# A copper-catalyzed asymmetric oxime propargylation enables the synthesis of the gliovirin tetrahydro-1,2-oxazine core†

**DOI:** 10.1039/d0sc04802j

**Published:** 2020-10-15

**Authors:** Nicholas G. W. Cowper, Matthew J. Hesse, Katie M. Chan, Sarah E. Reisman

**Affiliations:** The Warren and Katharine Schlinger Laboratory of Chemistry and Chemical Engineering, California Institute of Technology Pasadena CA 91125 USA reisman@caltech.edu

## Abstract

The bicyclic tetrahydro-1,2-oxazine subunit of gliovirin is synthesized through a diastereoselective copper-catalyzed cyclization of an *N*-hydroxyamino ester. Oxidative elaboration to the fully functionalized bicycle was achieved through a series of mild transformations. Central to this approach was the development of the first catalytic, enantioselective propargylation of an oxime to furnish a key *N*-hydroyxamino ester intermediate.

## Abstract 2

The bicyclic tetrahydro-1,2-oxazine subunit of gliovirin is synthesized through a diastereoselective copper-catalyzed cyclization of an *N*-hydroxyamino ester.

## Graphical abstract

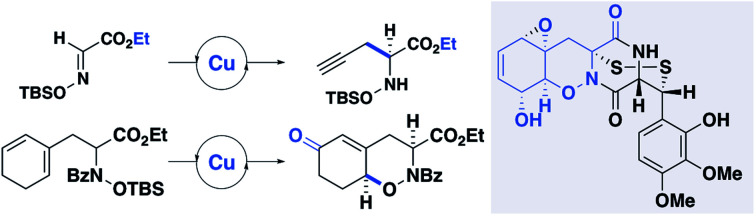

The fungal secondary metabolites gliovirin (**2**)^1^ and pretrichodermamides A (**3**)^2^ and E (**4**)^3^ are disulfide antibiotics that possess an unusual tetrahydro-1,2-oxazine (THO) core (Scheme 1). In addition to **2–4**, several related oxazine natural products have been isolated, including the monothiolated peniciadametizine B (**5**);^4^ however, these oxazine-containing natural products are rare relative to the biosynthetically related diketopiperazine natural products, hundreds of which have been isolated to date.^5^ In addition to their oxazine cores, **2–4** are unusual in that their disulfide linkages are joined to the carbon framework at C4 and C12, in contrast to the more common epipolythiodiketopiperazines (ETPs) such as gliotoxin (**1**).^6^ These fungal metabolites are proposed to be formed through thiolation of simple cyclic dipeptides followed by oxidative elaboration of the peripheral functionality.^7^ Perhaps because of the synthetic challenge posed by the combined oxazine and disulfide motifs, there have been no syntheses of gliovirin (**2**) or the related compounds **3** and **4** to date.

**Scheme 1:**
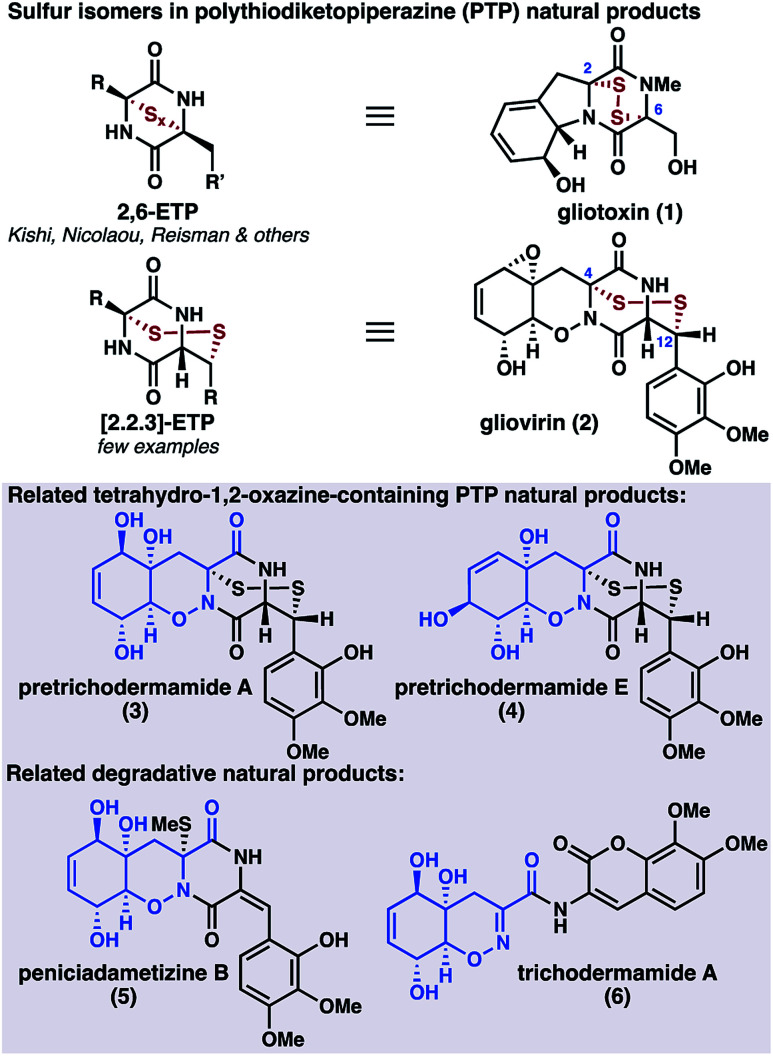
PTP isomerism: gliovirin and related natural products.

Whereas there are no syntheses of **2**, syntheses of related dihydro-1,2-oxazine (DHO) natural products, including trichodermamide A (**6**), have been reported by the groups of Joullié,^8^ Zakarian,^9^ and Larionov.^10^ These efforts relied upon cycloaddition chemistry or pericyclic rearrangement to install the DHO cores. As part of our larger program targeting the synthesis of polysulfide natural products,^11,12^ we envisioned a distinct approach to **2** that would involve late-stage diketopiperazine and disulfide formation, thereby reducing the synthetic challenge to that of preparing key THO **7** (Scheme 2). Oxazine **7** was expected to be accessible from **8a***via* epoxidation, desaturation, and functional group interconversion.

**Scheme 2:**
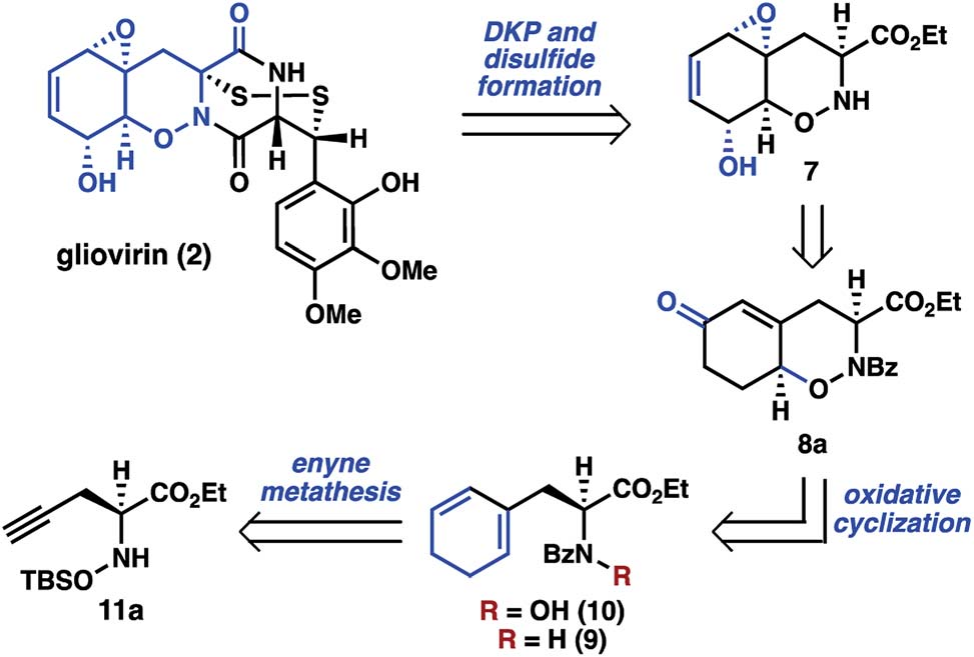
Retrosynthetic analysis of tetrahydro-1,2-oxazine **7**.

In a key synthetic step, the bicyclic THO **7** would be constructed by an intramolecular oxidative cyclization of *N*-hydroxydihydrophenylalanine derivative **10**. For the preparation of **10**, we considered two approaches: (1) *N*-oxidation of the corresponding dihydrophenylalanine **9**, or (2) initial installation of the N–O bond followed by construction of the cyclohexan-1,3-diene from the alkyne of **11a**. Given concerns about potential challenges of *N*-oxidation in the presence of the sensitive 1,3-cyclohexadiene motif, we elected to pursue a route where **10** would be accessed from α-propargyl *N*-hydroxyamino acid **11a** by an enyne metathesis reaction.

Having identified **11a** as an intermediate on route to **7**, a method to prepare this compound in enantioenriched form was desired. The most direct route to **11a** was envisioned to be an enantioselective propargylation of *N*-siloxyglyoxalate **12** (see Table 1). However, no examples of catalytic asymmetric addition of allyl nor propargyl nucleophiles to similar oxime substrates were found in the literature. The most promising lead was from Hanessian and coworkers, in which an excess of a chiral allylzinc reagent was added to an oxime.^13^ However, this method had not been extended to the corresponding propargylation.

**Table 1:** Optimization of Cu-catalyzed oxime propargylation^a^

Entry	B(OR^2^)_2_	[Cu], L	Yield^b^ (%)	ee^c^ (%)
1	Bgly (**13a**)	Cu(CO_2_*i*-Pr)_2_, **L1**^d^	2	63
2	Bgly (**13a**)	Cu(MeCN)_4_BF_4_, **L2**^d^	7	72
3	Bgly (**13a**)	Cu(MeCN)_4_BF_4_, **L3**^d^	70	30
4	Bgly (**13a**)	Cu(MeCN)_4_BF_4_, **L4**^d^	11	80
5	Bgly (**13a**)	Cu(MeCN)_4_BF_4_, **L5**^d^	24	82
6	Bgly (**13a**)	Cu(MeCN)_4_BF_4_, **L5**	30	95
7	Bgly (**13a**)	[Cu(**L5**)(MeCN)_2_]BF_4_	50	92
8	Bneo (**13b**)^e^	[Cu(**L5**)(MeCN)_2_]BF_4_	87	96

a Reactions conducted under inert atmosphere on 0.05 mmol scale for 24 h.

b Determined by ^1^H NMR *versus* an internal standard.

c Determined by SFC using chiral stationary phase.

d Li(O*t*-Bu) (9.5 mol%) was added to the reaction.

e 2.0 equivalents used in place of 1.4 equivalents.

Although there was no direct precedent for the catalytic asymmetric propargylation of oximes, we were inspired to pursue this approach by recent studies describing Cu-catalyzed asymmetric propargylation of imines.^14–16^ We began by investigating the ability of chiral Cu complexes to catalyze the reaction between glyoxalate-derived oxime **12b** and allenyl boronate **13a**. Bidentate bisphosphines gave promising levels of enantioinduction, although the reactions produced **11b** in very low yield (Table 1, entries 1–2).^17^ In comparison, monodentate phosphoramidite ligands (*e.g.***L3**) provided **11b** in improved yield, but with modest enantioselectivity (entry 3).

We hypothesized that the improved yield observed with the use of phosphoramidite ligands resulted from their increased ability to act as π-acceptors.^18^ It was envisioned that electron-deficient bis-phosphines would combine the benefits of greater π-acceptor ability to increase catalyst turnover while retaining the conformational rigidity of a bidentate ligand to promote asymmetric induction.^19^ Consistent with this hypothesis, fluorinated, commercially available, bisphosphines including DIFLUORPHOS (**L4**, entry 4) and BTFM-GARPHOS (**L5**, entry 5) both gave higher yields of **11b**, while also improving the enantioinduction.

In contrast to many metal-catalyzed cross-coupling reactions of boronates, a series of control experiments demonstrated that co-catalytic base was not required, and in fact, omitting base from the reaction led to an improvement in yield and ee (entry 6, Table 1). Use of neopentyl boronate **13b** further improved the yield. Although ester **12b** was used for the optimization process (due to the aryl UV chromophore aiding ee assay development), for the purpose of the synthesis, ethyl ester **11a** was accessed in similarly high yield and ee from *N*-siloxyglyoxalate **12a** (Scheme 3).

**Scheme 3:**
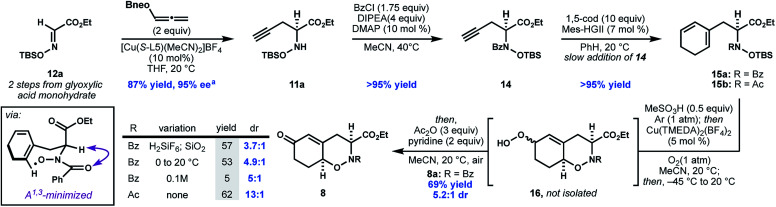
Realization of proposed oxidative cyclization. ^*a*^enantiomeric excess determined from **14**, following benzoylation, by SFC with a chiral stationary phase.

Concomitant to the development of the enantioselective propargylation shown in Table 1, we investigated the elaboration of compound **14**, as a racemate, to oxazine **7**. Initial attempts to generate the desired 1,3-cyclohexadiene **15a** through enyne metathesis proceeded in low yield due to catalyst deactivation and alkyne oligomerization; however, slow addition of **14** to a solution of 1,5-cyclooctadiene and second generation Hoveyda–Grubbs catalyst (Mes-HGII) in benzene produced the desired product, **15**, in excellent yield (Scheme 3).^20^

With access to *N*-hydroxydihydrophenylalanine derivative **15a**, we investigated the formation of the THO motif by an oxidative cyclization. The intramolecular oxidative radical addition of hydroxamic acids to generate cyclic hydroxamates was first observed by Perkins^21^ and later systematically studied by Alexanian.^22–24^ Furthermore, during the course of our work, the intermolecular addition of phthalimide *N*-oxyl radical (PINO) to activated alkenes was reported to be initiated by base metal catalysis, visible light, or conventional radical initiators.^25–28^ While this reactivity encouraged us, there were three issues that remained uncertain: (a) the regioselectivity of cyclization across the diene (*i.e.* 5-*exo vs.* 6-*endo*); (b) the diastereoselectivity of the C–O bond formation with respect the adjacent stereocenter; and (c) whether *N*-alkylhydroxamic acids would engage in similar reactivity previously observed in *N*-arylhydroxamic acids. With our cyclization substrate **15a** in hand we found that following *in situ* deprotection, silica-mediated autooxidation provided a mixture of allylic hydroperoxides **16** which could be converted to the corresponding enone **8** through a Kornblum–DeLaMare work-up.^29^ Under these conditions, the hydroxamic acid exhibits good selectivity for 6-*endo*-trig cyclization, presumably due to the stability of the intermediate allylic radical.

The desired *syn*-diastereomer **8a** was formed as the major product, albeit in modest diastereoselectivity. Eager to improve the dr, we screened a series of copper-diamine catalysts previously studied as copper monooxygenase mimics.^30^ To our delight, Cu(TMEDA)_2_(BF_4_)_2_ not only improved the diastereoselectivity, but also catalyzed the reaction at lower temperatures in higher combined yield of the 6*-endo* products.^31^ When an *N*-acetylhydroxamic acid (**8b**) is subjected to the optimal conditions, the dr improves to 13 : 1. The selectivity for the *syn* diastereomer in these reactions is consistent with related conformationally-controlled selectivity in cyclic amides,^32^ where the α-substituent adopts a pseudo-axial disposition to alleviate developing A^1,3^ strain in the chair-like transition state for cyclization.^33^ Although THO **8b** (R = Ac) was formed with higher diastereoselectivity, this compound was unstable to further elaboration. As a result, the more stable *N*-benzoyl THO **8a** was used for further elaboration to fully functionalized **22**.

With access to the desired bicyclic THO, our efforts turned to parlaying the newly installed enone to the oxidation pattern found in **2**. To our dismay, we found that traditional nucleophilic epoxidation conditions (*e.g.* NaOH, H_2_O_2_) led to complete decomposition of enone **8a**, while other oxidants, such as DMDO, returned starting material. After an extensive survey of the literature, we found promising reactivity using hydrogen peroxide and sodium bicarbonate, which presumably generates a peroxycarbonate species *in situ*.^34^ Further optimization found that use of sodium hypochlorite as the oxidant, in combination with catalytic CrCl_3_, provided **17** in good yield as a single diastereomer.

Initial attempts to desaturate epoxy ketone **17** by using classical Saegusa–Ito conditions or Tsuji-type oxidations of the corresponding silyl enol ethers were unsuccessful. In contrast, ketone **17** underwent smooth desaturation using conditions adapted from a recent report by White and coworkers,^35^ in which a Lewis acidic palladium catalyst enables *in situ* enolization and α-palladation. Under these conditions, epoxy enone **18** can be isolated directly in 67% yield.

At this stage, elaboration of **18** to **20b** was initially envisioned to proceed by diastereoselective ketone reduction followed by 1,3-transposition of the allylic alcohol (Scheme 4).^36^ Unfortunately, efforts to effect this strategy, or related approaches involving alkene formation and allylic oxidation, proved unsuccessful. As an alternate approach, we envisioned that a *bis*-epoxyketone (*i.e.***19**), which could potentially undergo chemoselective Wharton rearrangement to the desired allylic alcohol. To this end, treatment of enone **18** with sodium hypochlorite in 1,4-dioxane provided *bis*-epoxy enone **19** in high yield as a single diastereomer. Addition of 1.0 equiv. anhydrous hydrazine in the presence of catalytic benzoic acid with careful control of the temperature gave a mixture of isomers **20a** and **20b** in 33% yield. Unfortunately, efforts to further improve the efficiency of this reaction were unfruitful. Nonetheless, when the mixture of isomers was treated with the bulky, Lewis acidic silylating reagent TBSOTf, the corresponding secondary allylic silyl ether was as isolated exclusively.^37^ When unreacted **20a**, recovered from the reaction mixture, was subjected to neutral florisil purification a mixture of **20a** and **20b** were recovered. Taken together, these data might suggest that **20a** and **20b** can interconvert through an unusual vinylogous Payne rearrangement under Lewis acidic conditions.^38^ Finally, chemoselective cleavage of the *N*-benzoyl protecting group revealed **22**,^39^ our desired substrate for subsequent late stage diketopiperazine formation and thiolation.

**Scheme 4:**
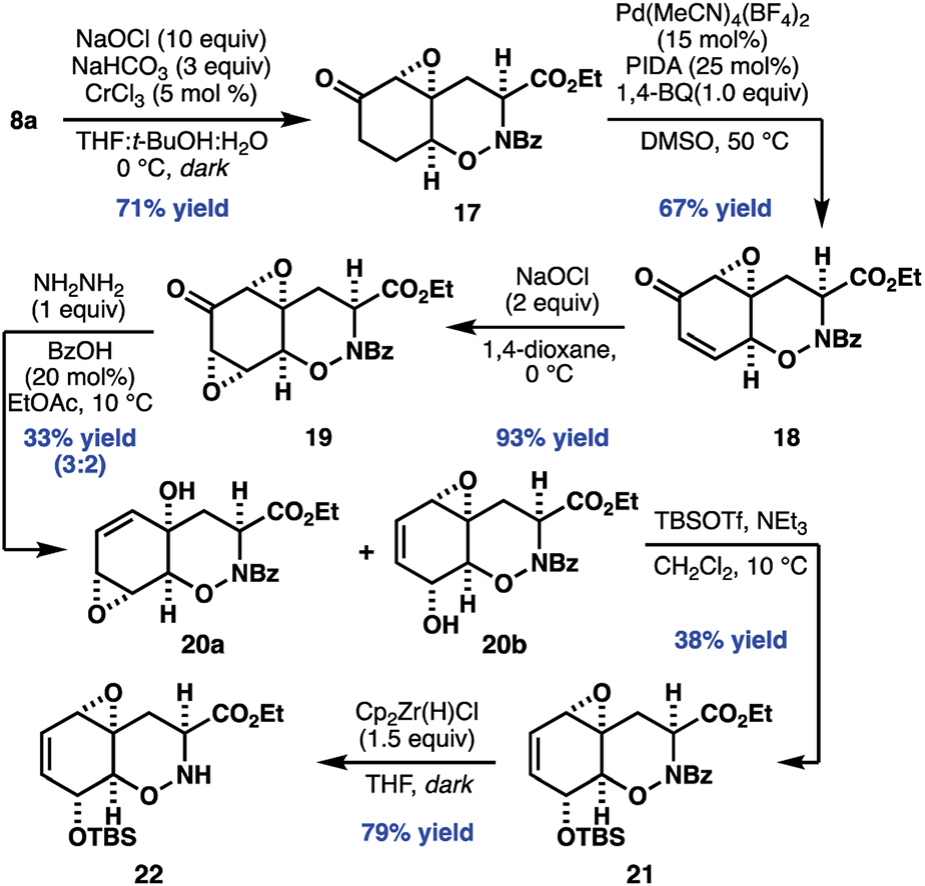
Synthesis of oxazine **22**.

## Conclusions

In conclusion, an approach to the bicyclic THO subunit of gliovirin (**22**) is reported. As part of these studies, a Cu-catalyzed asymmetric propargylation of a glyoxylate-derived oxime **12** was developed to prepare enantioenriched α-propargyl hydroxyamino acid **11**. The key oxazine motif was formed through a mild, Cu-catalyzed, intramolecular oxidative cyclization, which demonstrates that simple *N*-acyl hydroxamic acids can undergo a diastereoselective cyclization with dienes. Although the early introduction of the heterocyclic oxazine motif was initially viewed as an advantage of this synthetic approach, it ultimately proved to be a liability. In particular, compounds **17–22** were found to be acutely sensitive to base and acid, likely resulting from facile elimination to cleave the N–O bond.^40^ This study may serve to guide future work targeting the realization of the total synthesis of gliovirin and related THO natural products.

## Conflicts of interest

There are no conflicts to declare.

## Supplementary Material

SC-011-D0SC04802J-s001

SC-011-D0SC04802J-s002
