# Supplementary material for: A copper-catalyzed asymmetric oxime propargylation enables the synthesis of the gliovirin tetrahydro-1,2-oxazine core
Source: Chem Sci. 2020 Oct 15;11(43):11897–901. doi: 10.1039/d0sc04802j (PMC8162951; doi:10.1039/d0sc04802j)

Electronic Supplementary Material (ESI) for Chemical Science.
This journal is © The Royal Society of Chemistry 2020

Copper-catalyzed asymmetric oxime propargylation: enables the synthesis of the gliovirin tetrahydro-1,2-oxazine core

Nicholas G.W. Cowper, Matthew J. Hess, Katie M. Chan, and Sarah E. Reisman*

The Warren and Katharine Schlinger Laboratory of Chemistry and Chemical Engineering

Division of Chemistry and Chemical Engineering, California Institute of Technology, Pasadena, California 91125

SUPPORTING INFORMATION

TABLE OF CONTENTS **Page**

1. General Methods and Materials S2

2. Synthetic Procedures S4

3. Single Crystal X-ray Diffraction Data S25

4. ^{1}H and ^{13}C NMR Spectral Data S36

1. General Methods and Materials

Unless otherwise stated, reactions were performed under a nitrogen atmosphere using freshly dried solvents. Tetrahydrofuran (THF), methylene chloride CH_2Cl_2), acetonitrile (MeCN), dimethylformamide (DMF), benzene (PhH), diethyl ether (Et_2O) and toluene (PhMe) were dried by passing through activated alumina columns. Unless otherwise stated, chemicals and reagents were used as received. Triethylamine (Et_3N) was distilled over calcium hydride prior to use. All reactions were monitored by thin-layer chromatography using EMD/Merck silica gel 60 F254 pre- coated plates (0.25 mm) and were visualized by UV, *p*-anisaldehyde, vanillian, CAM or $KMnO_4$ staining. Flash column chromatography was performed either as described by Still *et al.* (W. C. Still, M. Kahn, A. Mitra, J. Org. Chem. 1978, 43, 2923.) using silica gel (partical size 0.032-0.063) purchased from Silicycle. Optical rotations were measured on a Jasco P-2000 polarimeter using a 100 mm path-length cell at 589 nm. ^{1}H and ^{13}C NMR spectra were recorded on a Bruker Avance III HD with Prodigy Cryoprobe (at 400MHz and 101 MHz, respectively), Varian 400 MR (at 400 MHz and 101 MHz, respectively), or a Varian Inova 500 (at 500 MHz and 126 MHz, respectively), and are reported relative to internal $CHCl_3$ (^{1}H, δ = 7.26), or DMSO (^{1}H, δ = 2.50), and $CDCl_3$ (^{13}C, δ = 77.0), or DMSO (^{13}C, δ = 40.0). Data for ^{1}H NMR spectra are reported as follows: chemical shift (δ ppm) (multiplicity, coupling constant (Hz), integration). Multiplicity and qualifier abbreviations are as follows: s =singlet, d = doublet, t = triplet, q = quartet, m = multiplet, br = broad, app = apparent. IR spectra were recorded on a Thermo Fisher Nicolet iS5 FTIR spectrometer and are reported in frequency of absorption (cm.$^{-1}$). HRMS were acquired using an Agilent 6200 Series TOF with an Agilent G1978A multimode source in electrospray ionization (ESI), atmospheric pressure chemical ionization (APCI), or mixed (MM) ionization mode. Analytical chiral SFC was performed with a Mettler SFC supercritical CO2analytical chromatography system (CO2= 1450 psi, column temperature = 40 °C) with Chiralcel AD-H, OD-H, AS-H, OB-H, and OJ-H columns (4.6 mm x 25 cm). Low-temperature X-ray diffraction data (φ-and ω-scans) were collected on a Bruker AXS D8 VENTURE KAPPA diffractometer coupled to a PHOTON 100 CMOS detector with Cu-Kα radiation (λ = 1.54178 Å) from an IμS micro-source.

2. Synthetic Procedures

*Preparation of ethyl ester **SI-2***

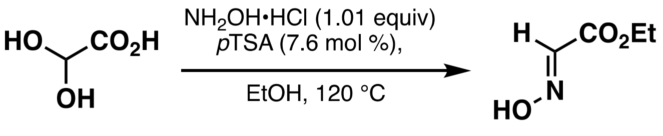

SI-1 **SI-2**

Charge a round bottom flask with glyoxylic acid monohydrate (**SI-1,** 20.0 g, 217 mmol 1.00 equiv), hydroxylamine hydrochloride (15.3 g, 220 mmol, 1.01 equiv), *p*TSA•H_2O (3.12 g, 16 mmol, 7.6 mol%) and ethanol (260 mL). Fit with a Socklett extractor charged with activated 4Å molecular sieves and a reflux condenser. Heat the mixture at 120°C for 9 hours. Cool reaction to room temperature. Concentrate in vacuo then dilute oil in Et_2O (400 mL) and $NaHCO_{3(sat)}$ (240 mL). Separate organic layer and wash organics with $NH_4Cl_{(sat)}$ (100mL) followed by pH=7 buffer (100 mL). Test aqueous layer for product and re-extract with Et_2O(150 mL), if necessary. Wash combined organics with brine (100 mL). Dry over Na_2SO_4, filter, and concentrate *in vacuo* to give clean **SI-2** (20.9 g, 174 mmol, 82% yield) as a pale yellow oil. Physical and spectral properties were consistent with literature values. (Mower, M. P.; Blackmond, D. G. *J. Am. Chem. Soc.* **2015**, *137* (6), 2386–2391.)

*Preparation of phenethyl ester **SI-3***

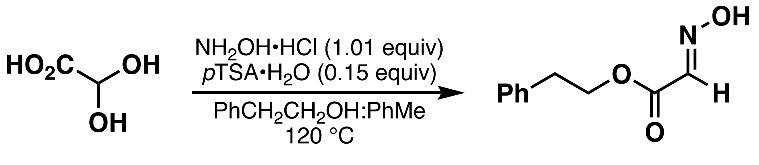

SI-1 **SI-3**

Combine glyoxylic acid monohydrate (**SI-1**, 3.00g, 32.59mmol, 1.0 equiv), hydroxylamine•HCl (2.29g, 32.92, 1.01 equiv), *p*-toluenesulfonic acid monohydrate (930.2mg, 4.89mmol 0.15 equiv) and phenethyl alcohol (11.7mL, 11.9g, 3.0 equiv) in toluene (10 mL). Heat mixture with a Dean-Stark trap to 50 °C and ramp to 120 °C over 80 min. Reflux overnight. Cool to ambient temperature, add EtOAc (100 mL). Wash organics layers with $NaHCO_{3(aq)}$ (100mL), then NH_4Cl (20 mL), then pH=7 buffer (20 mL) and finally

brine (40mL). Dry organics over Na_2SO_4. Purify by flash chromatography (silica, 300 g, 20→40% EtOAc/Hexanes) to yield S**I-3** (2.92g, 15.1 mmol, 46% yield) as a pale liquid.

^{1}H NMR (400 MHz, $CDCl_3$) δ 10.88 (s, 1H), 7.55 (d, J = 1.7 Hz, 1H), 7.35 – 7.27 (m, 2H), 7.27 – 7.17 (m, 3H), 4.45 (td, J = 7.2, 1.6 Hz, 2H), 3.06 – 2.94 (m, 2H).

^{13}C NMR (101 MHz, $CDCl_3$) δ 162.44, 141.47, 137.10, 128.87, 128.59, 126.75, 66.15, 34.77.

FTIR (AT-IR) 3322.11, 3028.17, 2359.63, 1721.19, 1622.35, 1497.30, 1453.99, 1306.73, 1257.07, 1193.59, 1009.49, 917.54, 744.20, 697.56, 667.93 cm^{-1}

HRMS (TOF, ES+) calc'd for $C_{10}H_{11}NO_3$ $[M+H]^+$ 194.0812, found 194.0819(ppm=-3.76)

*Preparation of silylated oxime **12a***

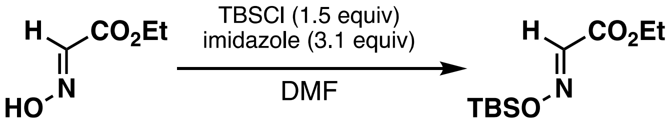

SI-2 **12a**

Combine **SI-2** (31.18g, 266 mmol), imidazole (55.76g, 819 mmol) and TBSCl (61.80g, 410 mmol) in DMF (210 mL). Stir at ambient temperature for 72h. Pour mixture into 6:1 DI:brine (2.1 L). Extract with Et_2O (1.5L). Wash organic layer with brine (300 mL). Dry over Na_2SO_4, filter and concentrate in vacuo to yield crude product. Purify by flash chromatography (silica, 3.5-4.5% Et_2O/Hexanes) to provide **12a** (47.8 g, 207 mmol, 78% yield) as a clear oil.

^{1}H NMR (400 MHz, $CDCl_3$) δ 7.59 (d, J = 1.5 Hz, 1H), 4.27 (qd, J = 7.1, 1.4 Hz, 2H), 1.31 (td, J = 7.1, 1.5 Hz, 3H), 0.92 (d, J = 1.9 Hz, 9H), 0.21 (d, J = 1.7 Hz, 6H).

^{13}C NMR (101 MHz, $CDCl_3$) δ 162.41, 146.20, 61.42, 25.91, 18.18, 14.22, -5.23.

FTIR (AT-IR) 2931.13, 2858.75, 1748.31, 1724.71, 1596.54, 1472.39, 1370.02, 1315.12, 1258.21, 1189.18, 1034.56, 967.56, 835.8, 785.55, 690.19, 667.95 cm^{-1}

HRMS (TOF, ES+) calc'd for $C_{20}H_{21}NO_3Si$ $[M+H]^+$ 232.1363, found 232.1365 (ppm=-0.66)

*Preparation of silylated oxime **12b***

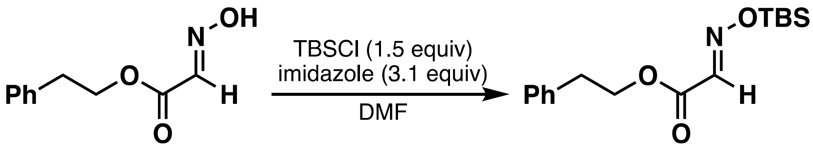

SI-3 (88% yield) 12b

Take up **SI-3** (566.5 mg, 2.93 mmol, 1.0 equiv) in DMF (5 mL). Add imidazole (618.2 mg, 9.08 mmol, 1.5 equiv) and TBSCl (663.2 mg, 4.40 mmol, 3.1 equiv) and stir at ambient temperature for 24h. Dilute in 6:1 DI H_2O:brine (26 mL) and extract with Et_2O (19 mL). Wash organic layer with brine (3.5 mL). Dry over Na_2SO_4, filter, and concentrate to yield the crude product. Purify by flash chromatography (silica, 3→5% EtOAc/Hexanes) to yield pure **12b** (795.1 mg, 2.59 mmol, 88% yield)

^{1}H NMR (400 MHz, $CDCl_3$) δ 7.60 (d, J = 1.2 Hz, 1H), 7.33 – 7.26 (m, 2H), 7.26 – 7.19 (m, 3H), 4.42 (td, J = 6.9, 1.3 Hz, 2H), 2.98 (t, J = 6.9 Hz, 2H), 0.97 (d, J = 1.7 Hz, 9H), 0.25 (d, J = 1.7 Hz, 6H).

^{13}C NMR (101 MHz, $CDCl_3$) δ 162.24, 145.88, 137.57, 129.05, 128.52, 126.67, 65.78, 35.01, 25.89, 18.14, -5.28.

FTIR (AT-IR) 2930.10, 2857.88, 1746.89, 1724.23, 1595.23, 1471.85, 1314.23, 1252.38, 1182.26, 1012.32, 974.59, 834.85, 784.87, 748.45, 697.31 cm^{-1}

HRMS (TOF, ES+) calc'd for $C_{16}H_{25}NO_3Si$ $[M+H]^+$ 308.1676, found 308.1676 (ppm=0.15)

*Synthesis of neopenylboronate **13b***

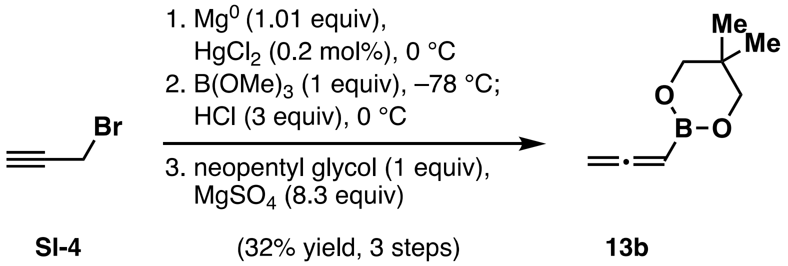

Adapted from: OL, 2011, p.4020; Org Syn. 1981, 60,41.

To freshly activated Mg^0 (6.11g, 251.3mmol, 1.01 equiv), add $HgCl_2$ (115mg, 0.425 mmol, 0.2 mol%) and suspend in Et_2O (50 mL). Propargyl bromide (29.79g, 250 mmol, 1.0 equiv) in PhMe (80 wt%, 27.8mL) was further diluted with additional Et_2O (170 mL). A small amount of the propargyl bromide solution (10 mL) was added to the suspension of Mg^0. Initiation was achieved through gentle heating of the resulting mixture. Cool in a salt/ice bath and add the remaining propargyl bromide solution as a slow, steady stream. After addition is complete, stir at ambient temperature for 1h. Cannulate the resulting Grignard solution, over 45 minute period, into a solution of freshly distilled trimethyl borate (26.0g, 27.9mL, 250 mmol, 1.0 equiv) in Et_2O (250 mL) cooled to –78 °C. After completion of Grignard addition, allow mixture to warm to ambient temperature. Cool the suspension once more to 0 °C and cannulate 3M $HCl_{(aq)}$ (250 mL, 3 equiv) dropwise into over 3h. Stir mixture until solids disappear, approximately 1h, and a further 20 minutes at ambient temperature. Separate the organic layer and wash the aqueous layer with Et_2O (3×150 mL); dry the combined organics over $MgSO_4$. Decant dried organics into a 2L round bottom flask and wash the remaining solids with dry Et_2O (100 mL). Concentrate solution under reduced pressure until the 500mL remain and backfill with argon. Add anhydrous $MgSO_4$ (250g, 2.08 mol, 8.31 equiv) and neopentyl glycol (26.04g, 250 mmol, 1.0 equiv). Rinse down solids with Et_2O (50 mL) and stir under argon with an overhead stirrer for 40h. Filter off solids using a large swivel frit. Take the caked solids and rinse with Et_2O (4×100 mL) through a packed sand filter. Recombine organic and remove solvent through a vacuum transfer. Add pentanes (400mL) to the remaining residue and cool to 0 °C. Filter off precipitated solids with a large swivel frit and remove pentane through a vacuum transfer. The remaining yellow oil is purified by kugelrohr distillation (90 °C/ 5.0 Torr → 110 °C/1.0 Torr) to yield **13b** (12.61g, 83.0 mmol, 32% yield) as a clear oil.

^{1}H NMR (400 MHz, $CDCl_3$) δ 4.82 (t, J = 7.0 Hz, 1H), 4.61 (d, J = 7.0 Hz, 2H), 3.66 (s, 4H), 0.98 (s, 6H).

^{13}C NMR (126 MHz, $CDCl_3$) δ 217.90, 72.47, 69.77, 31.84, 21.82.

^{11}B NMR (128 MHz, $CDCl_3$) δ 26.48.

FTIR (AT-IR) 2961.46, 2886.72, 1934.21, 1477.56, 1414.40, 1377.85, 1327.80, 1256.69, 1224.66, 1181.98, 1129.78, 812.31, 681.34, 667.05 cm^{-1}

*Small-scale enantioselective preparation of **11b***

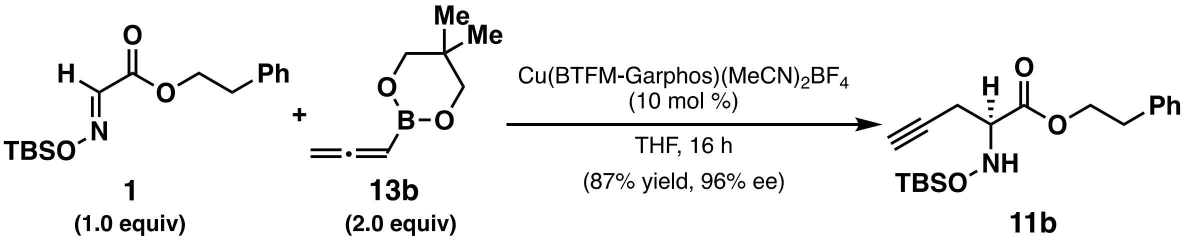

In a N_2-filled glovebox, dilute Cu(S-BTFMGarphos)$(MeCN)_2BF_4$ (28 mg, 0.020 mmol, 0.10 equiv) with THF (0.5 mL). Add **12b** (61.5 mg, 0.200 mmol) followed by **13b** (60.8 mg, 0.40 mmol, 2.0 equiv, 2 equiv) directly to the solution. Seal solution under N_2 and stir at ambient temperature for 16h. Dilute with EtOAc (2 mL) and diethanolamine (80 μL) and stir 15 minutes. Dilute with DI H_2O (3.0 mL) and extract with EtOAc (3×4.0 mL). Dry organics over Na_2SO_4, filter and concentrate *in vacuo* to yield crude product. ^{1}H NMR yields with dimethyl terephthalate (10 mol%) as a standard. Purification by flash chromatography. (silica, 2.5%Et_2O/ 10%CH_2Cl_2/10%PhMe/Hexanes) to yield **11b** (60.4 mg, 0.174 mmol, 87% yield) as a clear oil. The enantiomeric excess was determined to be 96% by chiral SFC analysis (AD, 2.5 mL/min, 1% IPA in CO_2, λ = 254 nm): t_R(minor) = 6.148 min, t_R(major) =5.116 min.

^{1}H NMR (400 MHz, $CDCl_3$): δ 7.33 – 7.28 (m, 2H), 7.24 (ddt, J = 7.6, 1.2, 0.6 Hz, 4H), 4.47 – 4.30 (m, 3H), 3.63 (t, J = 6.4 Hz, 1H), 2.98 (t, J = 7.1 Hz, 3H), 2.53 (tdd, J = 16.8, 6.4, 2.6 Hz, 2H), 2.04 – 1.93 (m, 1H), 0.89 (d, J = 0.5 Hz, 11H), 0.11 (d, J = 3.2 Hz, 6H).

^{13}C NMR (101 MHz, $CDCl_3$) δ 171.71, 137.73, 129.01, 128.64, 126.73, 79.34, 70.98, 65.68, 63.94, 35.19, 26.28, 19.41, 18.07, -5.38, -5.43.

FTIR (AT-IR) 3309.43, 2928.68, 2856.23, 1739.08, 1497.68, 1471.5, 1389, 1345.74, 1279.25, 1248.5, 1178.39, 1055.21, 974.31, 900.14, 833.73, 780.66, 747.8, 698.5, 644.51 cm^{-1}

HRMS (TOF, ES+) calc'd for $C_{19}H_{29}NO_3Si$ $[M+H]^+$ 348.1989, found 348.1998 (ppm=-2.45)

$[\alpha]_D^{23}$ −16.3 (c = 1.0, $CHCl_3$).

***rac*-11b**

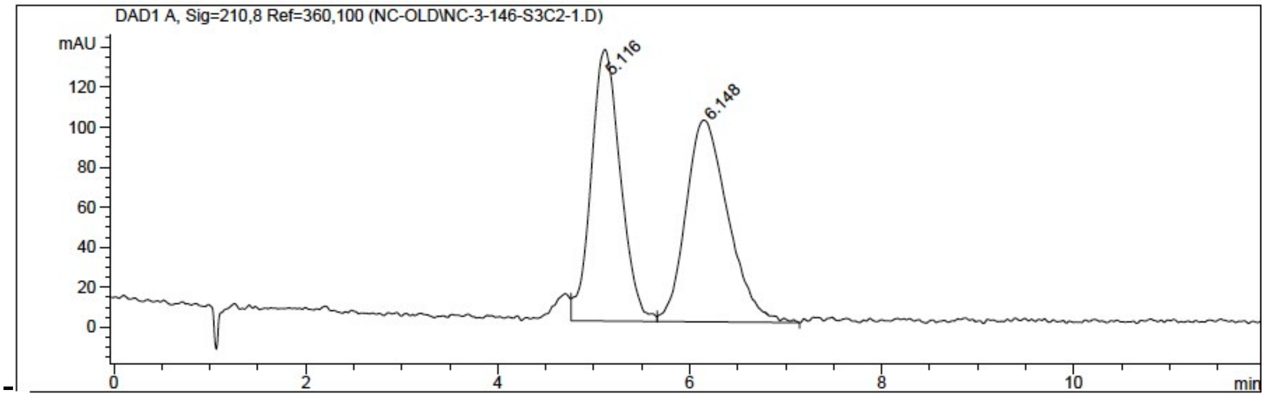

Peak #	RetTime [min]	Type	Width [min]	Area [mAU*s]	Height [mAU]	Area %
1	5.116	VV	0.3203	2889.85913	135.78705	48.4120
2	6.148	VV	0.4361	3079.43994	100.88213	51.5880

11b

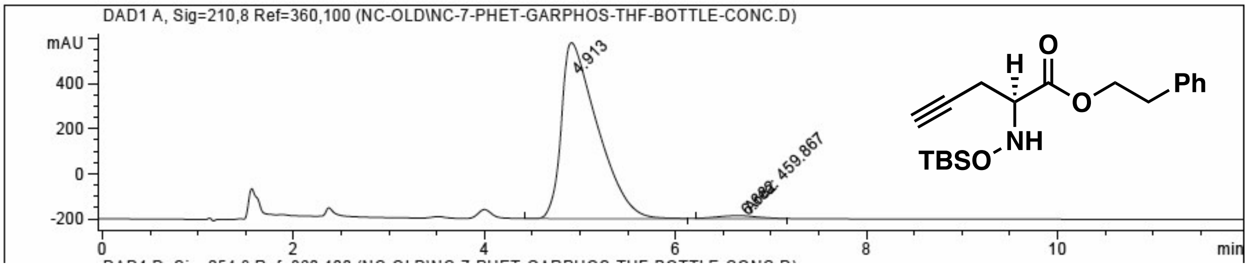

Peak #	RetTime [min]	Type	Width [min]	Area [mAU*s]	Height [mAU]	Area %
1	4.913	VB	0.3876	2.05818e4	780.59052	97.8145
2	6.682	MM	0.5361	459.86694	14.29552	2.1855

*Gram-scale enantioselective preparation of **11a***

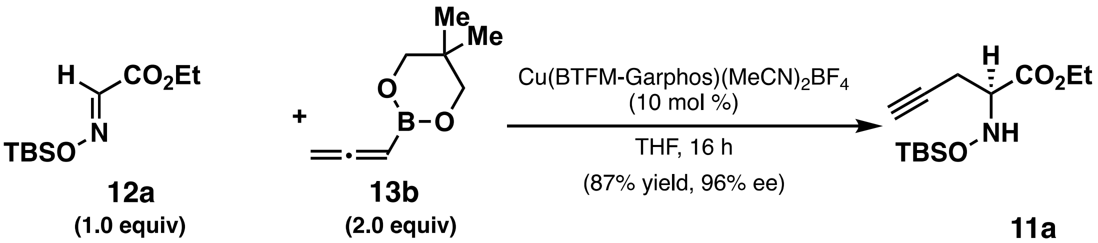

12a (1.0 equiv) **13b** (2.0 equiv) **11a**

Stir $Cu(MeCN)_4BF_4$ (135.9 mg, 0.432 mmol, 0.10 equiv) and *S*-BTFMGarphos (512.6 mg, 0.432 mmol, 0.10 equiv) in MeCN (4.0 mL) for 10 minutes before concentrating *in vacuo* to a yield a white powder. Dilute freshly prepared Cu(*S*-BTFMGarphos)$(MeCN)_2BF_4$ (0.432 mmol, 0.10 equiv) in THF (21.6 mL), add **12a** (1.00 g, 4.322 mmol, 1.0 equiv) and **13b** (295 mg, 1.944 mmol, 2.0 equiv). Stir at ambient temperature for 16h. Dilute with EtOAc (40 mL) and diethanolamine (3.2 mL) and stir 15 minutes. Dilute with DI H_2O (120 mL) and extract with EtOAc (3×150 mL). Dry organics over Na_2SO_4, filter and concentrate *in vacuo* to yield crude product. ^{1}H NMR yields with dimethyl terephthalate (10 mol%) as a standard. Purification by flash chromatography (silica, 2.5%Et_2O/10%CH_2Cl_2/10%PhMe/Hexanes) provided both **11a** (1.01 g, 3.76 mmol, 87% yield) and recovered ligand (450 mg, 88% recovery) Note: Any product fractions contaminated with ligand were concentrated and triturated with cold pentanes, before an azeotrope with PhMe. The enantiomeric excess was determined after benzoylation to be 95% by chiral SFC analysis.

^{1}H NMR (400 MHz, $CDCl_3$) δ 5.58 (s, 1H), 4.22 (q, *J* = 7.1 Hz, 2H), 3.60 (t, *J* = 6.4 Hz, 1H), 2.55 (dt, *J* = 6.4, 3.0 Hz, 2H), 2.00 (t, *J* = 2.7 Hz, 1H), 1.27 (td, *J* = 7.1, 0.7 Hz, 3H), 0.86 (d, *J* = 1.0 Hz, 9H), 0.08 (s, 6H).

^{13}C NMR (101 MHz, $CDCl_3$) δ 171.77, 79.37, 70.86, 63.89, 61.25, 26.21, 19.37, 18.01, 14.34, -5.43, -5.49.

FTIR (AT-IR) 3313.49, 2929.08, 2856.83, 2361.12, 2340.34, 1738.61, 1472.12, 1370.05, 1342.99, 1248.41, 1215.41, 1186.14, 1054.34, 904.00, 834.61, 780.54, 667.96 cm^{-1}

HRMS (TOF, ES+) calc'd for $C_{13}H_{25}NO_3Si$ $[M+H]^+$ 272.1676, found 272.1674 (ppm=0.91)

$[\alpha]_D^{23}$ –18.0° (c = 1.0, $CHCl_3$).

Preparation of N-benzyl **14**

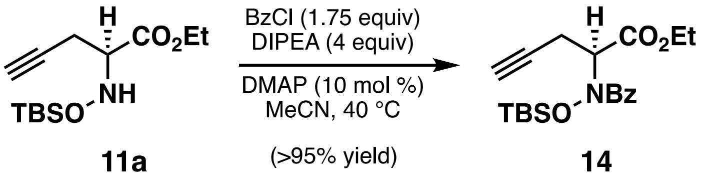

To a solution of **11a** (3.99 g, 14.70 mmol, 1.0 equiv) in MeCN (14.7 mL) add DIPEA (5.13 mL, 3.80 g, 29.40 mmol, 2.0 equiv) and benzoyl chloride (2.99 mL, 3.62 g, 25.73 mmol, 1.75 equiv) at ambient temperature and stir 30 minutes. Heat mixture to 40 °C stirring vigorously for 7 hours. Dilute in Et_2O (140 mL) and wash organics with with pH=7 phosphate buffer (30 mL), then brine (30 mL). Dry organic layer with Na_2SO_4, filter through celite, and concentrate. Purify by flash chromatography (florisil, 20% Et_2O/Hexanes) to yield **14** (5.5g, 14.65 mmol, >95% yield) as pale white crystals.

SFC analysis (IC, 5% *i*-PrOH in CO_2) peak 1(major): 6.748 min; peak 2(minor): 8.324 min; 95% ee

^{1}H NMR (400 MHz, $CDCl_3$) δ 7.76 – 7.65 (m, 2H), 7.53 – 7.35 (m, 3H), 4.65 (dd, J = 10.5, 4.5 Hz, 1H), 4.21 (qdd, J = 10.7, 7.0, 3.6 Hz, 2H), 2.97 (ddd, J = 17.4, 10.6, 2.7 Hz, 1H), 2.89 – 2.70 (m, 1H), 2.14 (t, J = 2.7 Hz, 1H), 1.30 (t, J = 7.1 Hz, 3H), 0.95 (s, 9H), 0.30 (s, 3H), 0.21 (s, 3H).

^{13}C NMR (101 MHz, $CDCl_3$) δ 173.13, 168.28, 134.61, 131.05, 128.59, 128.48, 80.35, 71.70, 63.97, 62.11, 26.18, 18.58, 14.31, -4.31, -4.51.

FTIR (AT-IR) 3310.03, 2929.56, 2857.22, 2359.18, 1744.02, 1694.62, 1472.02, 1446.93, 1390.26, 1362.25, 1289.85, 1250.00, 1226.16, 1186.10, 1072.43, 1017.66, 964.62, 920.36, 831.69, 809.32, 783.13, 748.13, 703.47, 674.24, 654.39 cm^{-1}

HRMS (TOF, ES+) calc'd for $C_{20}H_{30}NO_4Si$ $[M+H]^+$ 376.1939, found 376.1934

$[\alpha]_D^{23}$ –89.9° (c=1.0, $CHCl_3$)

***rac*-14**

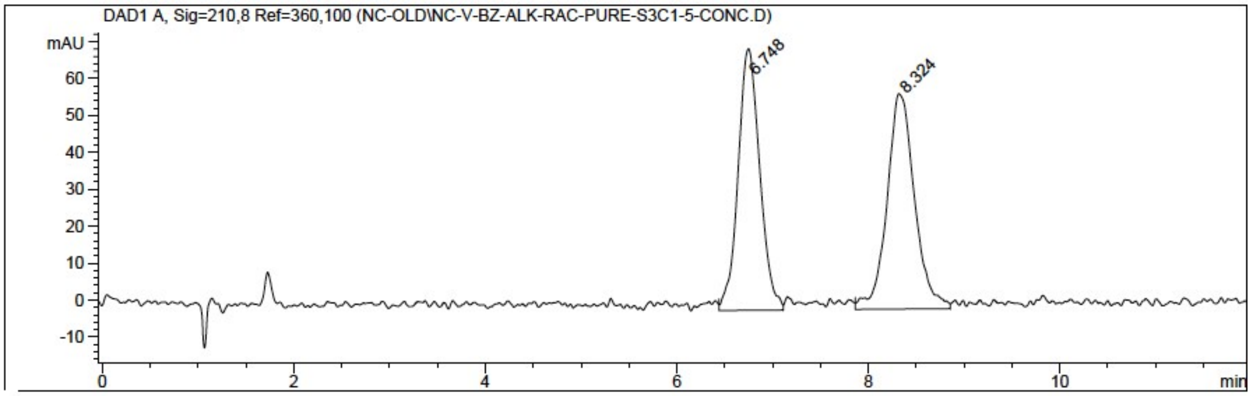

Peak #	RetTime [min]	Type	Width [min]	Area [mAU*s]	Height [mAU]	Area %
1	6.748	VV	0.2318	1116.34790	70.85162	48.8551
2	8.324	VV	0.2857	1168.67224	58.33414	51.1449

14

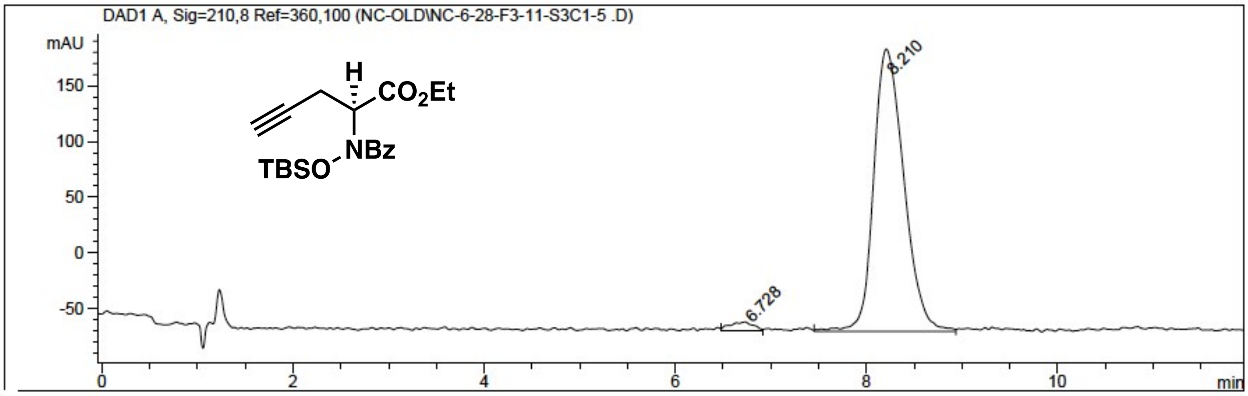

Peak #	RetTime [min]	Type	Width [min]	Area [mAU*s]	Height [mAU]	Area %
1	6.728	VV	0.2246	147.39131	8.40454	2.5675
2	8.210	VV	0.3533	5593.31201	253.61139	97.4325

*Racemic preparation of **11a***

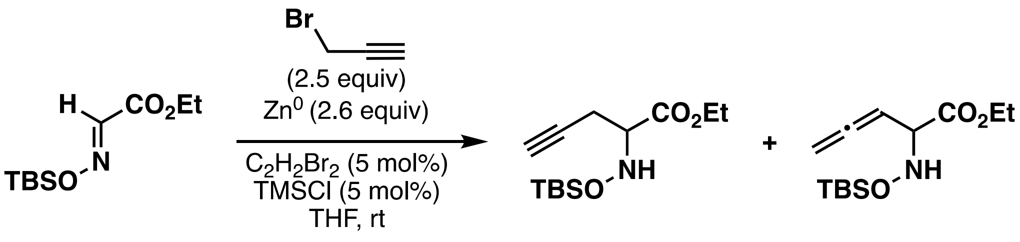

Suspend dry Zn^0 (14.13 g, 224.74 mmol, 2.6 equiv) in THF (400 mL). Add 1,2-dibromoethane (0.37 mL, 812 mg, 4.32 mmol, 5 mol %) and TMSCl (0.55 mL, 469 mg, 4.32 mmol, 5 mol %), stir at room temperature for 45 min. Add a solution of propargyl bromide, 80%wt in PhMe, (0.20 mL, 1.80 mmol, 2 mol %). Heat gently until initiation is observed. Add the remainder of propargyl bromide, 80 wt% in PhMe, (23.9 mL, 214.57 mmol, 2.58 equiv) dropwise. With addition complete, stir 30 min at ambient temperature vigorously, until zinc is no longer consumed. Cannulate fresh organozinc into a solution of **12a** (20.00 g, 86.44 mmol, 1.0 equiv) in THF (400 mL) chilled to 0 °C, over a three hour period. Upon disappearance of starting material quench with $NaHCO_{3(sat)}$ (200 mL). Filter off salts through a sand pad. Wash salts Et_2O(3×200 mL). Wash combined organics with 1:1 DI H_2O:brine then brine. Dry over Na_2SO_4, filter and concentrate in vacuo to yield crude product. Purification by flash chromatography (silica 500g, 5% EtOAc/Hexanes) yields *rac*-**11a** (15.1g, 55.6 mmol, 64% yield) as a pale yellow oil. Trace allene **SI-5** was also isolated for characterization.

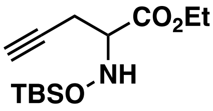

rac-**11a**

^{1}H NMR (400 MHz, $CDCl_3$) δ 5.60 (d, J = 9.5 Hz, 1H), 4.24 (q, J = 7.1 Hz, 2H), 3.79 – 3.46 (m, 1H), 2.57 (dt, J = 6.4, 2.8 Hz, 2H), 2.02 (t, J = 2.7 Hz, 1H), 1.29 (t, J = 7.1 Hz, 3H), 0.89 (s, 9H), 0.11 (d, J = 1.2 Hz, 6H).

^{13}C NMR (101 MHz, $CDCl_3$) δ 171.77, 79.37, 70.86, 63.89, 61.25, 26.21, 19.37, 18.01, 14.34, -5.43, -5.49.

FTIR (AT-IR) 3313.49, 2929.08, 2856.83, 2361.12, 2340.34, 1738.61, 1472.12, 1370.05, 1342.99, 1248.41, 1215.41, 1186.14, 1054.34, 904.00, 834.61, 780.54, 667.96 cm^{-1}

HRMS (TOF, ES+) calc'd for $C_{13}H_{26}NO_3$ $[M+H]^+$ 272.1676, found 272.1673(ppm=-1.28)

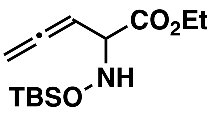

SI-5

^{1}H NMR (400 MHz, $CDCl_3$) δ 5.48 (d, *J* = 10.6 Hz, 1H), 5.12 (q, *J* = 6.8 Hz, 1H), 4.80 (dd, *J* = 6.7, 2.5 Hz, 2H), 4.18 (qd, *J* = 7.1, 0.9 Hz, 2H), 3.98 (ddt, *J* = 9.8, 7.0, 2.3 Hz, 1H), 1.23 (t, *J* = 7.1 Hz, 3H), 0.84 (d, *J* = 1.1 Hz, 9H), 0.05 (d, *J* = 1.0 Hz, 6H).

^{13}C NMR (101 MHz, $CDCl_3$) δ 209.30, 171.64, 85.51, 77.47, 64.67, 64.64, 61.08, 26.19, 26.15, 26.13, 17.96, 14.34, 14.30, -5.49, -5.53, -5.57.

FTIR (AT-IR) 2929.12, 2856.96, 1957.66, 1741.62, 1472.28, 1390.00, 1368.67, 1301.88, 1247.48, 1183.61, 1043.73, 832.33, 779.68, 666.29 cm^{-1}

HRMS (TOF, ES+) calc'd for $C_{11}H_{25}NO_{3Si}$ $[M+H]^+$ 272.1676, found 272.1686 (ppm=3.67)

Preparation of N-acetyl alkyne **SI-5**

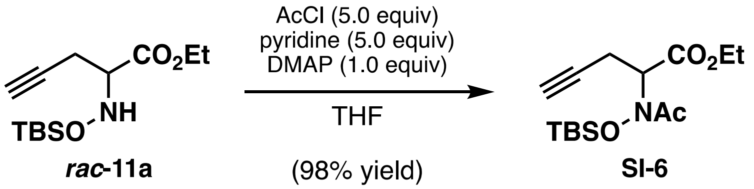

Add pyridine (1.48 mL, 1.46 g, 18.42 mmol, 5.0 equiv) and DMAP (450 mg, 3.83 mmol, 0.96 equiv) to solution of ***rac*-11a** (1.04 g, 3.83 mmol, 1.0 equiv) in THF (60 mL). Add acetyl chloride (1.31 mL, 1.45 g, 18.42 mmol, 5.0 equiv) and stir vigorously at ambient temperature for 18h. Dilute in Et_2O (50 mL) and wash with pH=7 phosphate buffer (50mL) then brine (50 mL). Dry organic phase over Na_2SO_4, filter and concentrate gave **SI-6** (1.18 g, 3.76 mmol, 98% yield) as a pale-yellow oil with no further purification necessary.

^{1}H NMR (400 MHz, $CDCl_3$) δ 4.56 (t, *J* = 7.5 Hz, 1H), 4.21 (q, *J* = 7.1 Hz, 2H), 3.01 – 2.83 (m, 2H), 2.16 (s, 3H), 2.02 (t, *J* = 2.7 Hz, 1H), 1.27 (t, *J* = 7.1 Hz, 3H), 0.96 (s, 9H), 0.29 (s, 3H), 0.24 (s, 3H).

^{13}C NMR (126 MHz, $CDCl_3$) δ 176.42, 168.14, 80.35, 70.42, 63.61, 61.72, 25.80, 21.52, 18.68, 17.93, 14.08, -4.51, -4.76.

FTIR (AT-IR) 3282.17, 2930.95, 2858.52, 2361.30, 1742.64, 1674.63, 1472.78, 1463.68, 1367.92, 1253.56, 1185.00, 1080.68, 1018.10, 983.77, 965.14, 938.25, 833.87, 784.10, 667.99 cm^{-1}

HRMS (TOF, ES+) calc'd for $C_{15}H_{27}NO_4Si$ $[M+H]^+$ 314.1782, found 314.1780 (ppm=0.67)

Preparation of N-benzyl cyclohexadiene **15a**

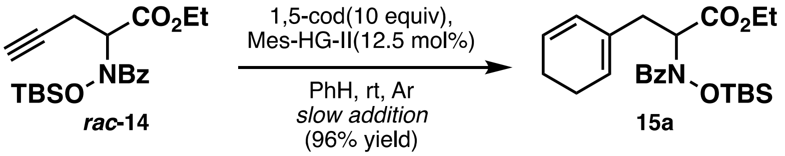

Solvate Mes-HG-II (692 mg, 1.103 mmol, 7.5 mol%) stored in the glovebox in benzene (55 mL). Maintain an Ar atmosphere. Add distilled and degassed 1,5-cyclooctadiene (18.0 mL, 15.90 g, 147.0 mmol, 10 equiv), stir five minutes. Concurrently, both a solution of ***rac*-14** (5.50 g, 14.70 mmol, 1.0 equiv) in benzene (360 mL) and a solution of Mes-HG-II (461 mg, 0.735 mmol, 5 mol%) in benzene (10.6 mL) were added by syringe pumps over 12h. Stir at room temperature for 2h. Concentrate the crude reaction onto celite (50 g) overnight. Purify by flash chromatography (silica 150 g, 10→20% Et_2O/Hexanes). Concentrate to an oil and dilute in cold MeCN (50 mL). Filter off precipitate with celite and wash the celite pad twice with cold MeCN (50 mL). Concentrate to yield **15a** (6.09 g, 14.2 mmol, 96% yield) a beige oil.

^{1}H NMR (400 MHz, $CDCl_3$) δ 7.56 (dt, J = 6.8, 1.5 Hz, 2H), 7.45 – 7.37 (m, 1H), 7.37 (s, 2H), 5.79 (dd, J = 9.5, 4.3 Hz, 1H), 5.70 – 5.54 (m, 2H), 4.45 (dd, J = 10.1, 4.4 Hz, 1H), 4.32 – 4.13 (m, 3H), 2.85 (dd, J = 14.2, 10.1 Hz, 1H), 2.60 (d, J = 14.1 Hz, 1H), 2.15 – 2.09 (m, 4H), 1.33 (t, J = 7.1 Hz, 4H), 0.92 (s, 10H), 0.29 (s, 3H), 0.13 (s, 3H).

^{13}C NMR (101 MHz, $CDCl_3$) δ 170.80, 169.47, 134.72, 131.16, 130.60, 129.83, 128.26, 127.25, 126.70, 124.77, 64.74, 61.74, 33.61, 26.19, 22.55, 22.19, 18.87, 14.29, -3.86, -4.52.

FTIR (AT-IR) 2929.77, 2857.11, 2359.5, 2340.28, 1742.71, 1653.06, 1471.97, 1447.06, 1249.13, 1183.09, 1019.33, 969.71, 919.13, 826.95, 783.52, 735.42, 700.25, 667.9 cm^{-1}

HRMS (TOF, ES+) calc'd for $C_{24}H_{35}NO_4Si$ $[M+H]^+$ 430.2408, found 430.2395 (ppm=3.05)

Preparation of N-acetyl cyclohexadiene **15b**

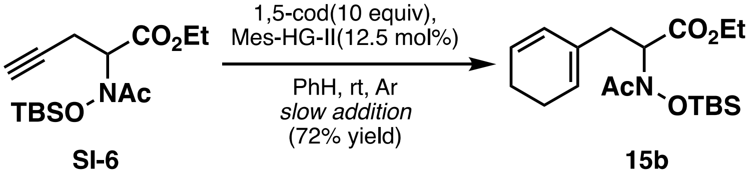

Solvate Mes-HG-II (176.7 mg, 0.282 mmol, 7.5 mol%) stored in the glovebox in benzene (14 mL). Maintain an Ar atmosphere. Add distilled and degassed 1,5-cyclooctadiene (4.62 mL, 4.07g, 37.64 mmol, 10 equiv), stirring vigorously (700 rpm). Concurrently, both a solution of **SI-6** (1.18g, 3.76mmol, 1 equiv) in benzene (90 mL) and a solution of Mes-HG-II (117.8 mg, 0.188 mmol, 5 mol%) in benzene (3.5 mL) were added by syringe pumps over 10h. Stir at room temperature for 3h. Concentrate the crude reaction onto celite overnight. Purify by flash chromatography (silica, 7.5→10% EtOAc/Hexanes). Add $P(CH_2CH_2OH)_3$ (1.16g, 20 equiv) to product-containing fractions along with silica and was sonicate until the combined solution is clear. Filter the solution was then and concentrate to yield **15b** (991mg, 2.70 mmol, 72% yield) a clear oil.

^{1}H NMR (500 MHz, $CDCl_3$) δ 5.81 (d, J = 1.3 Hz, 2H), 5.58 (d, J = 4.5 Hz, 1H), 4.47 (s, 1H), 4.26 – 4.08 (m, 2H), 2.78 (ddd, J = 14.2, 10.3, 0.9 Hz, 1H), 2.65 (dd, J = 14.4, 4.8 Hz, 1H), 2.10 – 2.04 (m, 7H), 1.27 (t, J = 7.1 Hz, 3H), 0.94 (s, 9H), 0.26 (s, 3H), 0.18 (s, 3H).

^{13}C NMR (101 MHz, $CDCl_3$) δ 175.10, 169.64, 131.84, 127.11, 126.84, 123.88, 64.01, 61.53, 33.64, 25.97, 22.52, 22.24, 21.62, 18.07, 14.22, -4.53.

FTIR (AT-IR) 2930.65, 2857.62, 2359.56, 2340.27, 1742.48, 1667.8, 1367.59, 1252.92, 1031.68, 832.83, 783.93, 667.92 cm^{-1}

HRMS (TOF, ES+) calc'd for $C_{19}H_{33}NO_4Si$ $[M+H]^+$ 368.2252, found 368.2253 (ppm=–0.38)

*Preparation of N-benzyl THO enone **8a***

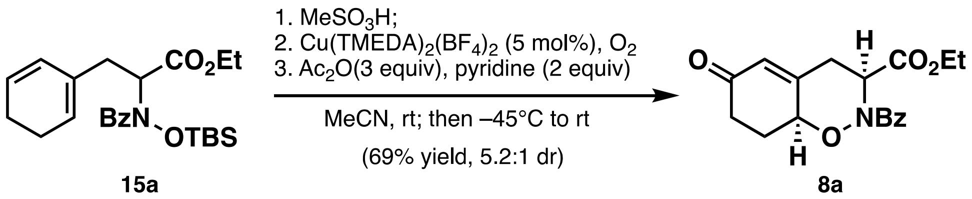

Add $MeSO_3H$ (0.23mL, 325mg, 3.49 mmol, 0.5 equiv) to **15a** (3.00 g, 6.98 mmol, 1.00 equiv) under argon in wet MeCN (350 mL) and begin cool to –35 °C. Sparge reaction with O_2 and add $Cu(TMEDA)_2(BF4)_2$ (0.0349 mmol, 0.05 equiv) as a solution in MeCN (1 mL). [Note: $Cu(TMEDA)_2(BF4)_2$ made by dissolving $Cu(BF_4)_2$•xH_2O (20 wt% Cu) (111 mg, 0.349 mmol, 0.05 equiv) and TMEDA (0.10 mL, 81 mg, 0.10 equiv) in MeCN(1 mL)]. Continue cooling to –45 °C. Stop O_2 sparge after 10 minutes at –45 °C. Slowly allow reaction return to room temperature. Add acetic anhydride (3.84 mL, 4.16 g, 40.48 mmol, 6.0 equiv), stir 1 minute then add pyridine (0.54 mL, 534 mg, 6.75 mmol, 1 equiv) allow to stir under air overnight. Dilute with EtOAc (350 mL) wash with an aqueous solution (357 mL) composed of EDTA pH=9 buffer (7 mL), DI H_2O (175mL) and brine (175mL). Wash organic with additional brine (50 mL). Extract combined aqueous layer with EtOAc (2x100mL). Dry combined organic layers over Na_2SO_4, filter and concentrate. Purify by flash chromatography (silica 215 g, 40% EtOAc/Hexanes) to yield **8a** and ***anti*-8a** as an overall 5.2:1 dr mixture (1.512g, 4.59 mmol, 69% yield).

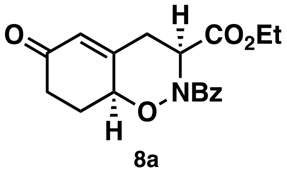

8a: ^{1}**H NMR** (400 MHz, $CDCl_3$) δ 7.78 (d, J = 7.6 Hz, 2H), 7.57 – 7.49 (m, 1H), 7.45 (ddt, J = 8.3, 6.6, 1.3 Hz, 2H), 6.02 (d, J = 2.3 Hz, 1H), 5.54 (s, 1H), 4.62 (s, 1H), 4.26 (q, J = 7.2 Hz, 2H), 3.16 (d, J = 15.7 Hz, 1H), 2.96 (dd, J = 15.8, 7.0 Hz, 1H), 2.51 (d, J = 16.7 Hz, 1H), 2.30 (td, J = 16.5, 15.9, 5.0 Hz, 1H), 2.13 (d, J = 23.1 Hz, 1H), 1.97 – 1.80 (m, 1H), 1.29 (t, J = 7.2 Hz, 4H).

^{13}**C NMR** (101 MHz, $CDCl_3$) δ 196.87, 170.20, 168.39, 153.52, 132.54, 131.83, 128.94, 128.27, 127.95, 78.86, 77.52, 62.33, 35.05, 31.33, 29.87, 26.33, 14.33.

FTIR (AT-IR) 2979.74, 2359.59, 1738.00, 1667.46, 1600.60, 1578.10, 1447.41, 1387.71, 1364.39, 1316.29, 1254.3, 1197.39, 1139.88, 1077.16, 1027.25, 975.89, 899.81, 871.89, 789.33, 758.51, 706.03, 617.25 cm^{-1}

HRMS (TOF, ES+) calc'd for $C_{18}H_{19}NO_5$ $[M+H]^+$ 330.1336, found 330.1337 (ppm=-0.31)

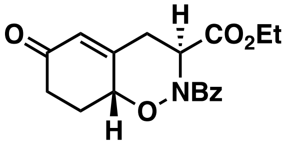

anti-8a

***anti*-8a:** ^{1}**H NMR** (400 MHz, CDCl3) δ 7.80 (dt, *J* = 8.5, 1.6 Hz, 2H), 7.53 – 7.46 (m, 1H), 7.46 – 7.38 (m, 2H), 6.02 – 5.93 (m, 1H), 4.99 (dd, *J* = 9.6, 7.6 Hz, 1H), 4.76 (t, *J* = 8.3 Hz, 1H), 4.36 – 4.22 (m, 2H), 3.14 – 2.96 (m, 2H), 2.53 – 2.44 (m, 1H), 2.33 – 2.14 (m, 1H), 1.89 (ddd, *J* = 9.7, 8.0, 5.0 Hz, 2H), 1.35 – 1.29 (m, 3H).

^{1}**H NMR** (500 MHz, $CDCl_3$) δ 7.82 – 7.77 (m, 2H), 7.52 – 7.46 (m, 1H), 7.45 – 7.39 (m, 2H), 6.01 – 5.96 (m, 1H), 4.98 (dd, *J* = 9.6, 7.5 Hz, 1H), 4.81 – 4.68 (m, 1H), 4.28 (q, *J* = 7.1 Hz, 2H), 3.10 – 2.95 (m, 2H), 2.49 (dddd, *J* = 17.2, 4.4, 2.9, 1.2 Hz, 1H), 2.31 – 2.19 (m, 1H), 1.97 – 1.80 (m, 2H), 1.31 (t, *J* = 7.1 Hz, 3H).

^{13}**C NMR** (101 MHz, $CDCl_3$) δ 196.64, 170.15, 168.93, 157.09, 132.65, 131.60, 129.22, 128.10, 127.77, 80.96, 62.26, 58.14, 35.41, 30.86, 27.55, 14.28.

FTIR (AT-IR) 2359.53, 2340.23, 1729.8, 1637.97, 1577.29, 1448.88, 1394.9, 1300.99, 1245.96, 1199.42, 1098.44, 1008.47, 981.15, 962.42, 904.15, 844.43, 790.7, 736.73, 707.82, 667.97, 635.45 cm^{-1}

HRMS (TOF, ES+) calc'd for $C_{18}H_{19}NO_5$ $[M+H]^+$ 330.1336, found 330.1334 (ppm=0.60)

Preparation of N-acetyl THO enone ***8b***

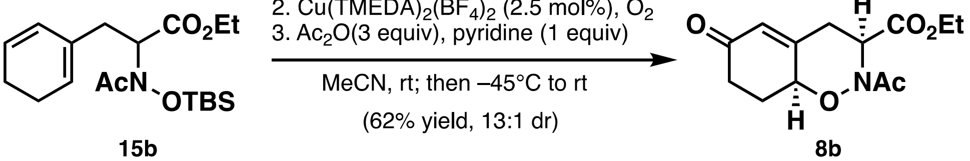

15b 8b

Add $MeSO_3H$ (18 μL, 26.1 mg, 0.272 mmol, 0.5 equiv) in MeCN (0.5 mL) to **15b** (200 mg, 0.544 mmol, 1.0 equiv) under argon in wet MeCN (26 mL) and begin cool to –35 °C. Sparge reaction with O_2 and add $Cu(TMEDA)_2(BF4)_2$ (0.00.136 mmol, 0.05 equiv) as a solution in MeCN (0.5 mL). Note: $Cu(TMEDA)_2(BF4)_2$ made by dissolving $Cu(BF_4)_2{\bullet}xH_2O$ (20 wt% Cu) (4.3 mg, 0.0.0136 mmol, 0.05 equiv) and TMEDA (4 μL, 3.2 mg, 0.0272 mmol, 0.10 equiv) in MeCN (0.5 mL). Continue cooling to –45 °C. Stop O_2 sparge after 10 minutes but continue stirring at –45 °C for 2h. Slowly allow reaction return to room temperature. Add acetic anhydride (0.16 mL, 168 mg, 1.632 mmol, 3.0 equiv), stir 1 minute then add pyridine (44 μL, 43 mg, 0.544 mmol, 1 equiv) allow to stir under air overnight. Dilute with EtOAc (20 mL) wash with an aqueous solution (35 mL) composed of EDTA pH=9 buffer (10 mL), DI H_2O (5mL) and brine (5 mL). Extract combined aqueous with EOAc (10 mL) Wash combined organics with additional brine (20 mL). Dry combined organic layers over Na_2SO_4, filter and concentrate. Purify crude material by flash chromatography on silica with 100% Et_2O then 75% to 100%EtOAc/Hexanes. Recovered 90 mg (0.337 mmol, 62% yield) of **8b** as a mixture of diastereomers (13:1 dr).

^{1}H NMR (600 MHz, $CDCl_3$) δ 6.01 (q, *J* = 1.5 Hz, 1H), 5.41 (dd, *J* = 7.2, 1.4 Hz, 1H), 4.68 (dd, *J* = 10.7, 5.1 Hz, 1H), 4.27 – 4.15 (m, 2H), 3.11 (ddt, *J* = 15.7, 1.5, 0.7 Hz, 1H), 2.84 (dddd, *J* = 15.7, 7.2, 2.5, 1.6 Hz, 1H), 2.63 – 2.55 (m, 1H), 2.41 – 2.33 (m, 2H), 2.25 (s, 3H), 1.98 (dddd, *J* = 16.2, 14.1, 9.0, 3.3 Hz, 1H), 1.26 (td, *J* = 7.1, 1.0 Hz, 4H).

^{13}C NMR (126 MHz, $CDCl_3$) δ 196.79, 171.44, 168.28, 153.41, 127.93, 78.79, 62.19, 53.60, 35.02, 31.50, 26.38, 20.30, 14.26.

FTIR (AT-IR) 2931.38, 2360.54, 1737.83, 1668.55, 1402.03, 1368.95, 1314.81, 1256.65, 1198.38, 1026.43, 975.46, 945.41, 900.55, 725.65 cm^{-1}

HRMS (TOF, ES+) calc'd for $C_{13}H_{17}NO_5$ $[M+H]^+$ 268.1179, found 268.1180 (ppm=-0.19)

*Preparation of epoxy ketone **17***

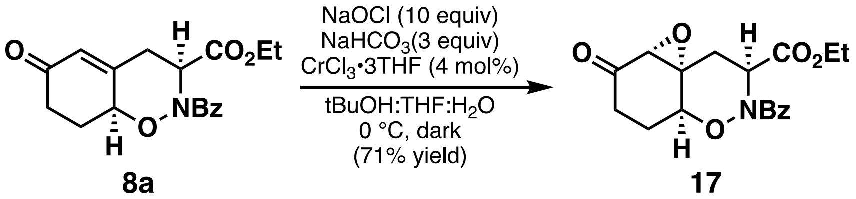

Substrate **8a** (726.8mg, 2.207 mmol, 1 equiv) was dissolved in THF (37 mL) and pH=7 phosphate buffer (11 mL), reaction is kept dark, cooled to 0 °C. Cannulate suspension of $CrCl_3$•3THF (33mg, 0.088 mmol, 0.04 equiv) and $NaHCO_3$ (556 mg, 6.620 mmol, 3.0 equiv) in THF (74 mL) and H_2O (33 mL) dropwise. After 75 minutes, reaction is complete. Add 0.20M sodium thiosulfate and pH=7 phosphate buffer, Extract with CH_2Cl_2 three times (note: an emulsion forms, allow to settle). Wash organic layer with brine. Dry organics over Na_2SO_4 filter and concentrate. Separate crude material on florisil (35g) with a gradient 20–50% EtOAc in Hexanes to provide **17** (540.7 mg, 1.56 mmol, 71% yield) as a white solid

^{1}H NMR (500 MHz, $CDCl_3$) δ 7.74 (d, *J* = 7.6 Hz, 2H), 7.55 – 7.49 (m, 1H), 7.48 – 7.40 (m, 2H), 5.68 (s, 1H), 4.36 (s, 1H), 3.30 (s, 1H), 2.62 (dd, *J* = 13.5, 6.1 Hz, 1H), 2.36 (dt, *J* = 17.3, 5.2 Hz, 1H), 2.22 (dd, *J* = 20.4, 15.0 Hz, 2H), 2.11 (d, *J* = 15.4 Hz, 1H), 1.79 (s, 1H), 1.28 (t, *J* = 7.2 Hz, 3H).

^{13}C NMR (126 MHz, $CDCl_3$) δ 202.53, 170.07, 168.33, 131.70, 128.81, 128.17, 78.81, 62.40, 60.80, 60.30, 55.39, 31.71, 30.86, 22.01, 14.29.

FTIR(AT-IR) 2979.80, 2359.60, 1716.02, 1656.84, 1578.52, 1447.13, 1389.33, 1366.88, 1309.09, 1257.56, 1178.77, 1092.65, 1026.42, 974.69, 920.18, 870.32, 788.06, 748.64, 707.41 cm^{-1}

HRMS (TOF, ES+) calc'd for $C_{18}H_{19}NO_6$ $[M+H]^+$ 346.1285, found 346.1291 (ppm=–1.69)

*Preparation of epoxy enone **17***

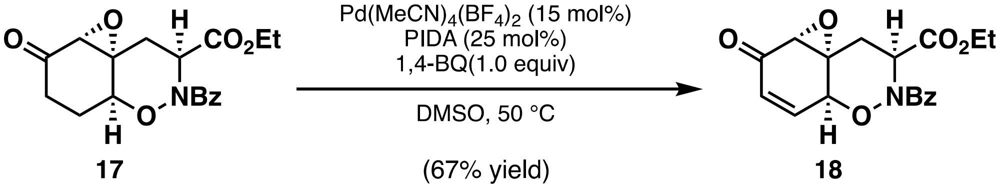

To a solution of substrate **17** (1.688 g, 4.89 mmol, 1.0 equiv) in DMSO(0.15M, 32.5 mL) add 1,4-benzoquinone (660 mg, 6.11 mmol, 1.25 equiv), $Pd(MeCN)_4(BF)_4$ (325.7 mg, 0.733 mmol, 0.15 equiv), and PIDA(394 mg, 1.22 mmol, 0.25 equiv). Heat to 50 °C, stir 96h. Cool to ambient temperature. Add 75 mL $NaHCO_3$(aq), extract 4x175 mL EtOAc. Wash organic layer with 40 mL brine. Dry organic layer with Na_2SO_4, filter, and concentrate. Purify by flash chromatography (silica 175 g, 15%EtOAc/40%CH_2Cl_2/Hexanes) to yield **18** (1.12g, 3.26 mmol, 67% yield).

^{1}H NMR (400 MHz, $CDCl_3$) δ 7.79 – 7.69 (m, 2H), 7.58 – 7.50 (m, 1H), 7.49 – 7.41 (m, 2H), 6.30 (s, 1H), 6.08 (dt, J = 10.6, 1.4 Hz, 1H), 5.65 (s, 1H), 4.72 (s, 1H), 4.34 – 4.12 (m, 3H), 3.53 (t, J = 1.6 Hz, 1H), 2.70 (dd, J = 13.9, 6.2 Hz, 1H), 2.28 (d, J = 14.0 Hz, 1H), 1.27 (t, J = 7.1 Hz, 4H).

^{13}C NMR (101 MHz, $CDCl_3$) δ 192.45, 170.22, 168.04, 136.07, 132.22, 131.90, 130.64, 128.71, 128.27, 76.11, 62.50, 60.42, 59.24, 55.60, 29.61, 14.19.

FTIR (AT-IR) 2982.21, 1737.29, 1690.76, 1661.42, 1600.80, 1579.13, 1447.34, 1390.80, 1365.58, 1334.93, 1306.37, 1266.83, 1226.57, 1187.35, 1026.36, 947.14, 910.44, 859.39, 826.78, 780.45, 729.61, 708.37, 647.95 cm^{-1}

HRMS (TOF, ES+) calc'd for $C_{18}H_{17}NO_6$ $[M+H]^+$ 344.1129, found 344.1127 (ppm=0.48)

Preparation of bis-epoxy ketone ***19***

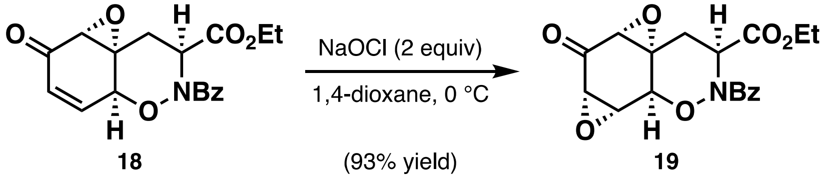

To chill a solution of **18** (1.1207g, 3.26 mmol, 1 equiv) in wet dioxane (13 mL dioxane, 0.1mL DI H_2O) to 0 °C. Add NaOCl (12.5 wt% in H_2O) (3.62mL, 4.37g (243 mg NaOCl), 2.25 equiv) Stir 5h at 0 °C. Dilute

in 1:1 brine/DI H_2O (65 mL). Extract with EtOAc 3x75mL. Dry organics over Na_2SO_4. Filter and concentrate. Take up crude in benzene, concentrate; take up again in hexanes and re-concentrate. Yields **19** (1.09g, 93% yield) as a white foam. [Note: **19** was not amenable to chromatographic purification due to instability and was used with no further purification.]

^{1}H NMR (400 MHz, $CDCl_3$) δ 7.78 – 7.69 (m, 2H), 7.56 – 7.49 (m, 1H), 7.49 – 7.40 (m, 2H), 5.53 (s, 0H), 4.74 (s, 1H), 3.43 (d, J = 1.8 Hz, 1H), 3.40 (s, 1H), 3.39 – 3.33 (m, 1H), 2.63 (dd, J = 14.0, 6.1 Hz, 1H), 2.11 (dd, J = 14.1, 1.8 Hz, 1H), 1.28 (t, J = 7.1 Hz, 4H).

^{13}C NMR (101 MHz, $CDCl_3$) δ 197.03, 170.44, 167.70, 132.10, 131.96, 128.50, 128.32, 75.09, 63.75, 62.60, 61.63, 56.77, 55.37, 54.25, 31.10, 14.14.

FTIR (AT-IR) 1736.36, 1707.34, 1666.01, 1447.25, 1368.08, 1303.87, 1226.04, 1185.33, 1022.17, 947.87, 912.99, 868.45, 788.53, 708.45 cm^{-1}

HRMS (TOF, ES+) calc'd for $C_{18}H_{17}NO_7$ $[M+H]^+$ 360.1078, found 360.1078 (ppm=–0.06)

*Preparation of Wharton products **20a** and **20b***

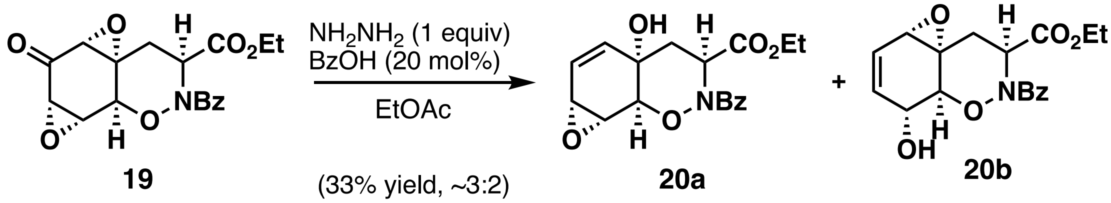

Cool to solution of benzoic acid (6.8 mg, 0.057 mmol, 0.20 equiv) in EtOAc (2mL) to 10 °C. Concurrently, add solutions of NH_2NH_2 (8.9 mg, 8.7μL, 0.278 mmol) in EtOAc (5 mL) and **19** (100 mg, 0.278 mmol) in EtOAc (5 mL) dropwise via syringes to the cooled reaction flask over 30 minutes. Rinse substrate syringe with EtOAc (1 mL). Bring reaction to ambient temperature and stir five minutes before adding triethylamine (1 mL). Filter through a neutralized florisil plug and rinse plug with (5% NEt_3/EtOAc). Purification of that crude mixture by flash chromatography (florisil 5.0g, 5% NEt_3/50%EtOAc/Hexanes) to yield a 3:2 mixture

of **20a** and **20b** (31.4mg, 0.091mmol, 33% yield) which were carried forward as a mixture. **20b** could be isolated cleanly for characterization by flash chromatography (40%EtOAc/Hexanes).

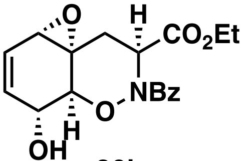

20b

20b: ^{1}H NMR (400 MHz, $CDCl_3$) δ 7.72 (d, J = 7.7 Hz, 2H), 7.56 – 7.47 (m, 1H), 7.46 – 7.36 (m, 2H), 6.19 (dd, J = 9.8, 4.0 Hz, 1H), 6.13 – 6.02 (m, 1H), 5.66 (s, 1H), 4.30 (s, 1H), 4.24 (q, J = 7.1 Hz, 2H), 3.92 (s, 1H), 3.41 (dt, J = 4.1, 1.3 Hz, 1H), 2.69 (dd, J = 13.3, 6.2 Hz, 1H), 2.39 – 2.22 (m, 1H), 1.73 – 1.62 (m, 1H), 1.27 (t, J = 7.1 Hz, 3H).

^{13}C NMR (101 MHz, $CDCl_3$) δ 170.16, 168.79, 136.71, 133.28, 131.70, 128.76, 128.19, 126.98, 81.68, 64.57, 62.28, 59.83, 55.26, 54.45, 31.04, 14.34.

FTIR (AT-IR) 3457.65, 2981.45, 2359.53, 2340.24, 1739.24, 1652.54, 1576.29, 1447.92, 1394.23, 1317.61, 1274.82, 1267.49, 1230.14, 1189.56, 1028.04, 954.06, 867.43, 809.44, 788.73, 763.78, 749.59, 708.28, 667.92 cm^{-1}

HRMS (TOF, ES+) alc'd for $C_{18}H_{19}NO_6$ $[M+H]^+$ 346.1285, found 346.1282 (ppm=0.91)

*Preparation of silylated allylic alcohol **21***

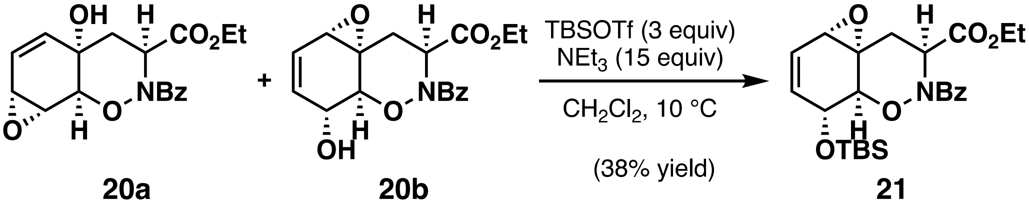

20a **20b** **21**

Cool solution of **20a** and **20b** (31.6 mg, 0.0915 mmol) to –5 °C. Add triethylamine (93mg, 128 μL, 0.915 mmol, 10 equiv) followed by TBSOTf (48.4 mg, 42 μL, 0.183 mmol, 2.0 equiv). Warm to 10 °C and stir 25 minutes. Add additional triethylamine (44 mg, 60 μL, 0.430 mmol, 4.7 equiv) followed by TBSOTf (23 mg, 20 μL, 0.087 mmol, 0.95 equiv). Quench excess TBSOTf with *i*-PrOH (25 μL) and stir at ambient temperature for 5 minutes. Concentrate crude reaction and purify by flash chromatography (florisil 3.6g, 2% NEt_3/5→15%EtOAc/Hexanes) provides **21** (9.6mg, 0.0209 mmol, 38% yield).

^{1}H NMR (400 MHz, $CDCl_3$) δ 7.78 – 7.68 (m, 2H), 7.48 (d, *J* = 1.6 Hz, 1H), 7.44 – 7.38 (m, 2H), 6.04 (ddd, *J* = 10.0, 3.6, 1.3 Hz, 1H), 5.77 (ddd, *J* = 10.0, 4.1, 1.1 Hz, 1H), 5.46 (s, 1H), 4.23 (d, *J* = 5.7 Hz, 2H), 4.16 (s, 1H), 4.08 (d, *J* = 3.8 Hz, 1H), 3.24 (dt, *J* = 3.6, 1.0 Hz, 1H), 2.45 (dd, *J* = 13.8, 5.1 Hz, 2H), 1.29 (t, *J* = 7.1 Hz, 3H), 0.78 (s, 9H), -0.10 (d, *J* = 34.0 Hz, 6H).

^{13}C NMR (101 MHz, $CDCl_3$) δ 169.81, 169.07, 134.70, 132.72, 131.30, 128.61, 128.04, 124.94, 84.87, 66.65, 61.89, 58.77, 52.47, 30.67, 29.69, 25.75, 18.04, 14.19, -4.88, -4.92.

FTIR (AT-IR) 2954.10, 2928.42, 2856.26, 2361.06, 2340.36, 1739.52, 1652.91, 1471.97, 1447.94, 1389.33, 1315.95, 1253.02, 1226.52, 1188.91, 1094.5, 1027.62, 915.93, 878.84, 837.35, 814.81, 778.37, 746.41, 706.37, 668.03, 654.57, 648.90, 632.41, 617.5, 608.61 cm^{-1}

HRMS (TOF, ES+) calc'd for $C_{24}H_{33}NO_6Si$ $[M+H]^+$ 460.2150, found 460.2146 (ppm=0.85)

Preparation of N-H THO derivative ***22***

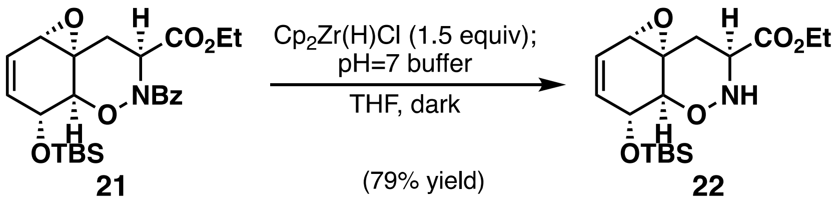

To a stirred suspension of $Cp_2Zr(H)Cl$ (12.4 mg, 0.0320 mmol, 1.5 equiv) in THF (0.15 mL) add **21** (14.7 mg, 0.0320 mmol, 1.0 equiv) in a steady stream as a solution in THF (1.75 mL). Rinse substrate syringe with THF (3×0.15mL) Stir at ambient temperature for 10 minutes. Quench reaction with the rapid addition of a pH=7 phosphate buffer (0.50 mL). Extract aqueous four times with EtOAc. Dry organics over Na_2SO_4. Filter and concentrate, purify crude product by flash chromatography (florisil 1.50 g, 10–60% EtOAc/Hexanes, +10% EtOAc/10 mL eluent). Concentrate and re–concentrate from dry toluene to yield **22** as a white solid (9.0 mg, 0.0253 mmol, 79% yield).

^{1}H NMR (400 MHz, $CDCl_3$) δ 6.41 (s, 1H), 6.06 (d, J = 3.8 Hz, 1H), 5.82 (dddd, J = 10.0, 5.0, 1.3, 0.7 Hz, 1H), 4.34 – 4.16 (m, 2H), 4.15 (dt, J = 1.7, 0.8 Hz, 1H), 4.13 (ddd, J = 5.0, 1.9, 0.8 Hz, 1H), 3.90 (ddd, J =

6.0, 2.8, 1.0 Hz, 1H), 3.21 (dt, J = 4.0, 1.2 Hz, 1H), 2.57 (dd, J = 13.4, 5.9 Hz, 1H), 2.12 (dt, J = 13.4, 2.3 Hz, 1H), 1.29 (t, J = 7.1 Hz, 3H), 0.89 (s, 9H), 0.09 (d, J = 8.4 Hz, 6H).

^{13}C NMR (126 MHz, $CDCl_3$) δ 171.70, 133.48, 125.45, 81.96, 67.70, 61.78, 59.88, 59.68, 53.79, 31.59, 26.08, 18.43, 14.41, -4.34.

FTIR (AT-IR) 2928.00, 2855.59, 2361.23, 2339.00, 1734.81, 1472.07, 1462.93, 1388.10, 1251.36, 1225.94, 1180.11, 1082.99, 1026.99, 1005.17, 931.05, 859.36, 836.96, 776.80, 739.28, 667.95 cm^{-1}

HRMS (TOF, ES+) calc'd for $C_{17}H_{29}NO_5Si$ $[M+H]^+$ 356.1888, found 356.1891 (ppm=-0.91)

*Derivatization of **18** for X-ray crystallography*

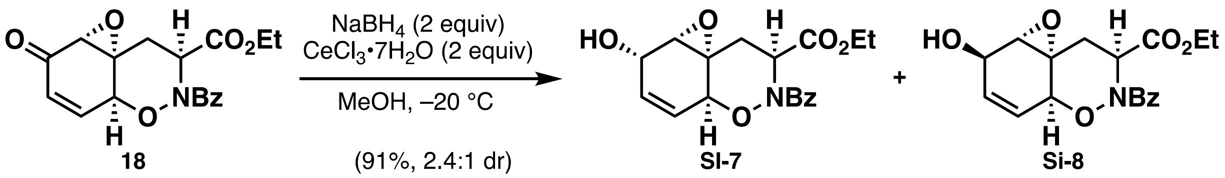

Cool **18** (140 mg, 0.408mg, 1.0 equiv) and $CeCl_3$•$7H_2O$ (304mg, 0.816 mmol, 2.0 equiv) in MeOH (6.7 mL) to –20 °C. Add a solution of $NaBH_4$ (30.9 mg, 0.816 mmol, 2.0 equiv) and stir for 30 minutes before raising the temperature to 0 °C. Add $NaHCO_{3(aq)}$ (9 mL). Extract with EtOAc four times. Wash combined organics with brine. Dry over Na_2SO_4, filter, and concentrate. Purification by flash chromatography (fine silica, 20% PhMe/40% Acetone/Hexanes) provided **SI-7** (63.3 mg, 0.183 mmol, 45% yield) and some mixed fractions which were subsequently purified on normal phase prep-HPLC (45% EtOAc/Hexanes) to provide additional **SI-7** (27.0mg, 0.078 mmol, 19% yield) and **SI-8** (38.4 mg, 0.111 mmol, 27% yield).

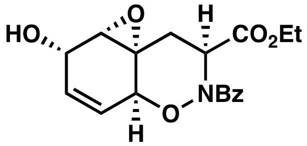

377a

SI-7: ^{1}H NMR (500 MHz, $CDCl_3$) δ 7.74 (d, *J* = 7.6 Hz, 2H), 7.54 – 7.48 (m, 1H), 7.46 – 7.39 (m, 2H), 5.79 (d, *J* = 10.3 Hz, 1H), 5.66 (s, 1H), 5.38 (s, 1H), 4.47 (s, 1H), 4.40 (s, 1H), 4.32 – 4.18 (m, 2H), 3.59 (t, *J* = 2.0 Hz, 1H), 2.66 – 2.53 (m, 1H), 2.24 (d, *J* = 13.7 Hz, 1H), 2.08 (s, 1H), 1.64 (s, 1H), 1.29 (t, *J* = 7.1 Hz, 3H).

^{13}C NMR (126 MHz, $CDCl_3$) δ 170.09, 168.74, 133.50, 131.72, 128.88, 128.16, 119.84, 76.90, 65.00, 62.29, 62.01, 56.89, 55.35, 30.65, 14.31.

FTIR (AT-IR) 3515.22, 2359.36, 2340.32, 1713.29, 1651.27, 1448.14, 1400.82, 1371.84, 1300.75, 1271.34, 1237.79, 1198.4, 1086.26, 1044.77, 1015.91, 974.42, 917.96, 850.56, 808.77, 791.48, 707.66, 668.00, 627.70 cm^{-1}

HRMS (TOF, ES+) calc'd for $C_{18}H_{19}NO_6$ $[M+H]^+$ 346.1285, found 346.1281 (ppm=1.20)

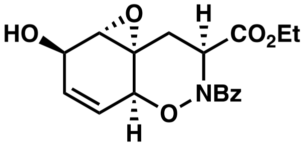
377b

SI-8: **^{1}H NMR** (500 MHz, $CDCl_3$) δ 7.74 (d, *J* = 7.6 Hz, 2H), 7.55 – 7.47 (m, 1H), 7.46 – 7.40 (m, 2H), 5.96 (ddd, *J* = 9.6, 4.6, 2.0 Hz, 1H), 5.55 (d, *J* = 74.0 Hz, 2H), 4.50 (s, 1H), 4.41 (s, 1H), 4.34 – 4.19 (m, 2H), 3.37 (q, *J* = 1.6 Hz, 1H), 2.60 (dd, *J* = 13.6, 6.1 Hz, 1H), 2.16 (dd, *J* = 13.7, 1.9 Hz, 1H), 1.93 (s, 1H), 1.31 (td, *J* = 7.1, 0.9 Hz, 3H).

^{13}C NMR (126 MHz, $CDCl_3$) δ 170.24, 168.80, 131.97, 131.75, 128.90, 128.18, 120.92, 76.79, 63.26, 62.39, 60.50, 55.64, 53.93, 30.96, 14.32.

FTIR (AT-IR) 3506.46, 2925.60, 2359.53, 2340.3, 1733.94, 1653.54, 1578.26, 1447.05, 1367.84, 1298.39, 1233.21, 1189.33, 1025.82, 905.79, 867.34, 831.29, 790.72, 768.16, 730.21, 706.66, 667.91 cm^{-1}

HRMS (TOF, ES+) calc'd for $C_{18}H_{19}NO_6$ $[M+H]^+$ 346.1285, found 346.1275 (ppm=1.20)

3. Single Crystal X-ray Diffraction Data

Determination of enantiomeric series – desilylated S-hydroxylamine (p16469_b)

Crystal was obtained in analogy to **14** with 2-Br-benzoyl chloride. Material was recrystallized from $CHCl_3$/hexanes. Layer diffusion between 1:1 $CHCl_3$/Hexanes and Hexanes yielded X-ray quality crystals of a desilylated hydroxylamine.

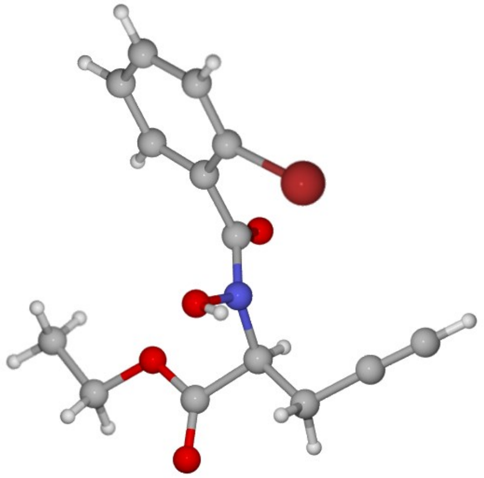

Table 1. Crystal data and structure refinement for final p16469_b.

Identification code	p16469_b	
Empirical formula	C14 H14 Br N O4	
Formula weight	340.17	
Temperature	100(2) K	
Wavelength	0.71073 Å	
Crystal system	Monoclinic	
Space group	$P12_11$	
Unit cell dimensions	a = 8.6346(7) Å	α = 90°.
	b = 5.6380(5) Å	β = 103.728(3)°
	c = 15.6510(13) Å	γ = 90°
Volume	740.15(11) Å^3	
Z	2	
Density (calculated)	1.410 Mg/m^3	
Absorption coefficient 0	2.788 mm^{-1}	
F(000)	344	
Crystal size	0.23 × 0.15 × 0.07 mm^3	
Theta range for data collection	2.428 to 45.369°	
Index ranges	-16<=h<=17, -11<=k<=11, -31<=l<=31	
Reflections collected	68030	
Independent reflections	12256 [R(int) = 0.0378]	
Completeness to theta = 25.000°	99.7 %	
Absorption correction	Semi-empirical from equivalents	
Max. and min. transmission	0.747 and 0.716	
Refinement method	Full-matrix least-squares on F2	
Data / restraints / parameters	12256/1/183	
Goodness-of-fit on F2	0.989	
Final R indices [I>2sigma(I)]	R1 = 0.0241, wR2 = 0.0526	
R indices (all data)	R1 = 0.0305, wR2 = 0.0541	
Extinction coefficient	n/a	
Largest diff. peak and hole	0.526 and -0.838 e.Å^{-3}	

Table 2. Atomic coordinates (×10^4) and equivalent isotropic displacement parameters (Å 2×10^3) for final p16469_b. U(eq) is defined as one third of the trace of the orthogonalized U^{ij} tensor.

	**x**	**y**	**z**	**U_{eq}**
Br1	0.15673(2)	0.20544(3)	0.66433(2)	0.02070(3)
O1	0.86808(9)	0.26712(16)	0.92479(6)	0.01991(14)

O2	0.80569(9)	0.56641(15)	0.82777(6)	0.01865(13)
O3	0.54192(7)	0.19604(14)	0.73103(4)	0.01256(9)
O4	0.492	0.0738	0.7396	0.019
N1	0.43360(9)	0.76829(12)	0.77810(5)	0.01514(11)
C1	0.50418(9)	0.38290(13)	0.78093(5)	0.00968(10)
C2	0.77148(10)	0.39468(16)	0.87841(6)	0.01261(12)
C3	0.59223(10)	0.38553(15)	0.87231(5)	0.00991(11)
C4	0.5623	0.5331	0.9	0.012
C5	0.55204(9)	0.17365(15)	0.92407(5)	0.01207(13)
C6	0.5754	0.025	0.8959	0.014
C7	0.6205	0.1778	0.9845	0.014
C8	0.38480(10)	0.17397(16)	0.92825(6)	0.01385(14)
C9	0.24845(11)	0.1758(2)	0.93395(7)	0.01919(18)
C10	0.1407	0.1773	0.9384	0.023
C11	0.43888(10)	0.58063(14)	0.73832(5)	0.00977(11)
C12	0.37367(10)	0.56879(15)	0.64074(5)	0.01081(11)
C13	0.25012(11)	0.42021(17)	0.59858(6)	0.01398(13)
C14	0.18903(13)	0.4296(2)	0.50811(7)	0.01944(17)

Table 3. Bond lengths [Å] and angles [°] for final p16469_b

Br1–C8	1.8892(9)
O1–C1	1.2052(12)
O2–C1	1.3282(11)
O2–C13	1.4559(13)
O3–H3	0.8400
O3–N1	1.3953(10)
O4–C6	1.2340(11)
N1–C2	1.4518(11)
N1–C6	1.3517(11)
C1–C2	1.5288(12)
C2–H2	1.0000
C2–C3	1.5287(11)
C3–H3A	0.9900
C3–H3B	0.9900
C3–C4	1.4610(11)
C4–C5	1.2017(12)
C5–H5	0.9500
C6–C7	1.4986(11)
C7–C8	1.3932(13)
C7–C12	1.3987(12)
C8–C9	1.3899(14)
C9–H9	0.9500
C9–C10	1.3905(15)
C10–H10	0.9500
C10–C11	1.3858(16)
C11–H11	0.9500
C11–C12	1.3931(13)
C12–H12	0.9500
C13–H13A	0.9900
C13–H13B	0.9900
C13–C14	1.5054(17)
C14–H14A	0.9800
C14–H14B	0.9800
C14–H14C	0.9800

C1–O2–C13	115.36(8)

N1–O3–H3	109.5
O3–N1–C2	114.95(6)
C6–N1–O3	118.17(7)
C6–N1–C2	122.50(7)
O1–C1–O2	125.05(9)
O1–C1–C2	124.28(8)
O2–C1–C2	110.65(8)
N1–C2–C1	110.34(7)
N1–C2–H2	107.8
N1–C2–C3	112.48(7)
C1–C2–H2	107.8
C3–C2–C1	110.31(7)
C3–C2–H2	107.8
C2–C3–H3A	109.2
C2–C3–H3B	109.2
H3A–C3–H3B	107.9
C4–C3–C2	111.95(7)
C4–C3–H3A	109.2
C4–C3–H3B	109.2
C5–C4–C3	178.30(10)
C4–C5–H5	180.0
O4–C6–N1	121.28(8)
O4–C6–C7	120.17(8)
N1–C6–C7	118.55(7)
C8–C7–C6	124.73(7)
C8–C7–C12	118.27(8)
C12–C7–C6	116.78(8)
C7–C8–Br1	120.35(6)
C9–C8–Br1	118.14(7)
C9–C8–C7	121.49(9)
C8–C9–H9	120.4
C8–C9–C10	119.29(10)
C10–C9–H9	120.4
C9–C10–H10	119.8
C11–C10–C9	120.33(9)
C11–C10–H10	119.8
C10–C11–H11	120.1
C10–C11–C12	119.88(9)
C12–C11–H11	120.1
C7–C12–H12	119.6
C11–C12–C7	120.70(9)
C11–C12–H12	119.6
O2–C13–H13A	110.3
O2–C13–H13B	110.3
O2–C13–C14	107.04(9)
H13A–C13–H13B	108.6
C14–C13–H13A	110.3
C14–C13–H13B	110.3
C13–C14–H14A	109.5
C13–C14–H14B	109.5
C13–C14–H14C	109.5
H14A–C14–H14B	109.5
H14A–C14–H14C	109.5

Symmetry transformations used to generate equivalent atoms:

Table 4. Anisotropic displacement parameters (Å^2) for final p16469_b. The anisotropic displacement factor exponent takes the form: -2p^2[h^2 a*^{2}U^{11} + ... + 2 h k a* b*U^{12}]

	U^{11}	U^{22}	U^{33}	U^{23}	U^{13}	U^{12}
Br1	0.01666(4)	0.02574(5)	0.01784(4)	0.00477(4)	0.00036(3)	-0.01092(4)

O1	0.0128(2)	0.0248(4)	0.0218(3)	0.0102(3)	0.0035(2)	0.0027(2)
O2	0.0128(2)	0.0203(3)	0.0226(3)	0.0095(3)	0.0035(2)	-0.0029(2)
O3	0.0179(2)	0.0080(2)	0.0122(2)	-0.0020(2)	0.00421(17)	0.0012(2)
O4	0.0234(3)	0.0083(2)	0.0131(2)	-0.00124(19)	0.0030(2)	0.0014(2)
N1	0.0128(2)	0.0076(2)	0.0080(2)	-0.00085(18)	0.00111(19)	0.00054(19)
C1	0.0118(3)	0.0137(3)	0.0119(3)	0.0014(2)	0.0019(2)	-0.0021(2)
C2	0.0109(3)	0.0099(3)	0.0084(3)	0.0002(2)	0.0013(2)	-0.0013(2)
C3	0.0123(3)	0.0127(4)	0.0108(3)	0.0025(2)	0.0019(2)	-0.0012(2)
C4	0.0139(3)	0.0153(4)	0.0119(3)	0.0031(2)	0.0024(2)	-0.0019(2)
C5	0.0148(3)	0.0245(5)	0.0180(3)	0.0066(3)	0.0035(3)	-0.0002(3)
C6	0.0115(3)	0.0083(3)	0.0095(3)	0.0004(2)	0.0023(2)	0.0000(2)
C7	0.0129(3)	0.0101(3)	0.0092(3)	0.0010(2)	0.0023(2)	-0.0001(2)
C8	0.0126(3)	0.0166(3)	0.0114(3)	0.0020(3)	0.0003(2)	-0.0022(3)
C9	0.0164(4)	0.0268(5)	0.0123(3)	0.0014(3)	-0.0022(3)	-0.0031(3)
C10	0.0211(4)	0.0267(5)	0.0104(3)	0.0041(3)	0.0004(3)	0.0014(3)
C11	0.0262(4)	0.0187(5)	0.0120(3)	0.0044(3)	0.0058(3)	-0.0009(3)
C12	0.0216(3)	0.0122(4)	0.0122(3)	0.0016(2)	0.0048(2)	-0.0025(3)
C13	0.0139(4)	0.0264(5)	0.0263(5)	0.0092(4)	0.0048(3)	-0.0038(3)
C14	0.0238(5)	0.0323(6)	0.0394(7)	0.0178(5)	0.0113(5)	-0.0040(4)

Conformational preferences of bicyclic tetrahydro-1,2–oxazine – epoxy allylic alcohol (a16027_a)

X-ray quality crystals of **SI-8** obtained layer diffusion between 1:1 $CHCl_3$/hexanes and hexanes.

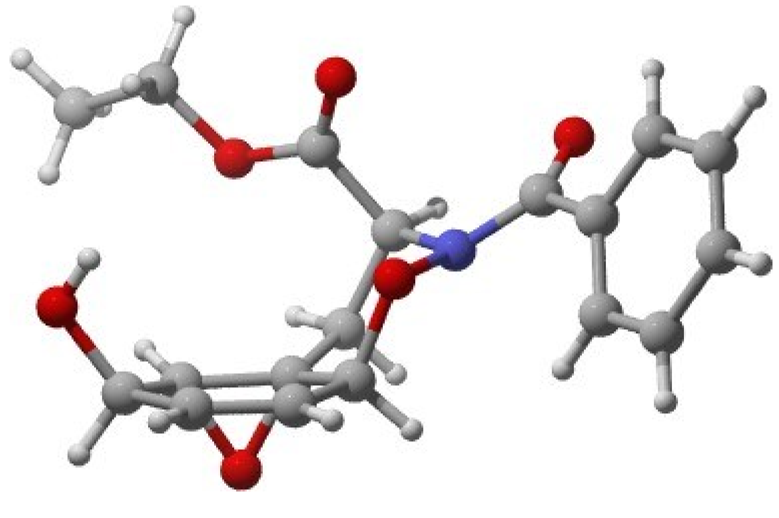

Table 1. Crystal data and structure refinement for a16027_a.cif.

Identification code	a16027_a	
Empirical formula	C18 H19 N O6	
Formula weight	345.34	
Temperature	99.99 K	
Wavelength	0.71073 Å	
Crystal system	Triclinic	
Space group	P-1	
Unit cell dimensions	a = 9.4539(6) Å	□= 114.437(3)°.
	b = 9.8703(7) Å	□= 105.326(3)°.
	c = 10.6887(7) Å	□ = 102.469(3)°.

Volume	813.45(10) Å^3
Z	2
Density (calculated)	1.410 Mg/m^3
Absorption coefficient	0.107 mm^{-1}
F(000)	364
Crystal size	0.8 x 0.45 x 0.35 mm3
Theta range for data collection	2.281 to 37.744°.
Index ranges	-15<=h<=16, -16<=k<=16, -18<=l<=18
Reflections collected	65383
Independent reflections	8412 [R(int) = 0.0252]
Completeness to theta = 26.000°	99.9 %
Absorption correction	Semi-empirical from equivalents
Max. and min. transmissio n	0.7474 and 0.7156
Refinement method	Full-matrix least-squares on F2
Data / restraints / parameters	8412 / 0 / 228
Goodness-of-fit on F2	1.043
Final R indices [I>2sigma(I)]	R1 = 0.0343, wR2 = 0.1005
R indices (all data)	R1 = 0.0395, wR2 = 0.1046
Extinction coefficient	n/a
Largest diff. peak and hole	0.555 and -0.205 e.Å^{-3}

Table 2. Atomic coordinates (×10^4) and equivalent isotropic displacement parameters (Å^2× 10^3) for a16027_a. $U_{(eq)}$ is defined as one third of the trace of the orthogonalized U_{ij} tensor.

	x	y	z	U(eq)
O(1)	6873(1)	5237(1)	993(1)	23(1)
O(2)	4078(1)	7342(1)	4849(1)	21(1)
O(3)	1513(1)	4450(1)	837(1)	21(1)
O(4)	6198(1)	8195(1)	2623(1)	12(1)
O(5)	10068(1)	8470(1)	3357(1)	20(1)
O(6)	5780(1)	4128(1)	2137(1)	17(1)
N(1)	7672(1)	8243(1)	3435(1)	13(1)
C(1)	6678(1)	5329(1)	2092(1)	14(1)
C(2)	7469(1)	6856(1)	3630(1)	14(1)
C(3)	6542(1)	6982(1)	4627(1)	17(1)
C(4)	5059(1)	7241(1)	4009(1)	14(1)
C(5)	3483(1)	6000(1)	3339(1)	17(1)
C(6)	2082(1)	5930(1)	2205(1)	18(1)
C(7)	2411(1)	7354(1)	1984(1)	18(1)
C(8)	3827(1)	8515(1)	2614(1)	17(1)
C(9)	5306(1)	8516(1)	3564(1)	14(1)
C(10)	8946(1)	8903(1)	3200(1)	14(1)
C(11)	8956(1)	10245(1)	2885(1)	15(1)
C(12)	9779(1)	10449(1)	2025(1)	20(1)
C(13)	9905(1)	11734(1)	1767(1)	26(1)
C(14)	9229(1)	12823(1)	2380(1)	27(1)
C(15)	8441(1)	12645(1)	3270(1)	23(1)
C(16)	8295(1)	11352(1)	3517(1)	17(1)
C(17)	4949(1)	2613(1)	725(1)	22(1)
C(18)	4116(1)	1415(1)	1064(1)	27(1)

Table 3. Bond lengths [Å] and angles [°] for a16027_a

O(1)-C(1)	1.2069(7)
O(2)-C(4)	1.4424(7)

O(2)-C(5)	1.4510(7)
O(3)-H(3)	0.8400
O(3)-C(6)	1.4277(8)
O(4)-N(1)	1.4127(6)
O(4)-C(9)	1.4561(6)
O(5)-C(10)	1.2234(7)
O(6)-C(1)	1.3270(7)
O(6)-C(17)	1.4591(7)
N(1)-C(2)	1.4480(7)
N(1)-C(10)	1.3767(7)
C(1)-C(2)	1.5316(7)
C(2)-H(2)	1.0000
C(3)-H(3A)	0.9900
C(3)-H(3B)	0.9900
C(3)-C(4)	1.5075(8)
C(4)-C(5)	1.4682(8)
C(4)-C(9)	1.5140(8)
C(5)-H(5)	1.0000
C(5)-C(6)	1.5068(9)
C(6)-H(6)	1.0000
C(6)-C(7)	1.5011(8)
C(7)-H(7)	0.9500
C(7)-C(8)	1.3347(8)
C(8)-H(8)	0.9500
C(8)-C(9)	1.4944(8)
C(9)-H(9)	1.0000
C(10)-C(11)	1.4945(8)
C(11)-C(12)	1.3977(8)
C(11)-C(16)	1.3956(8)
C(12)-H(12)	0.9500
C(12)-C(13)	1.3932(10)
C(13)-H(13)	0.9500
C(13)-C(14)	1.3882(12)
C(14)-H(14)	0.9500
C(14)-C(15)	1.3950(10)
C(15)-H(15)	0.9500
C(15)-C(16)	1.3928(8)
C(16)-H(16)	0.9500
C(17)-H(17A)	0.9900
C(17)-H(17B)	0.9900
C(17)-C(18)	1.5017(10)
C(18)-H(18A)	0.9800
C(18)-H(18B)	0.9800
C(18)-H(18C)	0.9800
C(4)-O(2)-C(5)	60.99(3)
C(6)-O(3)-H(3)	109.5
N(1)-O(4)-C(9)	109.45(4)
C(1)-O(6)-C(17)	116.14(5)
O(4)-N(1)-C(2)	110.53(4)
C(10)-N(1)-O(4)	116.60(4)
C(10)-N(1)-C(2)	122.41(4)
O(1)-C(1)-O(6)	124.83(5)
O(1)-C(1)-C(2)	123.79(5)
O(6)-C(1)-C(2)	111.38(4)
N(1)-C(2)-C(1)	109.45(4)
N(1)-C(2)-H(2)	108.5
N(1)-C(2)-C(3)	107.27(4)
C(1)-C(2)-H(2)	108.5
C(1)-C(2)-C(3)	114.37(4)
C(3)-C(2)-H(2)	108.5

C(2)-C(3)-H(3A)	109.6
C(2)-C(3)-H(3B)	109.6
H(3A)-C(3)-H(3B)	108.1
C(4)-C(3)-C(2)	110.42(4)
C(4)-C(3)-H(3A)	109.6
C(4)-C(3)-H(3B)	109.6
O(2)-C(4)-C(5)	59.80(4)
O(2)-C(4)-C(9)	114.11(5)
C(3)-C(4)-C(9)	114.18(4)
C(5)-C(4)-C(3)	122.07(5)
C(5)-C(4)-C(9)	119.51(5)
O(2)-C(5)-C(4)	59.22(3)
O(2)-C(5)-H(5)	115.7
O(2)-C(5)-C(6)	115.76(5)
C(4)-C(5)-H(5)	115.7
C(4)-C(5)-C(6)	122.52(5)
C(6)-C(5)-H(5)	115.7
O(3)-C(6)-C(5)	109.05(5)
O(3)-C(6)-H(6)	107.2
O(3)-C(6)-C(7)	112.48(5)
C(5)-C(6)-H(6)	107.2
C(7)-C(6)-C(5)	113.46(5)
C(7)-C(6)-H(6)	107.2
C(6)-C(7)-H(7)	117.8
C(8)-C(7)-C(6)	124.41(5)
C(8)-C(7)-H(7)	117.8
C(7)-C(8)-H(8)	117.9
C(7)-C(8)-C(9)	124.22(5)
C(9)-C(8)-H(8)	117.9
O(4)-C(9)-C(4)	108.30(4)
O(4)-C(9)-C(8)	104.16(4)
O(4)-C(9)-H(9)	109.8
C(4)-C(9)-H(9)	109.8
C(8)-C(9)-C(4)	114.81(4)
C(8)-C(9)-H(9)	109.8
O(5)-C(10)-N(1)	120.20(5)
O(5)-C(10)-C(11)	122.11(5)
N(1)-C(10)-C(11)	117.49(4)
C(12)-C(11)-C(10)	117.52(5)
C(16)-C(11)-C(10)	122.35(5)
C(16)-C(11)-C(12)	119.92(5)
C(11)-C(12)-H(12)	120.0
C(13)-C(12)-C(11)	120.05(6)
C(13)-C(12)-H(12)	120.0
C(12)-C(13)-H(13)	120.0
C(14)-C(13)-C(12)	119.93(6)
C(14)-C(13)-H(13)	120.0
C(13)-C(14)-H(14)	119.9
C(13)-C(14)-C(15)	120.18(6)
C(15)-C(14)-H(14)	119.9
C(14)-C(15)-H(15)	119.9
C(16)-C(15)-C(14)	120.11(6)
C(16)-C(15)-H(15)	119.9
C(11)-C(16)-H(16)	120.1
C(15)-C(16)-C(11)	119.79(5)
C(15)-C(16)-H(16)	120.1
O(6)-C(17)-H(17A)	110.4
O(6)-C(17)-H(17B)	110.4
O(6)-C(17)-C(18)	106.77(5)
H(17A)-C(17)-H(17B)	108.6
C(18)-C(17)-H(17A)	110.4

C(18)-C(17)-H(17B)	110.4
C(17)-C(18)-H(18A)	109.5
C(17)-C(18)-H(18B)	109.5
C(17)-C(18)-H(18C)	109.5
H(18A)-C(18)-H(18B)	109.5
H(18A)-C(18)-H(18C)	109.5
H(18B)-C(18)-H(18C)	109.5

Symmetry transformations used to generate equivalent atoms:

Table 4. Anisotropic displacement parameters (Å^{2}x 10^3) for a16027_a. The anisotropic displacement factor exponent takes the form: -2p2[h2 a*2U11 + ... + 2 h k a* b* U12]

	U^{11}	U^{22}	U^{33}	U^{23}	U^{13}	U^{12}
O(1)	34(1)	20(1)	18(1)	9(1)	16(1)	9(1)
O(2)	23(1)	22(1)	17(1)	6(1)	14(1)	4(1)
O(3)	19(1)	17(1)	21(1)	8(1)	7(1)	0(1)
O(4)	9(1)	14(1)	14(1)	6(1)	6(1)	5(1)
O(5)	15(1)	29(1)	24(1)	15(1)	10(1)	13(1)
O(6)	23(1)	12(1)	15(1)	7(1)	6(1)	5(1)
N(1)	10(1)	14(1)	16(1)	8(1)	5(1)	4(1)
C(1)	18(1)	13(1)	15(1)	8(1)	7(1)	8(1)
C(2)	14(1)	15(1)	13(1)	7(1)	5(1)	5(1)
C(3)	18(1)	19(1)	11(1)	7(1)	5(1)	5(1)
C(4)	16(1)	14(1)	12(1)	5(1)	8(1)	4(1)
C(5)	18(1)	15(1)	16(1)	8(1)	10(1)	3(1)
C(6)	14(1)	16(1)	22(1)	9(1)	10(1)	3(1)
C(7)	13(1)	18(1)	27(1)	12(1)	11(1)	7(1)
C(8)	14(1)	14(1)	26(1)	11(1)	11(1)	7(1)
C(9)	13(1)	12(1)	16(1)	5(1)	9(1)	4(1)
C(10)	11(1)	17(1)	13(1)	7(1)	6(1)	5(1)
C(11)	10(1)	18(1)	15(1)	9(1)	5(1)	3(1)
C(12)	14(1)	28(1)	20(1)	13(1)	8(1)	4(1)
C(13)	17(1)	34(1)	27(1)	21(1)	8(1)	2(1)
C(14)	19(1)	27(1)	34(1)	23(1)	6(1)	1(1)
C(15)	17(1)	19(1)	31(1)	16(1)	7(1)	4(1)
C(16)	13(1)	17(1)	21(1)	10(1)	7(1)	4(1)
C(17)	30(1)	13(1)	17(1)	5(1)	6(1)	6(1)
C(18)	23(1)	18(1)	28(1)	11(1)	3(1)	0(1)

4.

Table 5. Hydrogen coordinates (x 10^4) and isotropic displacement parameters (Å^{2}x 10^3) for a16027_a.

	x	y	z	U(eq)
H(3)	2000	4558	310	31
H(2)	8535	6899	4164	17
H(3A)	6262	5987	4680	20
H(3B)	7208	7887	5655	20
H(5)	3460	4949	3261	20
H(6)	1229	5920	2593	21

H(7)	1553	7434	1352	22

H(8)	3900	9397	2448	20
H(9)	5908	9597	4480	17
H(12)	10252	9711	1616	24
H(13)	10453	11866	1172	31
H(14)	9304	13692	2194	32
H(15)	8003	13407	3708	27
H(16)	7748	11224	4113	20
H(17A)	5707	2261	308	27
H(17B)	4178	2734	-14	27
H(18A)	3545	373	142	40
H(18B)	3366	1775	1470	40
H(18C)	4892	1313	1801	40

Table 6. Torsion angles [°] for a16027_a.

O(1)-C(1)-C(2)-N(1)	32.43(7)
O(1)-C(1)-C(2)-C(3)	152.80(6)
O(2)-C(4)-C(5)-C(6)	102.72(6)
O(2)-C(4)-C(9)-O(4)	-175.31(4)
O(2)-C(4)-C(9)-C(8)	-59.41(6)
O(2)-C(5)-C(6)-O(3)	-172.59(4)
O(2)-C(5)-C(6)-C(7)	61.20(6)
O(3)-C(6)-C(7)-C(8)	-118.76(6)
O(4)-N(1)-C(2)-C(1)	59.09(5)
O(4)-N(1)-C(2)-C(3)	-65.52(5)
O(4)-N(1)-C(10)-O(5)	-151.78(5)
O(4)-N(1)-C(10)-C(11)	33.40(6)
O(5)-C(10)-C(11)-C(12)	33.04(8)
O(5)-C(10)-C(11)-C(16)	-141.58(6)
O(6)-C(1)-C(2)-N(1)	-148.56(4)
O(6)-C(1)-C(2)-C(3)	-28.19(6)
N(1)-O(4)-C(9)-C(4)	-60.15(5)
N(1)-O(4)-C(9)-C(8)	177.21(4)
N(1)-C(2)-C(3)-C(4)	52.05(5)
N(1)-C(10)-C(11)-C(12)	-152.25(5)
N(1)-C(10)-C(11)-C(16)	33.14(7)
C(1)-O(6)-C(17)-C(18)	175.96(5)
C(1)-C(2)-C(3)-C(4)	-69.52(6)
C(2)-N(1)-C(10)-O(5)	-10.28(8)
C(2)-N(1)-C(10)-C(11)	174.90(4)
C(2)-C(3)-C(4)-O(2)	178.71(4)
C(2)-C(3)-C(4)-C(5)	110.02(5)
C(2)-C(3)-C(4)-C(9)	-46.69(6)
C(3)-C(4)-C(5)-O(2)	102.16(5)
C(3)-C(4)-C(5)-C(6)	-155.13(5)
C(3)-C(4)-C(9)-O(4)	49.71(6)
C(3)-C(4)-C(9)-C(8)	165.61(4)
C(4)-O(2)-C(5)-C(6)	-114.03(5)
C(4)-C(5)-C(6)-O(3)	118.90(5)
C(4)-C(5)-C(6)-C(7)	-7.31(8)
C(5)-O(2)-C(4)-C(3)	-114.02(5)
C(5)-O(2)-C(4)-C(9)	111.34(5)
C(5)-C(4)-C(9)-O(4)	-107.64(5)
C(5)-C(4)-C(9)-C(8)	8.26(7)
C(5)-C(6)-C(7)-C(8)	5.62(8)

C(6)-C(7)-C(8)-C(9)	3.49(9)
C(7)-C(8)-C(9)-O(4)	107.72(6)
C(7)-C(8)-C(9)-C(4)	-10.54(8)
C(9)-O(4)-N(1)-C(2)	71.68(5)
C(9)-O(4)-N(1)-C(10)	-142.45(4)
C(9)-C(4)-C(5)-O(2)	-102.33(5)
C(9)-C(4)-C(5)-C(6)	0.39(8)
C(10)-N(1)-C(2)-C(1)	-84.44(6)
C(10)-N(1)-C(2)-C(3)	150.94(5)
C(10)-C(11)-C(12)-C(13)	-176.34(5)
C(10)-C(11)-C(16)-C(15)	175.33(5)
C(11)-C(12)-C(13)-C(14)	0.77(9)
C(12)-C(11)-C(16)-C(15)	0.83(8)
C(12)-C(13)-C(14)-C(15)	0.80(10)
C(13)-C(14)-C(15)-C(16)	-1.55(10)
C(14)-C(15)-C(16)-C(11)	0.73(9)
C(16)-C(11)-C(12)-C(13)	-1.59(8)
C(17)-O(6)-C(1)-O(1)	-1.75(8)
C(17)-O(6)-C(1)-C(2)	179.25(5)

Symmetry transformations used to generate equivalent atoms:

4. ^{1}H and ^{13}C NMR Spectral Data

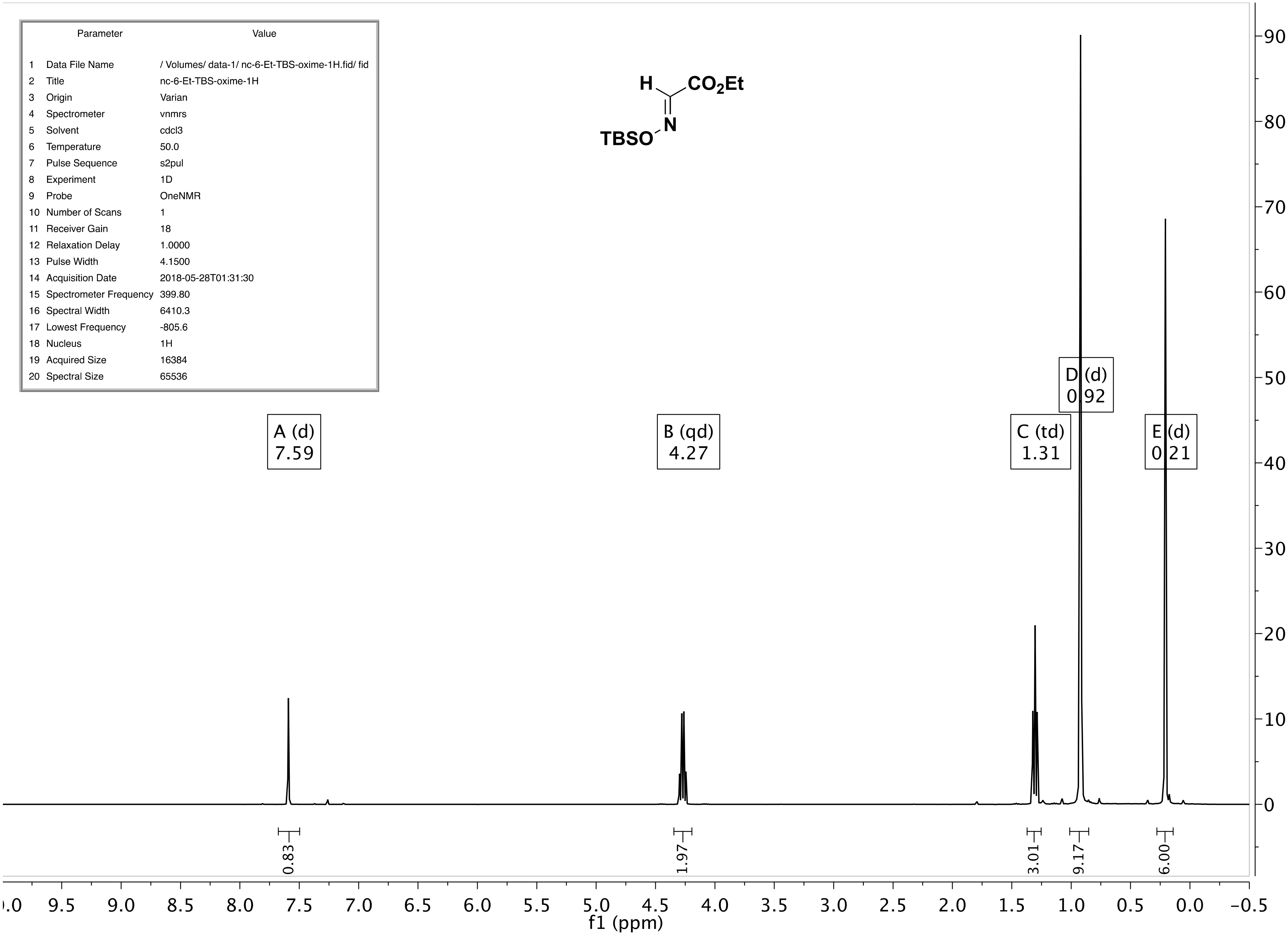

	Parameter	Value
1	Data File Name	/ Volumes/ data-1/ nc-6-Et-TBS-oxime-1H.fid/ fid
2	Title	nc-6-Et-TBS-oxime-1H
3	Origin	Varian
4	Spectrometer	vnmrs
5	Solvent	cdcl3
6	Temperature	50.0
7	Pulse Sequence	s2pul
8	Experiment	1D
9	Probe	OneNMR
10	Number of Scans	1
11	Receiver Gain	18
12	Relaxation Delay	1.0000
13	Pulse Width	4.1500
14	Acquisition Date	2018-05-28T01:31:30
15	Spectrometer Frequency	399.80
16	Spectral Width	6410.3
17	Lowest Frequency	-805.6
18	Nucleus	1H
19	Acquired Size	16384
20	Spectral Size	65536

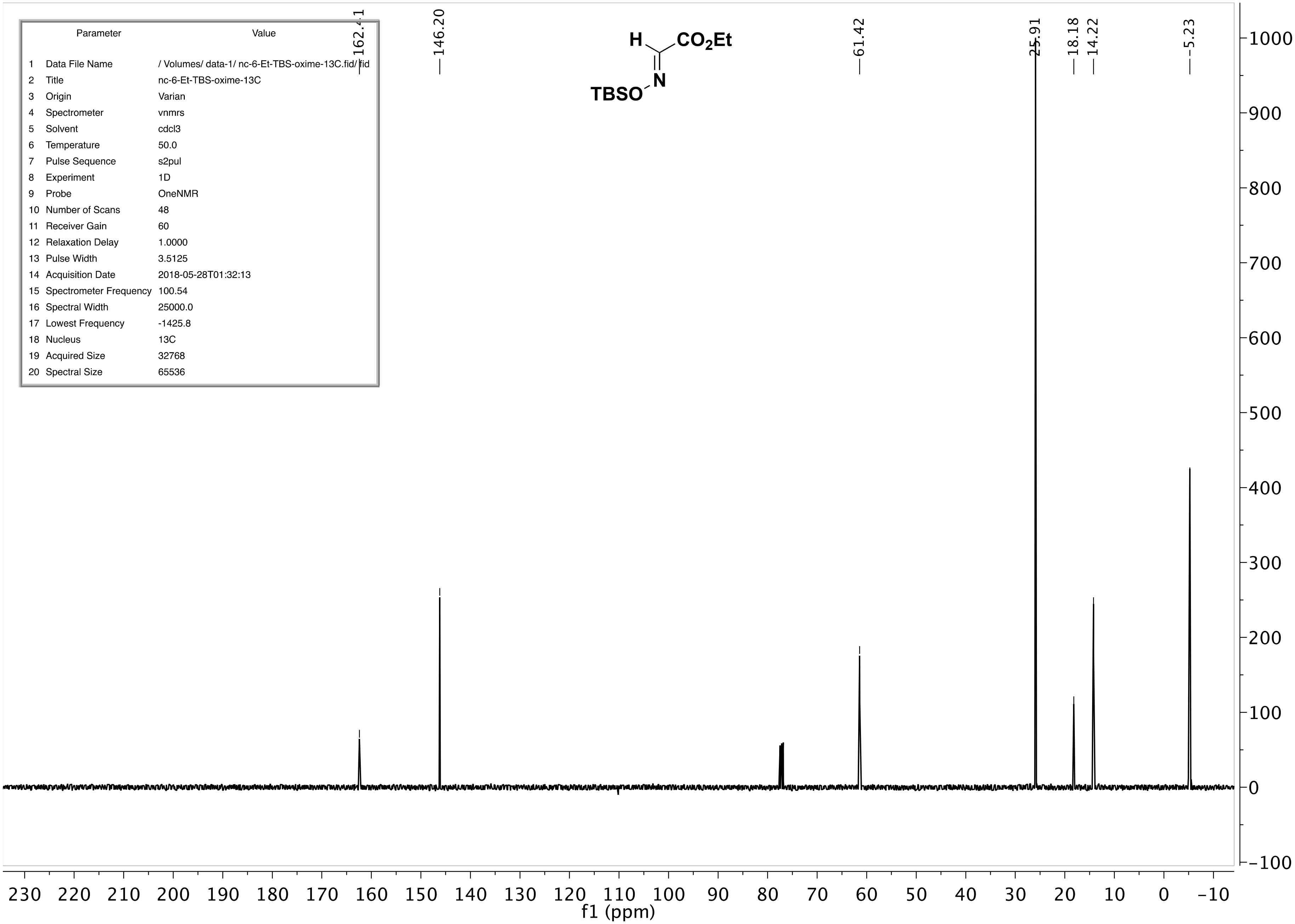

Parameter
Value
1 Data File Name / Volumes/ data-1/ nc-6-Et-TBS-oxime-13C.fid/ fid
2 Title nc-6-Et-TBS-oxime-13C
3 Origin Varian
4 Spectrometer vnmrs
5 Solvent cdcl3
6 Temperature 50.0
7 Pulse Sequence s2pul
8 Experiment 1D
9 Probe OneNMR
10 Number of Scans 48
11 Receiver Gain 60
12 Relaxation Delay 1.0000
13 Pulse Width 3.5125
14 Acquisition Date 2018-05-28T01:32:13
15 Spectrometer Frequency 100.54
16 Spectral Width 25000.0
17 Lowest Frequency -1425.8
18 Nucleus 13C
19 Acquired Size 32768
20 Spectral Size 65536
162.41
146.20
61.42
25.91
18.18
14.22
-5.23
H
CO2Et
N
TBSO
f1 (ppm)

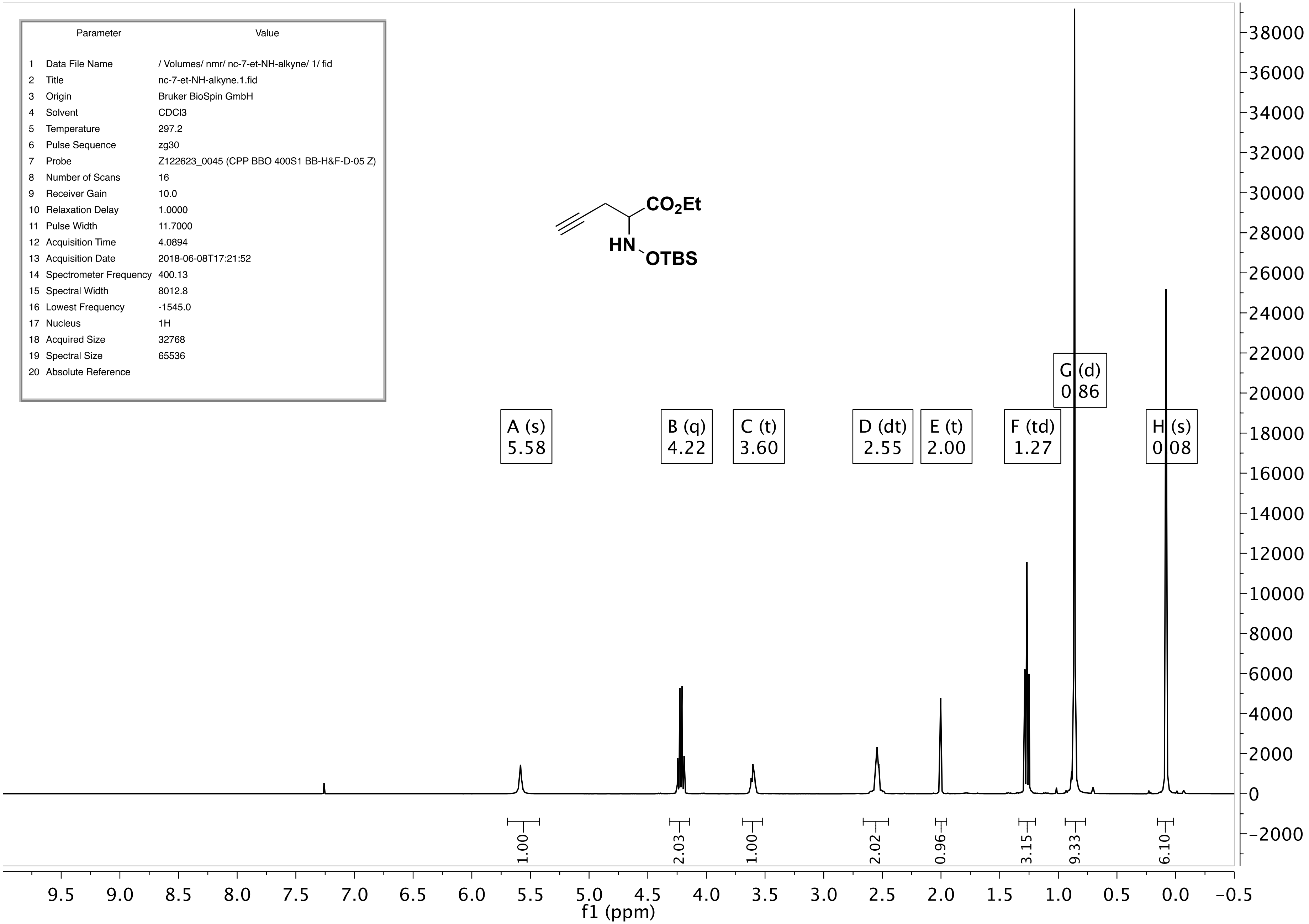

Parameter | Value
1 Data File Name | / Volumes/ nmr/ nc-7-et-NH-alkyne/ 1/ fid
2 Title | nc-7-et-NH-alkyne.1.fid
3 Origin | Bruker BioSpin GmbH
4 Solvent | CDCl3
5 Temperature | 297.2
6 Pulse Sequence | zg30
7 Probe | Z122623_0045 (CPP BBO 400S1 BB-H&F-D-05 Z)
8 Number of Scans | 16
9 Receiver Gain | 10.0
10 Relaxation Delay | 1.0000
11 Pulse Width | 11.7000
12 Acquisition Time | 4.0894
13 Acquisition Date | 2018-06-08T17:21:52
14 Spectrometer Frequency | 400.13
15 Spectral Width | 8012.8
16 Lowest Frequency | -1545.0
17 Nucleus | 1H
18 Acquired Size | 32768
19 Spectral Size | 65536
20 Absolute Reference
CO2Et
HN
OTBS
A (s) 5.58
B (q) 4.22
C (t) 3.60
D (dt) 2.55
E (t) 2.00
F (td) 1.27
G (d) 0.86
H (s) 0.08
1.00
2.03
1.00
2.02
0.96
3.15
9.33
6.10
f1 (ppm)
9.5 9.0 8.5 8.0 7.5 7.0 6.5 6.0 5.5 5.0 4.5 4.0 3.5 3.0 2.5 2.0 1.5 1.0 0.5 0.0 −0.5
38000 36000 34000 32000 30000 28000 26000 24000 22000 20000 18000 16000 14000 12000 10000 8000 6000 4000 2000 0 −2000

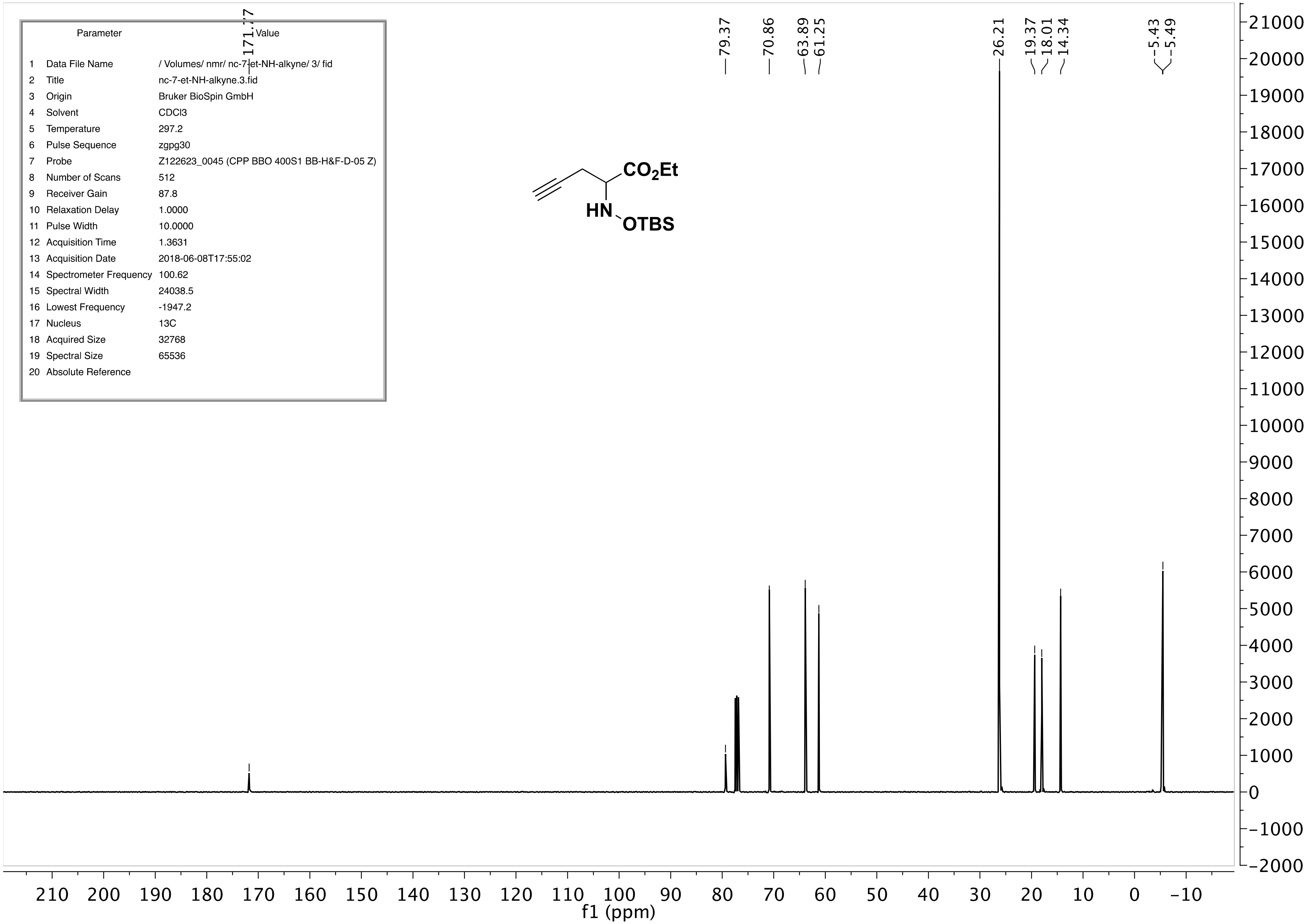
Parameter Value
1 Data File Name / Volumes/ nmr/ nc-7-et-NH-alkyne/ 3/ fid
2 Title nc-7-et-NH-alkyne.3.fid
3 Origin Bruker BioSpin GmbH
4 Solvent CDCl3
5 Temperature 297.2
6 Pulse Sequence zgpg30
7 Probe Z122623_0045 (CPP BBO 400S1 BB-H&F-D-05 Z)
8 Number of Scans 512
9 Receiver Gain 87.8
10 Relaxation Delay 1.0000
11 Pulse Width 10.0000
12 Acquisition Time 1.3631
13 Acquisition Date 2018-06-08T17:55:02
14 Spectrometer Frequency 100.62
15 Spectral Width 24038.5
16 Lowest Frequency -1947.2
17 Nucleus 13C
18 Acquired Size 32768
19 Spectral Size 65536
20 Absolute Reference
171.77
79.37
70.86
63.89
61.25
26.21
19.37
18.01
14.34
-5.43
-5.49
CO2Et
HN
OTBS
f1 (ppm)

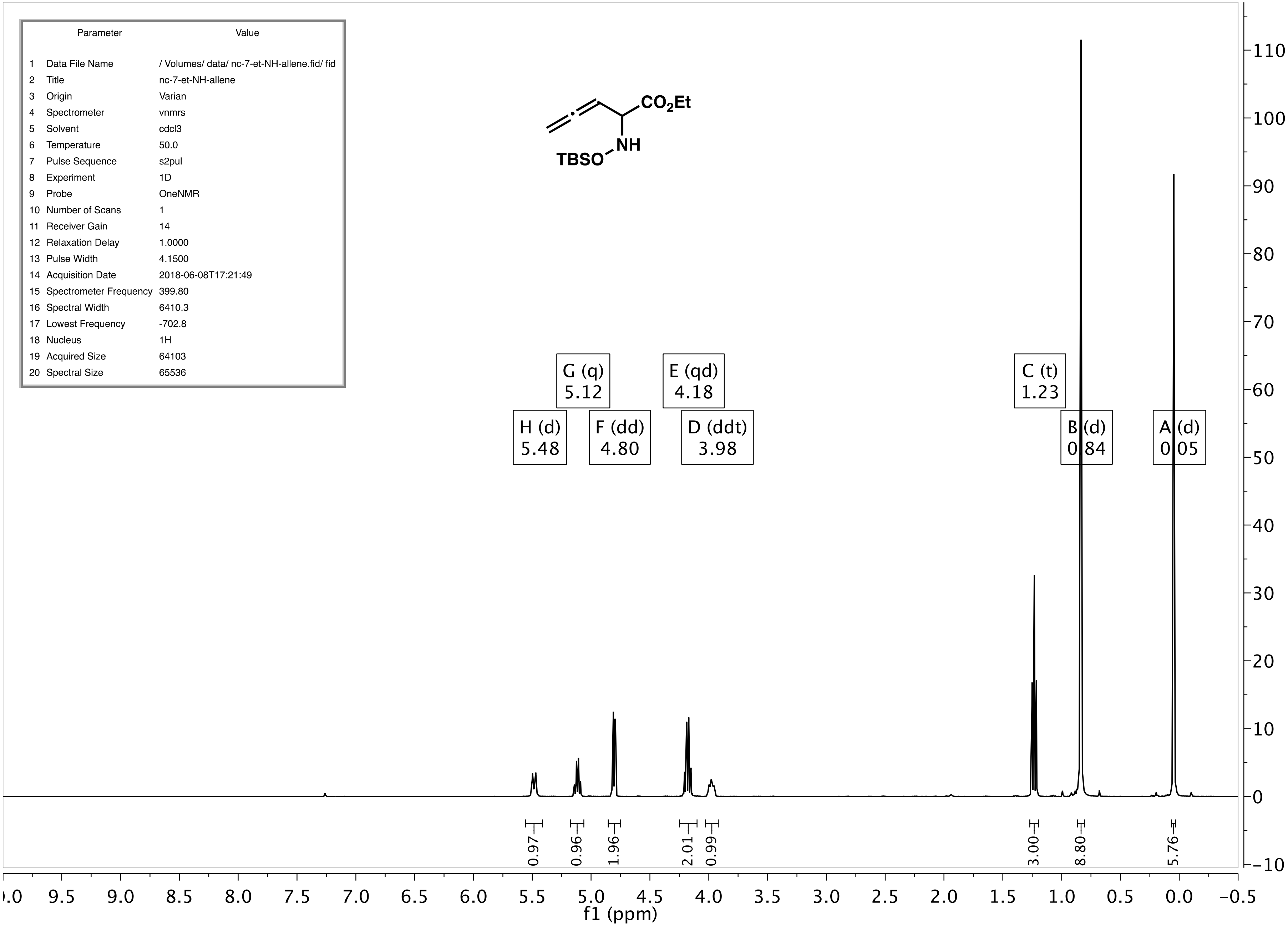

Parameter
Value
1 Data File Name / Volumes/ data/ nc-7-et-NH-allene.fid/ fid
2 Title nc-7-et-NH-allene
3 Origin Varian
4 Spectrometer vnmrs
5 Solvent cdcl3
6 Temperature 50.0
7 Pulse Sequence s2pul
8 Experiment 1D
9 Probe OneNMR
10 Number of Scans 1
11 Receiver Gain 14
12 Relaxation Delay 1.0000
13 Pulse Width 4.1500
14 Acquisition Date 2018-06-08T17:21:49
15 Spectrometer Frequency 399.80
16 Spectral Width 6410.3
17 Lowest Frequency -702.8
18 Nucleus 1H
19 Acquired Size 64103
20 Spectral Size 65536
CO2Et
NH
TBSO
G (q) 5.12
E (qd) 4.18
H (d) 5.48
F (dd) 4.80
D (ddt) 3.98
C (t) 1.23
B (d) 0.84
A (d) 0.05
0.97
0.96
1.96
2.01
0.99
3.00
8.80
5.76
f1 (ppm)

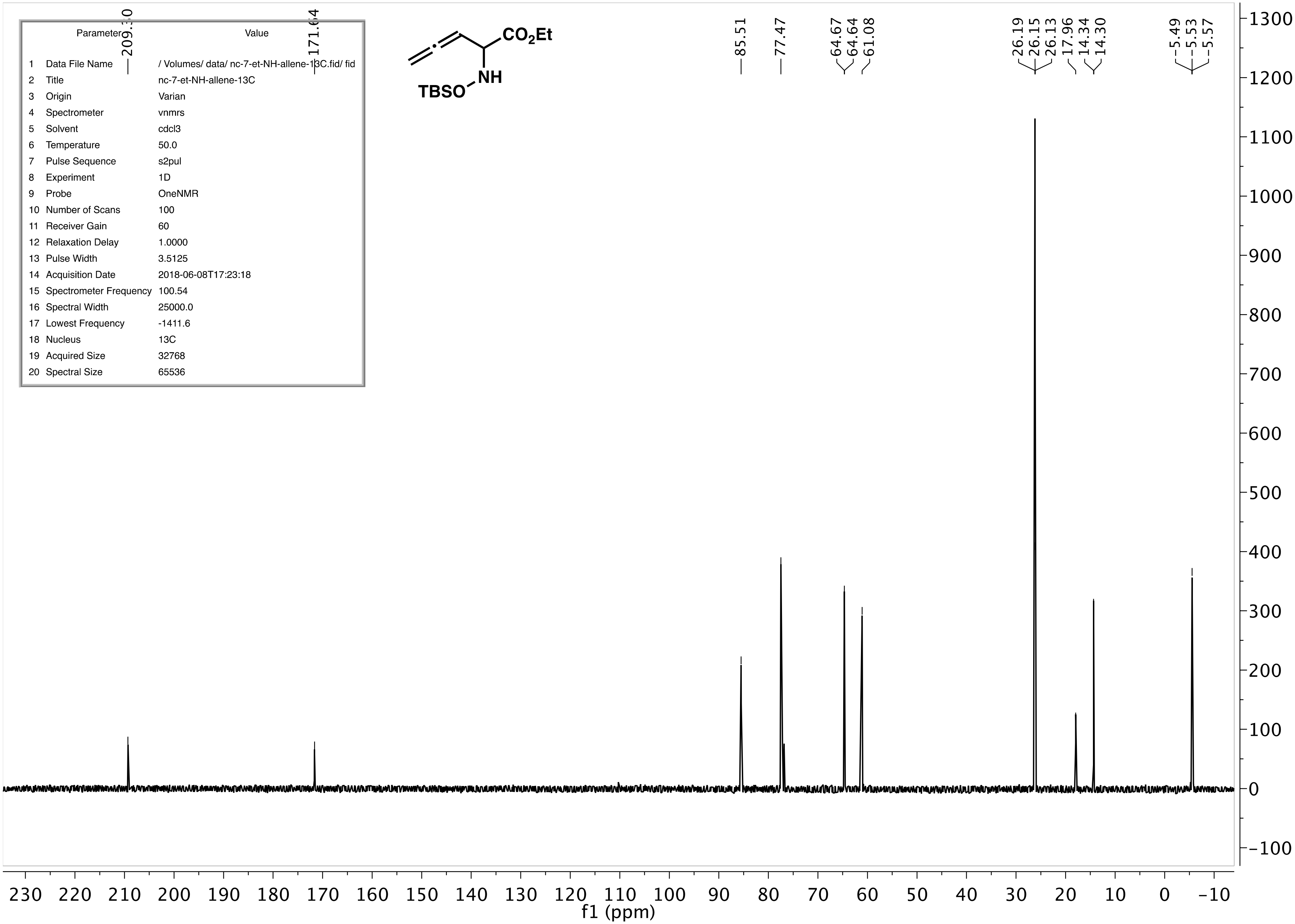
Parameter Value
1 Data File Name / Volumes/ data/ nc-7-et-NH-allene-13C.fid/ fid
2 Title nc-7-et-NH-allene-13C
3 Origin Varian
4 Spectrometer vnmrs
5 Solvent cdcl3
6 Temperature 50.0
7 Pulse Sequence s2pul
8 Experiment 1D
9 Probe OneNMR
10 Number of Scans 100
11 Receiver Gain 60
12 Relaxation Delay 1.0000
13 Pulse Width 3.5125
14 Acquisition Date 2018-06-08T17:23:18
15 Spectrometer Frequency 100.54
16 Spectral Width 25000.0
17 Lowest Frequency -1411.6
18 Nucleus 13C
19 Acquired Size 32768
20 Spectral Size 65536
CO_2Et
NH
TBSO
209.30
171.64
85.51
77.47
64.67
64.64
61.08
26.19
26.15
26.13
17.96
14.34
14.30
-5.49
-5.53
-5.57
f1 (ppm)

Parameter	Value
1 Data File Name	/ Volumes/ data/ nc-7-et-NH-allene-HSQC.fid/ fid
2 Title	nc-7-et-NH-allene-HSQC
3 Origin	Varian
4 Spectrometer	vnmrs
5 Solvent	cdcl3
6 Temperature	50.0
7 Pulse Sequence	HSQCAD
8 Experiment	HSQC-EDITED
9 Probe	OneNMR
10 Number of Scans	2
11 Receiver Gain	30
12 Relaxation Delay	1.0000
13 Pulse Width	8.3000
14 Acquisition Date	2018-06-08T17:35:56
15 Spectrometer Frequency	(399.80, 100.54)
16 Spectral Width	(6410.3, 20105.6)
17 Lowest Frequency	(-738.4, -1005.2)
18 Nucleus	(1H, 13C)
19 Acquired Size	(962, 96)
20 Spectral Size	(1024, 1024)

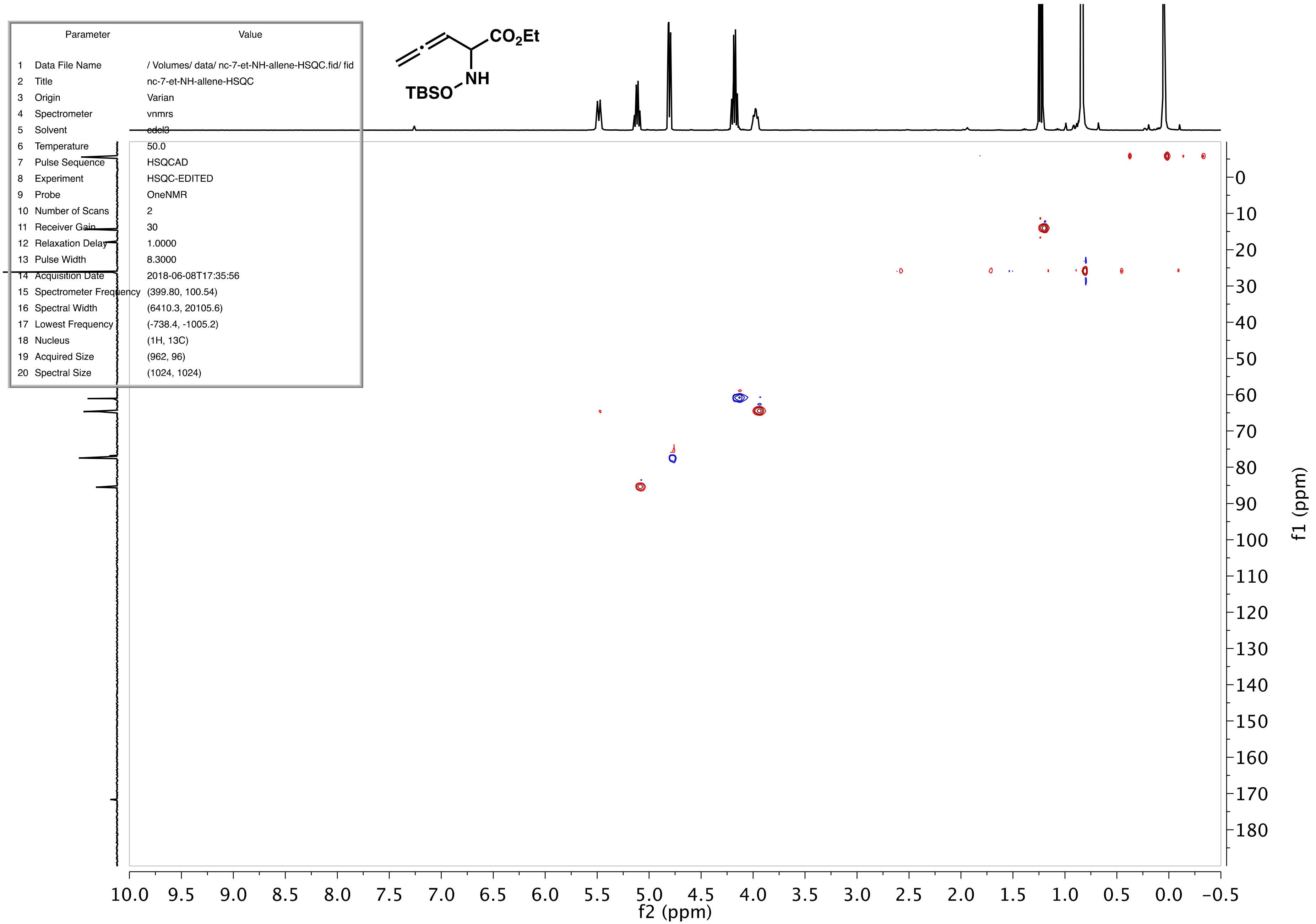

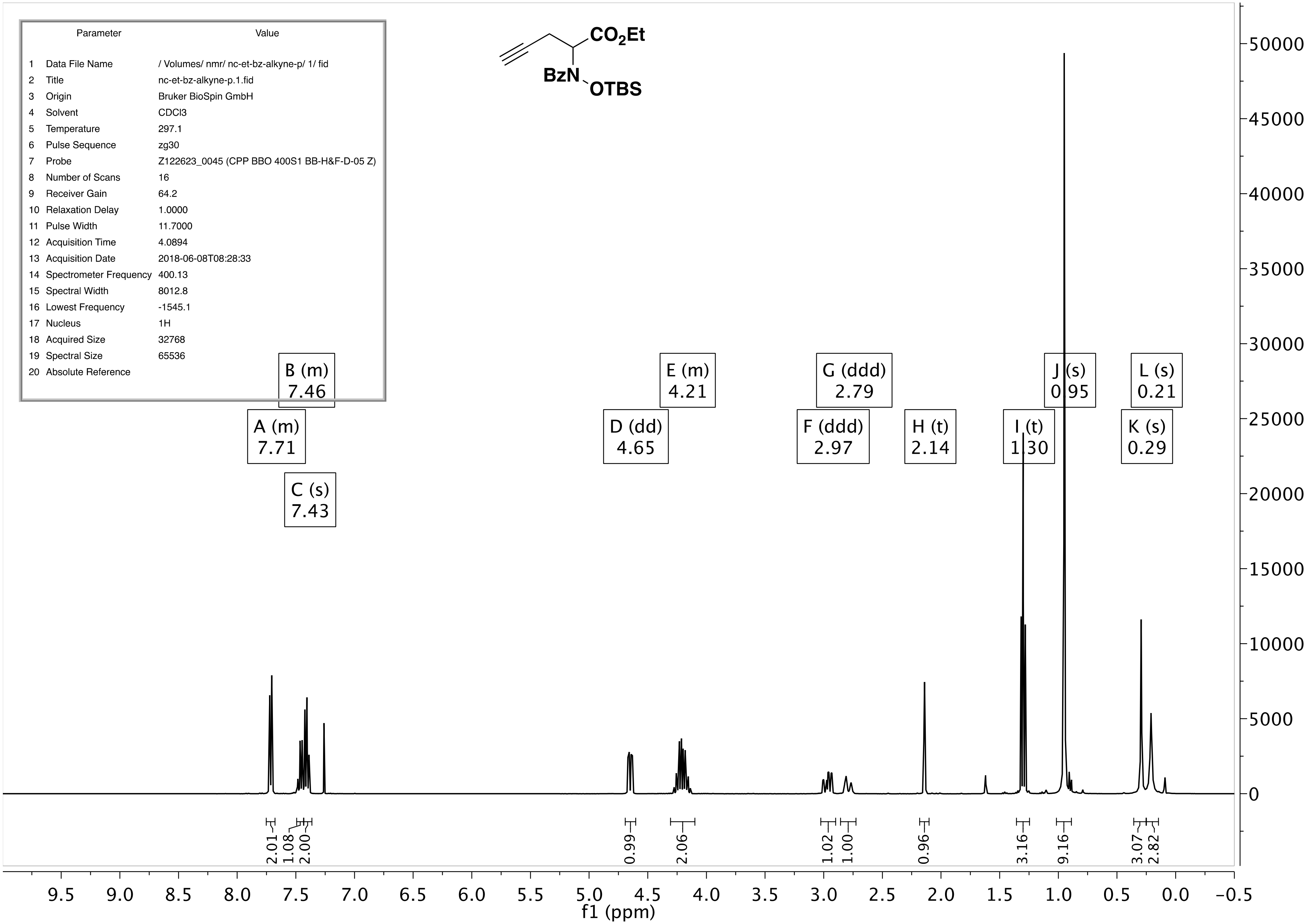
Parameter Value
1 Data File Name / Volumes/ nmr/ nc-et-bz-alkyne-p/ 1/ fid
2 Title nc-et-bz-alkyne-p.1.fid
3 Origin Bruker BioSpin GmbH
4 Solvent CDCl3
5 Temperature 297.1
6 Pulse Sequence zg30
7 Probe Z122623_0045 (CPP BBO 400S1 BB-H&F-D-05 Z)
8 Number of Scans 16
9 Receiver Gain 64.2
10 Relaxation Delay 1.0000
11 Pulse Width 11.7000
12 Acquisition Time 4.0894
13 Acquisition Date 2018-06-08T08:28:33
14 Spectrometer Frequency 400.13
15 Spectral Width 8012.8
16 Lowest Frequency -1545.1
17 Nucleus 1H
18 Acquired Size 32768
19 Spectral Size 65536
20 Absolute Reference
CO2Et
BzN
OTBS
A (m) 7.71
B (m) 7.46
C (s) 7.43
D (dd) 4.65
E (m) 4.21
F (ddd) 2.97
G (ddd) 2.79
H (t) 2.14
I (t) 1.30
J (s) 0.95
K (s) 0.29
L (s) 0.21
2.01
1.08
2.00
0.99
2.06
1.02
1.00
0.96
3.16
9.16
3.07
2.82
f1 (ppm)

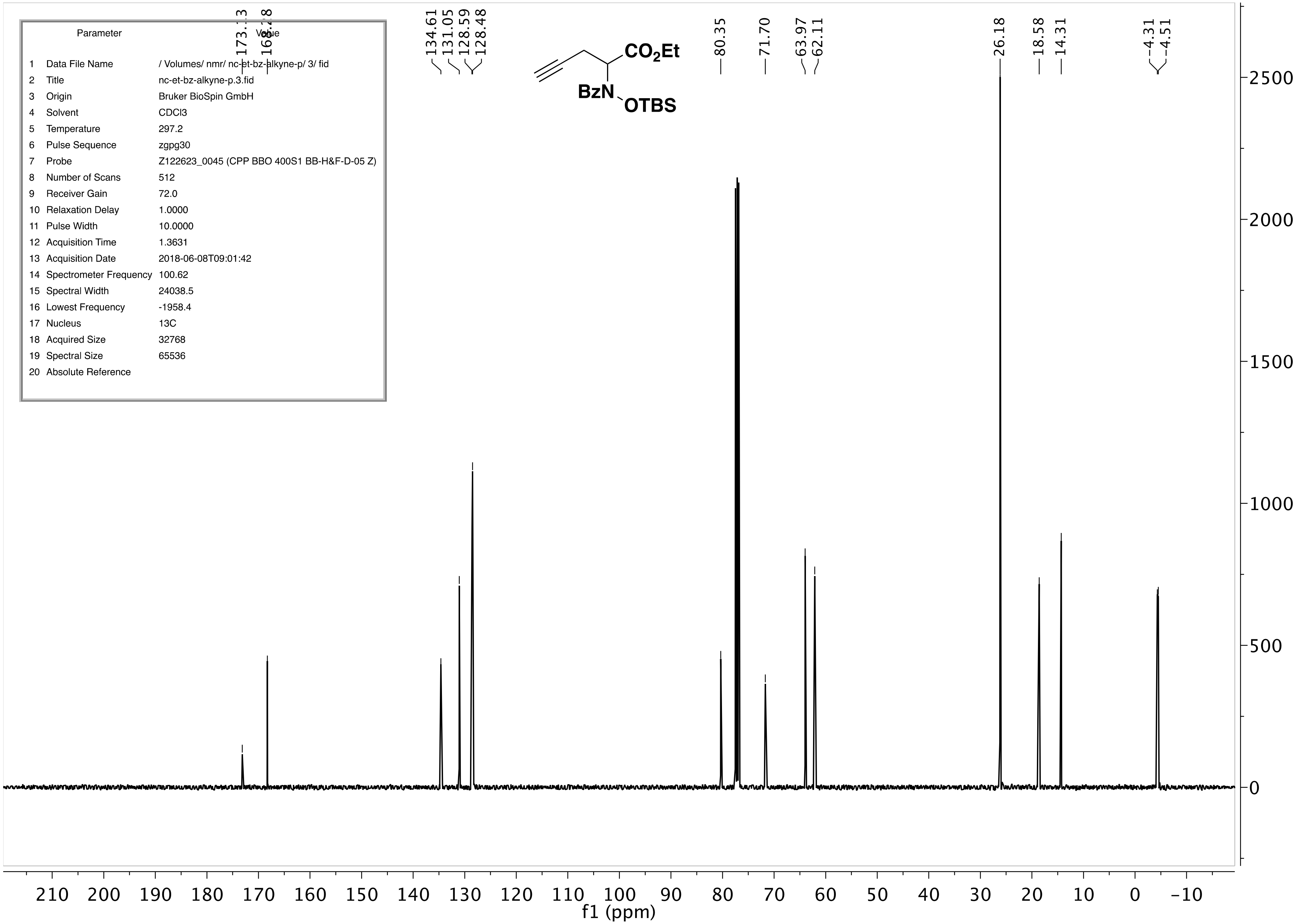

134.61
131.05
128.59
128.48
80.35
71.70
63.97
62.11
26.18
18.58
14.31
-4.31
-4.51
CO2Et
BzN
OTBS
Parameter
1 Data File Name / Volumes/ nmr/ nc-et-bz-alkyne-p/ 3/ fid
2 Title nc-et-bz-alkyne-p.3.fid
3 Origin Bruker BioSpin GmbH
4 Solvent CDCl3
5 Temperature 297.2
6 Pulse Sequence zgpg30
7 Probe Z122623_0045 (CPP BBO 400S1 BB-H&F-D-05 Z)
8 Number of Scans 512
9 Receiver Gain 72.0
10 Relaxation Delay 1.0000
11 Pulse Width 10.0000
12 Acquisition Time 1.3631
13 Acquisition Date 2018-06-08T09:01:42
14 Spectrometer Frequency 100.62
15 Spectral Width 24038.5
16 Lowest Frequency -1958.4
17 Nucleus 13C
18 Acquired Size 32768
19 Spectral Size 65536
20 Absolute Reference
f1 (ppm)

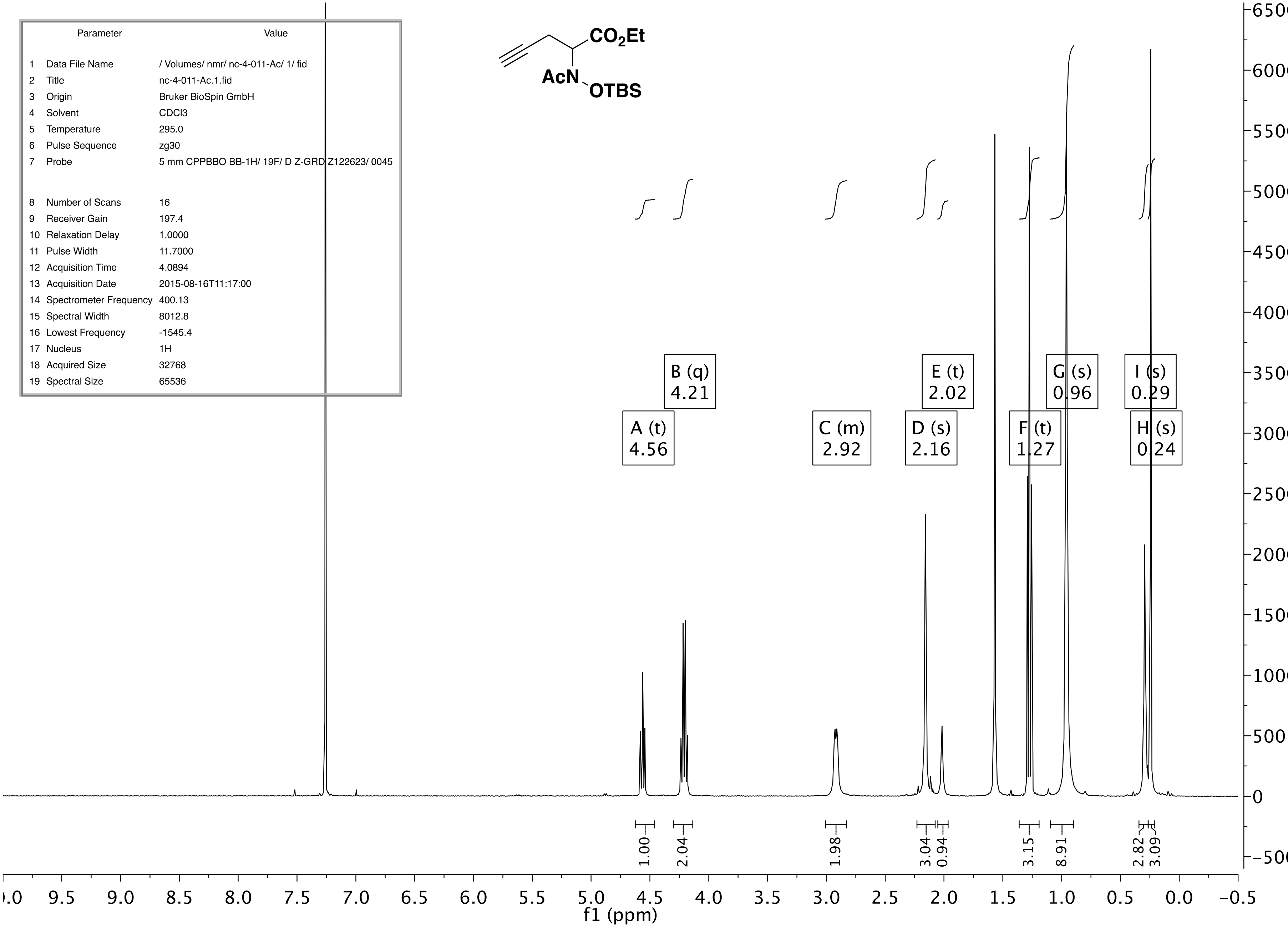
CO2Et
AcN
OTBS
Parameter Value
1 Data File Name / Volumes/ nmr/ nc-4-011-Ac/ 1/ fid
2 Title nc-4-011-Ac.1.fid
3 Origin Bruker BioSpin GmbH
4 Solvent CDCl3
5 Temperature 295.0
6 Pulse Sequence zg30
7 Probe 5 mm CPPBBO BB-1H/ 19F/ D Z-GRD Z122623/ 0045
8 Number of Scans 16
9 Receiver Gain 197.4
10 Relaxation Delay 1.0000
11 Pulse Width 11.7000
12 Acquisition Time 4.0894
13 Acquisition Date 2015-08-16T11:17:00
14 Spectrometer Frequency 400.13
15 Spectral Width 8012.8
16 Lowest Frequency -1545.4
17 Nucleus 1H
18 Acquired Size 32768
19 Spectral Size 65536
A (t) 4.56
B (q) 4.21
C (m) 2.92
D (s) 2.16
E (t) 2.02
F (t) 1.27
G (s) 0.96
H (s) 0.24
I (s) 0.29
1.00
2.04
1.98
3.04
0.94
3.15
8.91
2.82
3.09
f1 (ppm)

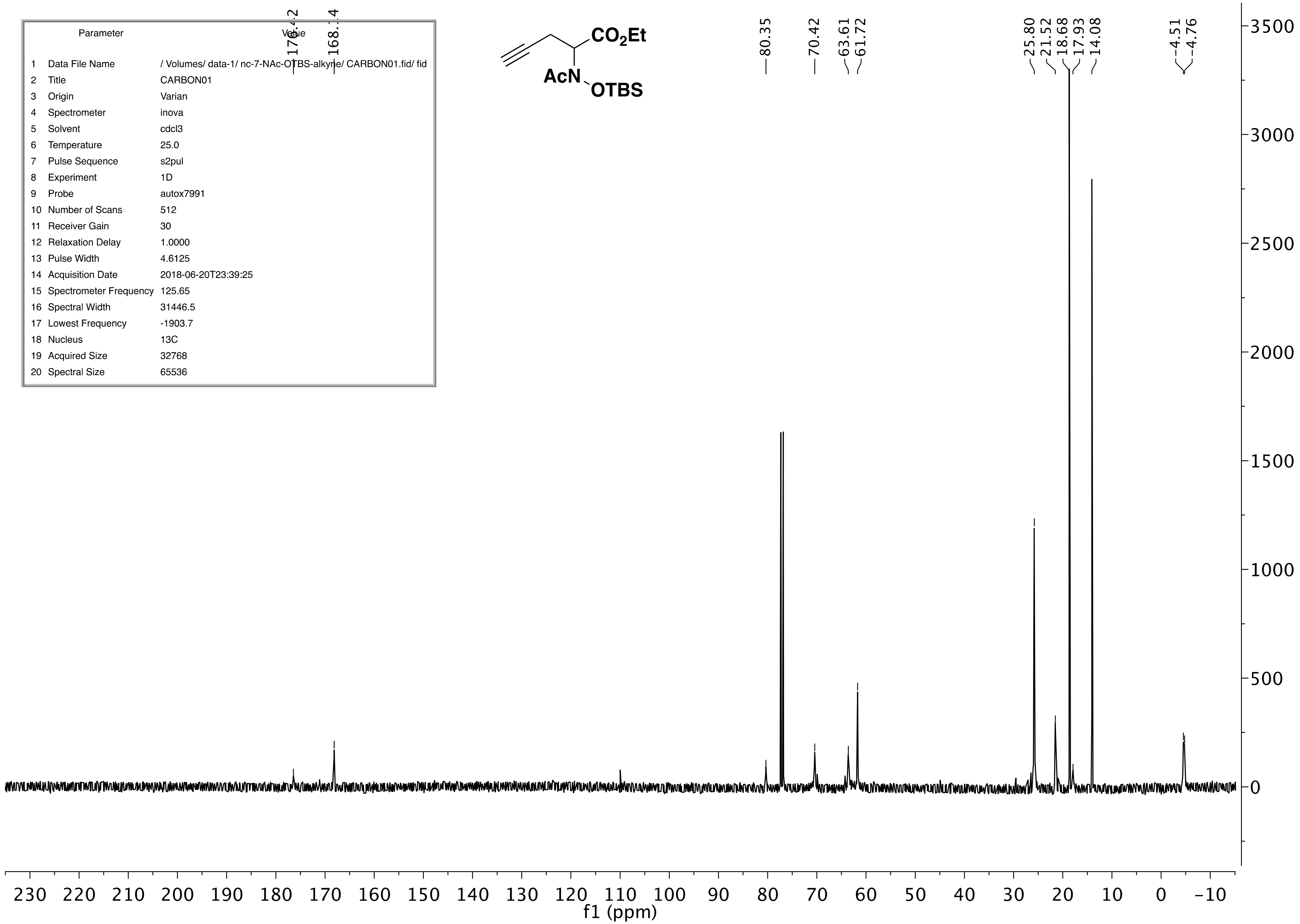

	Parameter	Value
1	Data File Name	/ Volumes/ data-1/ nc-7-NAc-OTBS-alkyne/ CARBON01.fid/ fid
2	Title	CARBON01
3	Origin	Varian
4	Spectrometer	inova
5	Solvent	cdcl3
6	Temperature	25.0
7	Pulse Sequence	s2pul
8	Experiment	1D
9	Probe	autox7991
10	Number of Scans	512
11	Receiver Gain	30
12	Relaxation Delay	1.0000
13	Pulse Width	4.6125
14	Acquisition Date	2018-06-20T23:39:25
15	Spectrometer Frequency	125.65
16	Spectral Width	31446.5
17	Lowest Frequency	-1903.7
18	Nucleus	13C
19	Acquired Size	32768
20	Spectral Size	65536

	Parameter	Value
1	Data File Name	/ Volumes/ nmrdata/ ncowper/ vnmrsys/ data/ nc-et-bz-diene-1H.fid/ fid
2	Title	nc-et-bz-diene-1H
3	Origin	Varian
4	Spectrometer	vnmrs
5	Solvent	cdcl3
6	Temperature	50.0
7	Pulse Sequence	s2pul
8	Experiment	1D
9	Probe	OneNMR
10	Number of Scans	1
11	Receiver Gain	22
12	Relaxation Delay	1.0000
13	Pulse Width	4.1500
14	Acquisition Date	2018-05-31T22:47:53
15	Spectrometer Frequency	399.80
16	Spectral Width	6410.3
17	Lowest Frequency	-805.6
18	Nucleus	1H
19	Acquired Size	64103
20	Spectral Size	65536

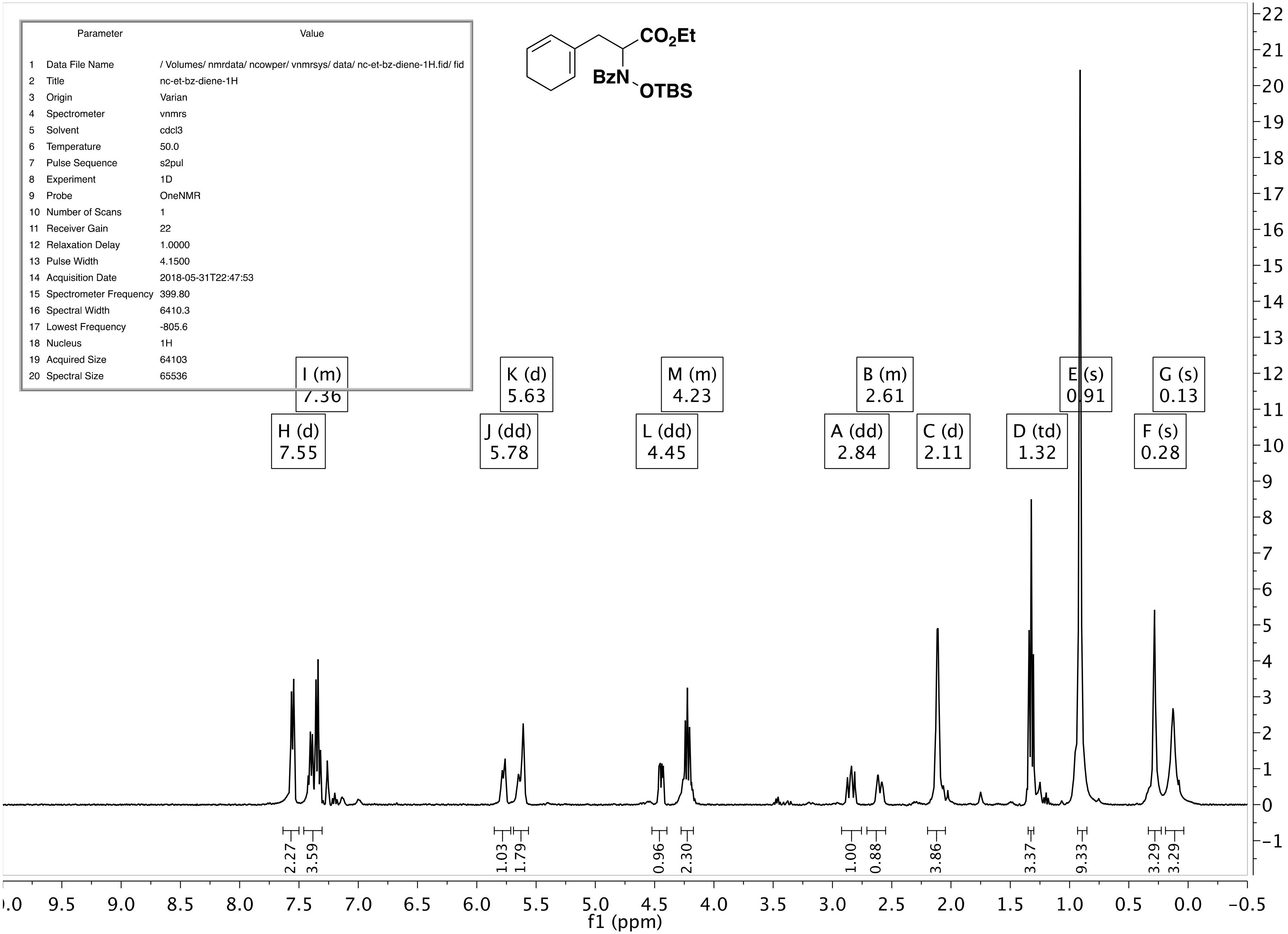

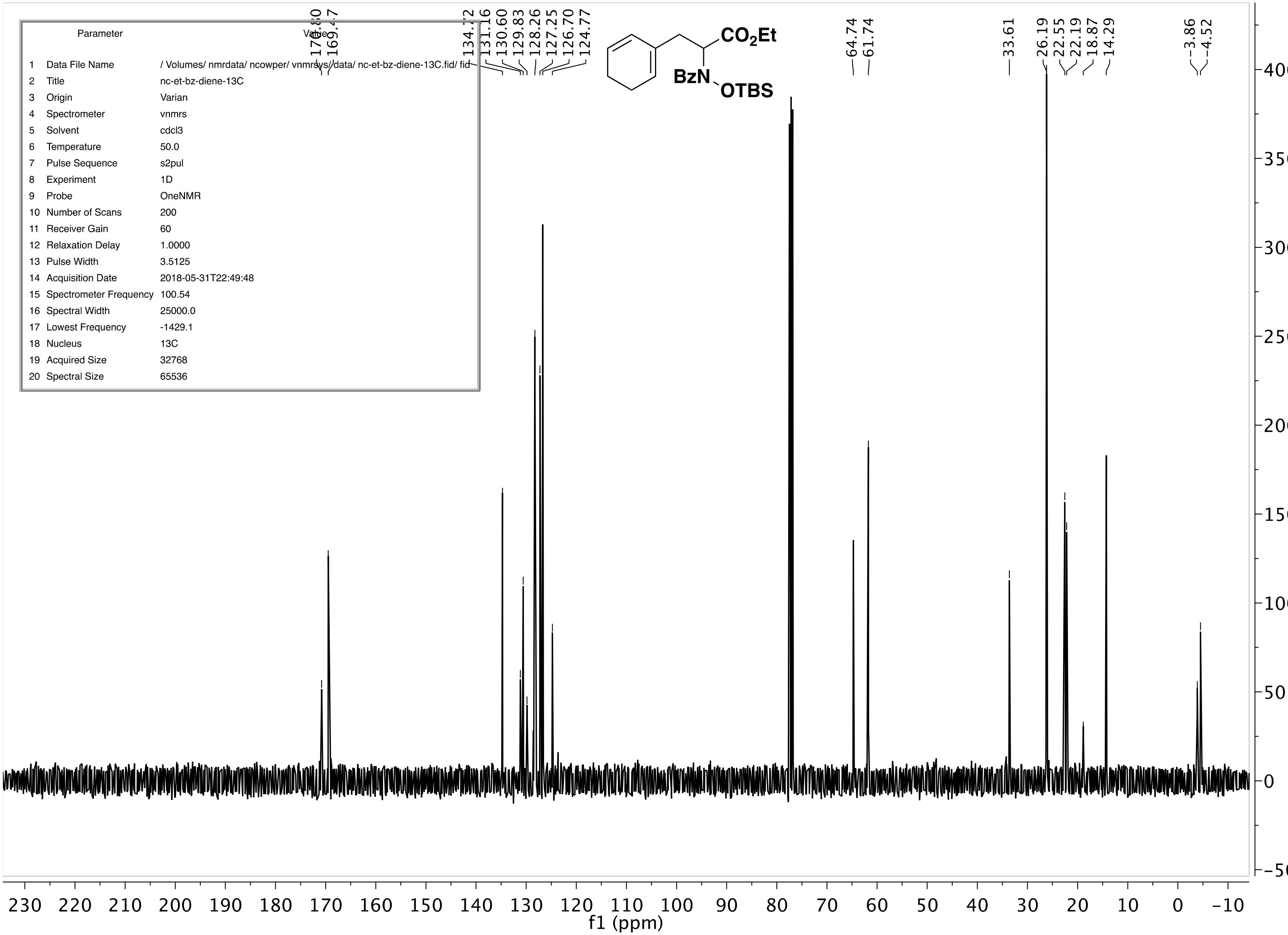
Parameter
Value
1 Data File Name / Volumes/ nmrdata/ ncowper/ vnmrsys/ data/ nc-et-bz-diene-13C.fid/ fid
2 Title nc-et-bz-diene-13C
3 Origin Varian
4 Spectrometer vnmrs
5 Solvent cdcl3
6 Temperature 50.0
7 Pulse Sequence s2pul
8 Experiment 1D
9 Probe OneNMR
10 Number of Scans 200
11 Receiver Gain 60
12 Relaxation Delay 1.0000
13 Pulse Width 3.5125
14 Acquisition Date 2018-05-31T22:49:48
15 Spectrometer Frequency 100.54
16 Spectral Width 25000.0
17 Lowest Frequency -1429.1
18 Nucleus 13C
19 Acquired Size 32768
20 Spectral Size 65536
CO2Et
BzN
OTBS
169.47
134.72
131.16
130.60
129.83
128.26
127.25
126.70
124.77
64.74
61.74
33.61
26.19
22.55
22.19
18.87
14.29
-3.86
-4.52
f1 (ppm)

	Parameter	Value
1	Data File Name	/ Volumes/ data/ nc-7-Ac-Et-Diene-cle/ PROTON01.fid/ fid
2	Title	PROTON01
3	Origin	Varian
4	Spectrometer	inova
5	Solvent	cdcl3
6	Temperature	25.0
7	Pulse Sequence	s2pul
8	Experiment	1D
9	Probe	autox7991
10	Number of Scans	8
11	Receiver Gain	32
12	Relaxation Delay	1.0000
13	Pulse Width	5.8000
14	Acquisition Date	2018-06-30T20:17:52
15	Spectrometer Frequency	499.64
16	Spectral Width	8000.0
17	Lowest Frequency	-1029.7
18	Nucleus	1H
19	Acquired Size	24000
20	Spectral Size	65536

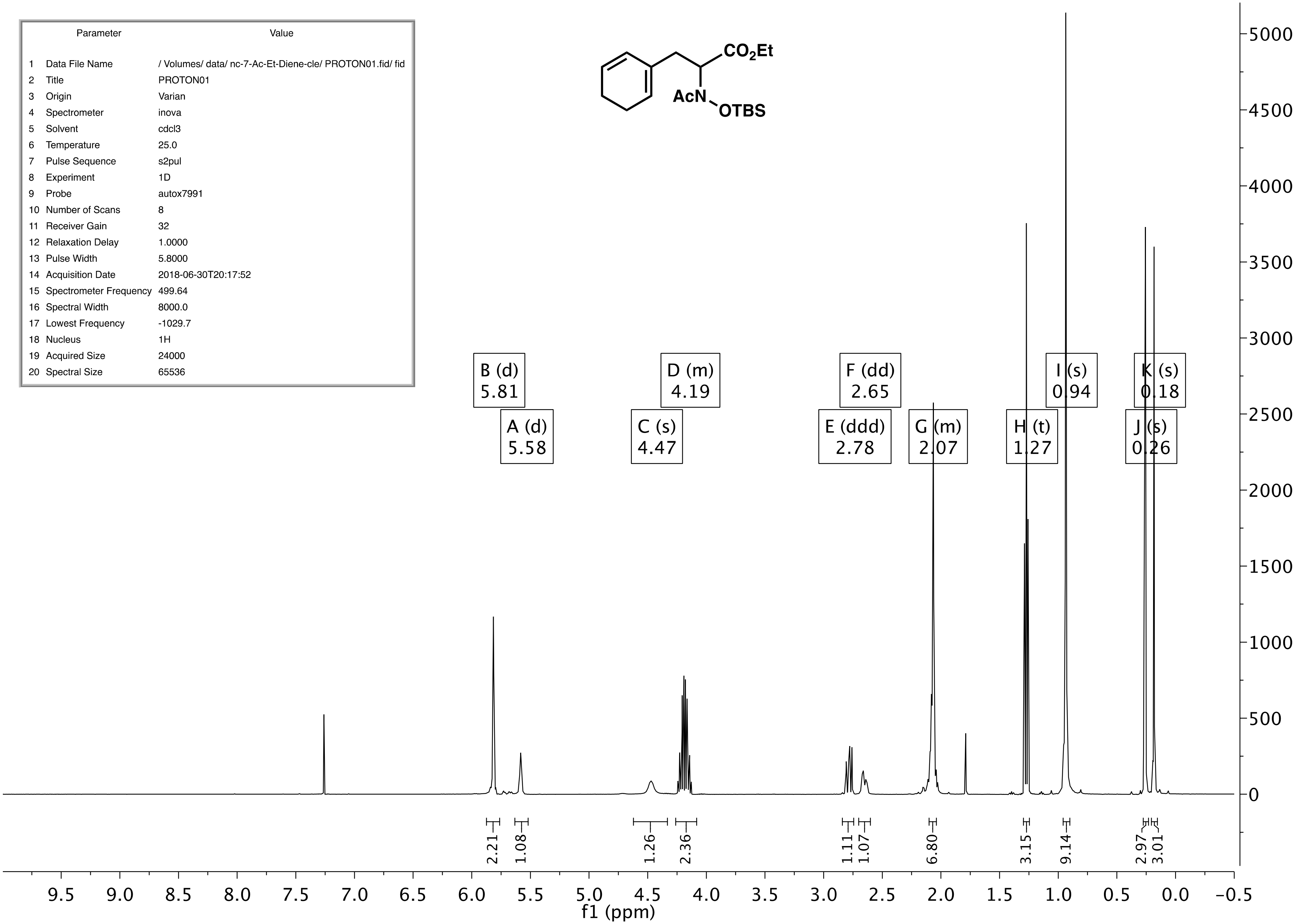

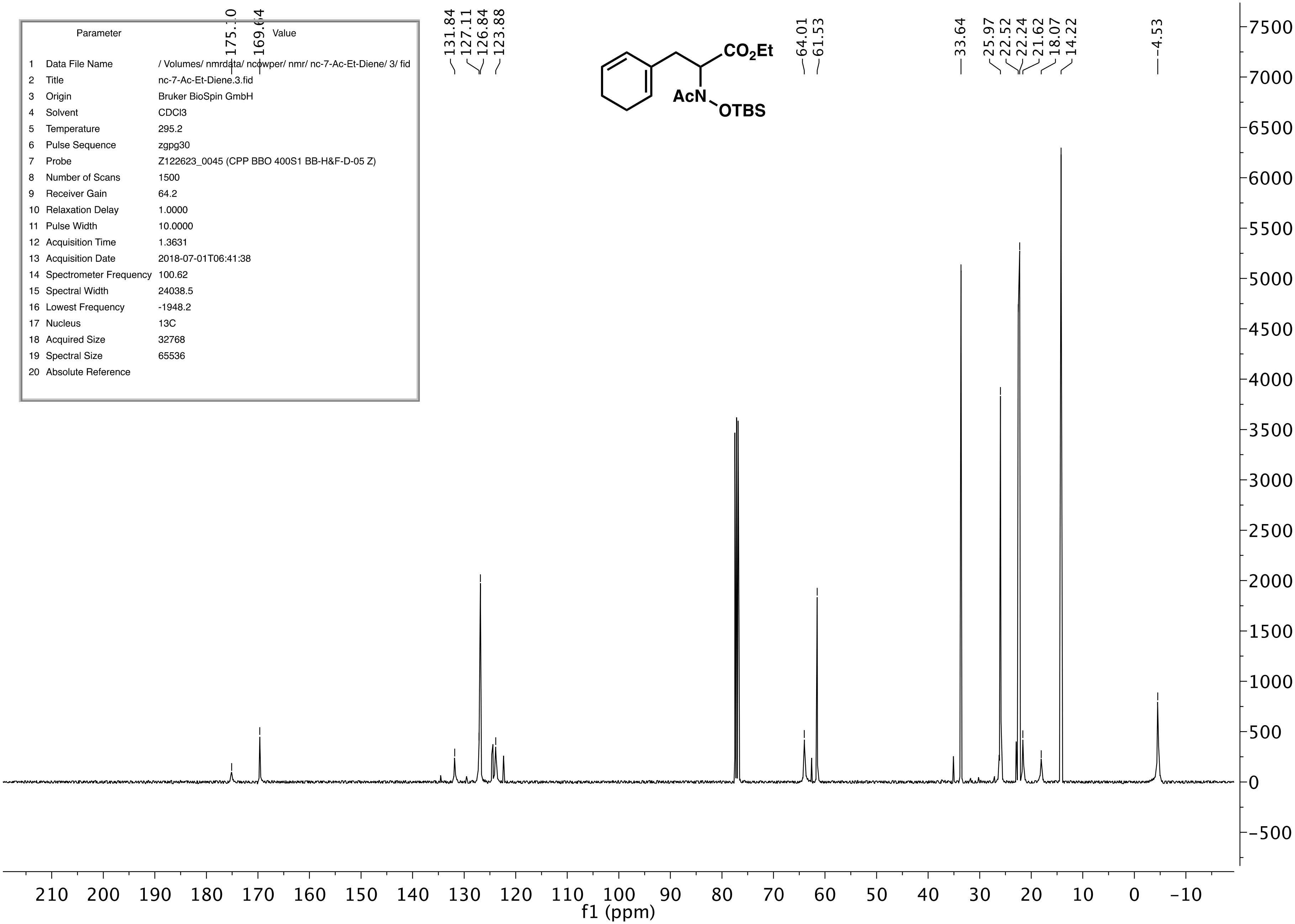
Parameter Value
1 Data File Name / Volumes/ nmrdata/ ncowper/ nmr/ nc-7-Ac-Et-Diene/ 3/ fid
2 Title nc-7-Ac-Et-Diene.3.fid
3 Origin Bruker BioSpin GmbH
4 Solvent CDCl3
5 Temperature 295.2
6 Pulse Sequence zgpg30
7 Probe Z122623_0045 (CPP BBO 400S1 BB-H&F-D-05 Z)
8 Number of Scans 1500
9 Receiver Gain 64.2
10 Relaxation Delay 1.0000
11 Pulse Width 10.0000
12 Acquisition Time 1.3631
13 Acquisition Date 2018-07-01T06:41:38
14 Spectrometer Frequency 100.62
15 Spectral Width 24038.5
16 Lowest Frequency -1948.2
17 Nucleus 13C
18 Acquired Size 32768
19 Spectral Size 65536
20 Absolute Reference
169.64
131.84
127.11
126.84
123.88
CO2Et
AcN
OTBS
64.01
61.53
33.64
25.97
22.52
22.24
21.62
18.07
14.22
-4.53
f1 (ppm)

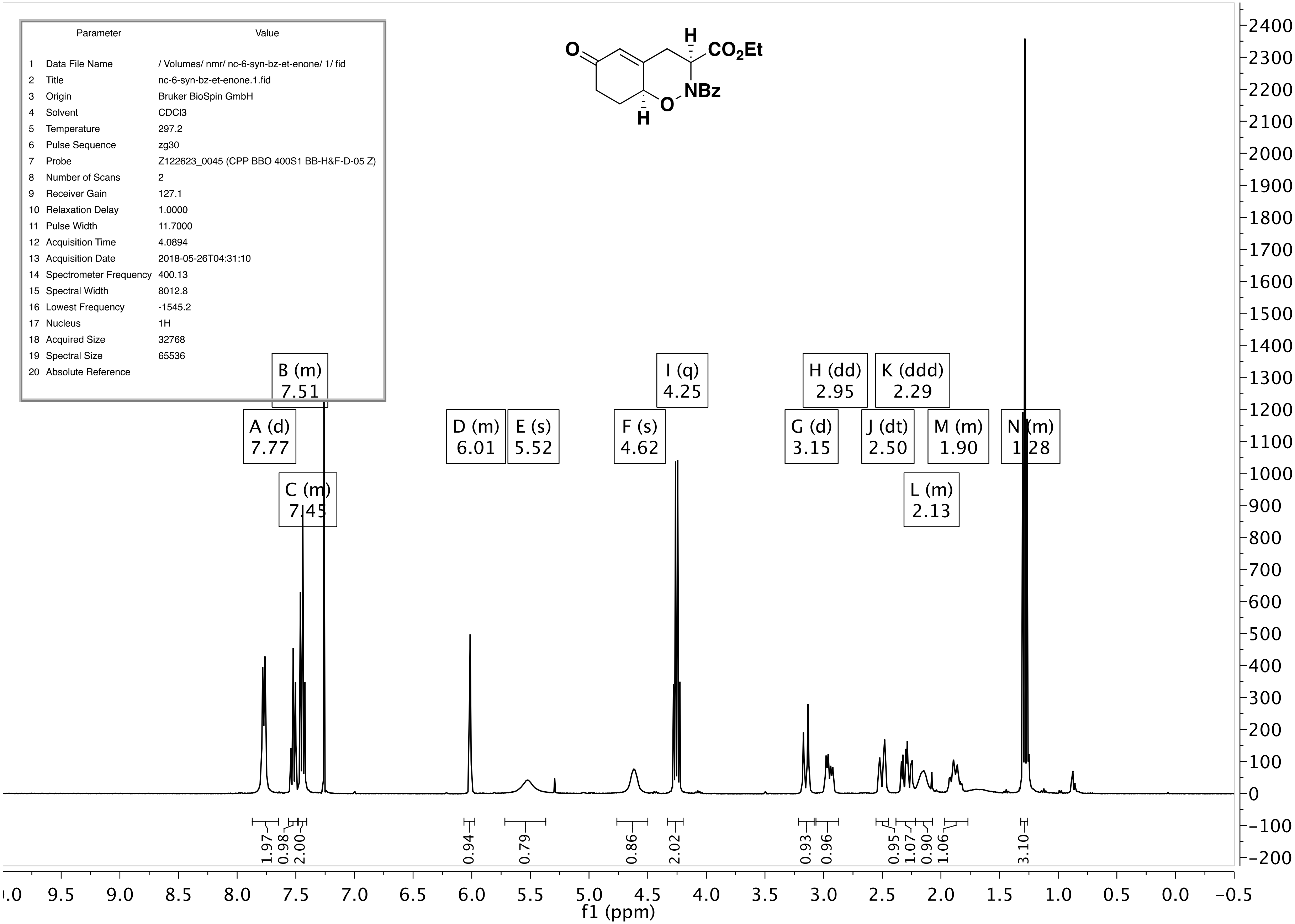

Parameter
Value
1 Data File Name / Volumes/ nmr/ nc-6-syn-bz-et-enone/ 1/ fid
2 Title nc-6-syn-bz-et-enone.1.fid
3 Origin Bruker BioSpin GmbH
4 Solvent CDCl3
5 Temperature 297.2
6 Pulse Sequence zg30
7 Probe Z122623_0045 (CPP BBO 400S1 BB-H&F-D-05 Z)
8 Number of Scans 2
9 Receiver Gain 127.1
10 Relaxation Delay 1.0000
11 Pulse Width 11.7000
12 Acquisition Time 4.0894
13 Acquisition Date 2018-05-26T04:31:10
14 Spectrometer Frequency 400.13
15 Spectral Width 8012.8
16 Lowest Frequency -1545.2
17 Nucleus 1H
18 Acquired Size 32768
19 Spectral Size 65536
20 Absolute Reference
O
H
CO2Et
NBz
O
H
A (d) 7.77
B (m) 7.51
C (m) 7.45
D (m) 6.01
E (s) 5.52
F (s) 4.62
I (q) 4.25
G (d) 3.15
H (dd) 2.95
J (dt) 2.50
K (ddd) 2.29
L (m) 2.13
M (m) 1.90
N (m) 1.28
1.97
0.98
2.00
0.94
0.79
0.86
2.02
0.93
0.96
0.95
1.07
0.90
1.06
3.10
f1 (ppm)

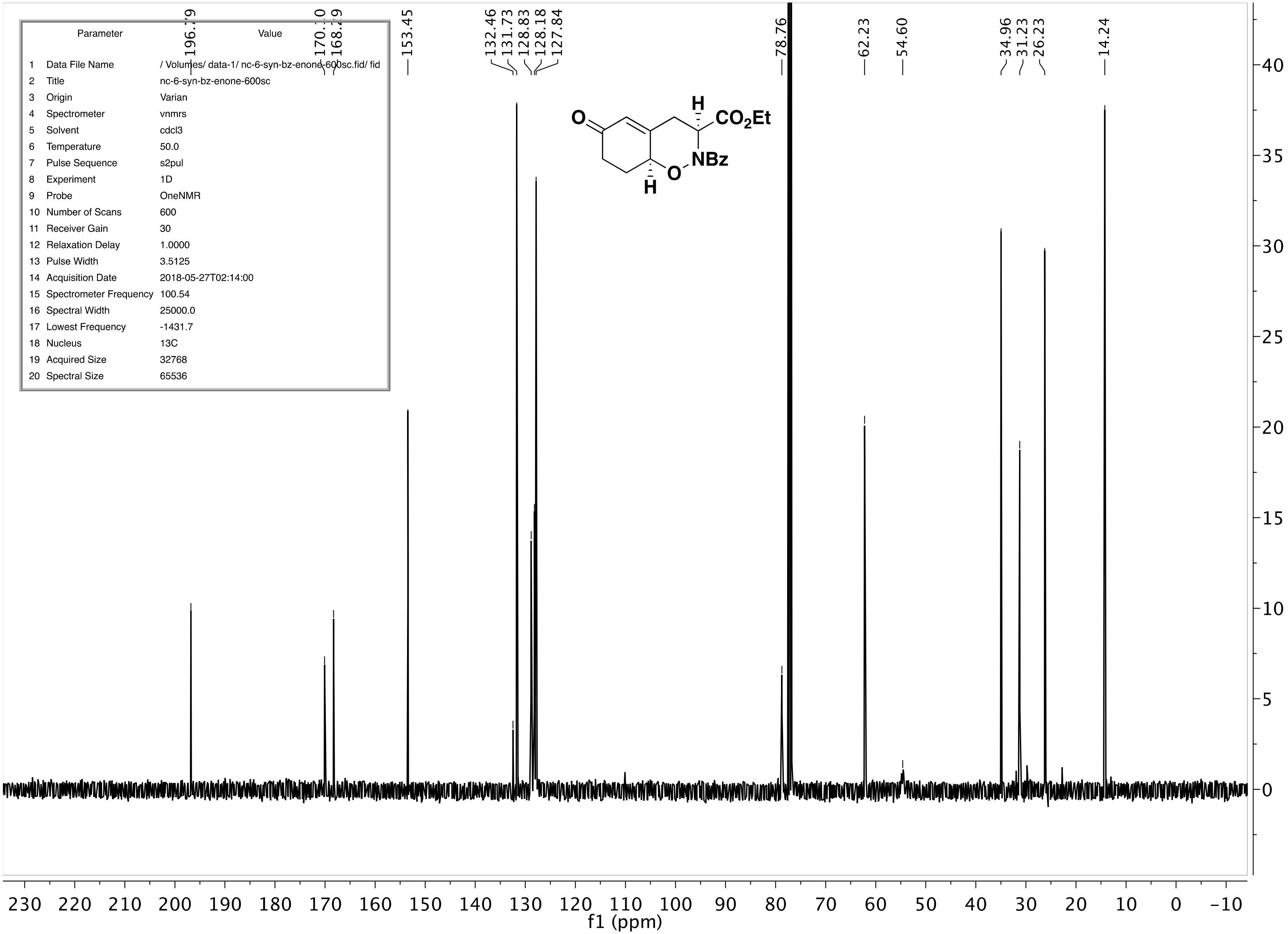
Parameter
Value
1 Data File Name / Volumes/ data-1/ nc-6-syn-bz-enone-600sc.fid/ fid
2 Title nc-6-syn-bz-enone-600sc
3 Origin Varian
4 Spectrometer vnmrs
5 Solvent cdcl3
6 Temperature 50.0
7 Pulse Sequence s2pul
8 Experiment 1D
9 Probe OneNMR
10 Number of Scans 600
11 Receiver Gain 30
12 Relaxation Delay 1.0000
13 Pulse Width 3.5125
14 Acquisition Date 2018-05-27T02:14:00
15 Spectrometer Frequency 100.54
16 Spectral Width 25000.0
17 Lowest Frequency -1431.7
18 Nucleus 13C
19 Acquired Size 32768
20 Spectral Size 65536
196.79
168.29
153.45
132.46
131.73
128.83
128.18
127.84
78.76
62.23
54.60
34.96
31.23
26.23
14.24
O
H
CO2Et
NBz
O
H
40
35
30
25
20
15
10
5
0
230 220 210 200 190 180 170 160 150 140 130 120 110 100 90 80 70 60 50 40 30 20 10 0 -10
f1 (ppm)

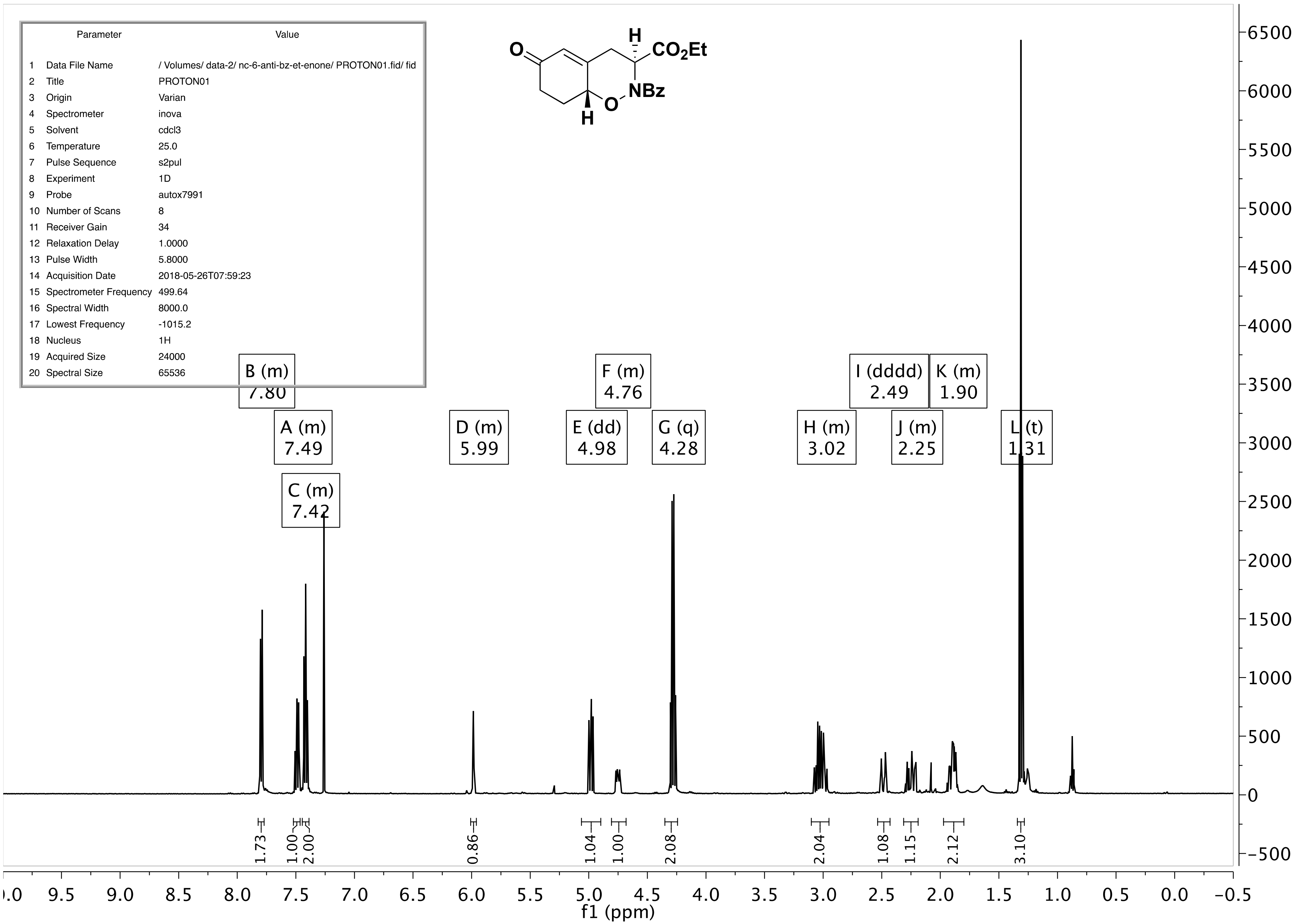
Parameter Value
1 Data File Name / Volumes/ data-2/ nc-6-anti-bz-et-enone/ PROTON01.fid/ fid
2 Title PROTON01
3 Origin Varian
4 Spectrometer inova
5 Solvent cdcl3
6 Temperature 25.0
7 Pulse Sequence s2pul
8 Experiment 1D
9 Probe autox7991
10 Number of Scans 8
11 Receiver Gain 34
12 Relaxation Delay 1.0000
13 Pulse Width 5.8000
14 Acquisition Date 2018-05-26T07:59:23
15 Spectrometer Frequency 499.64
16 Spectral Width 8000.0
17 Lowest Frequency -1015.2
18 Nucleus 1H
19 Acquired Size 24000
20 Spectral Size 65536
B (m) 7.80
A (m) 7.49
C (m) 7.42
D (m) 5.99
E (dd) 4.98
F (m) 4.76
G (q) 4.28
H (m) 3.02
I (dddd) 2.49
J (m) 2.25
K (m) 1.90
L (t) 1.31
f1 (ppm)

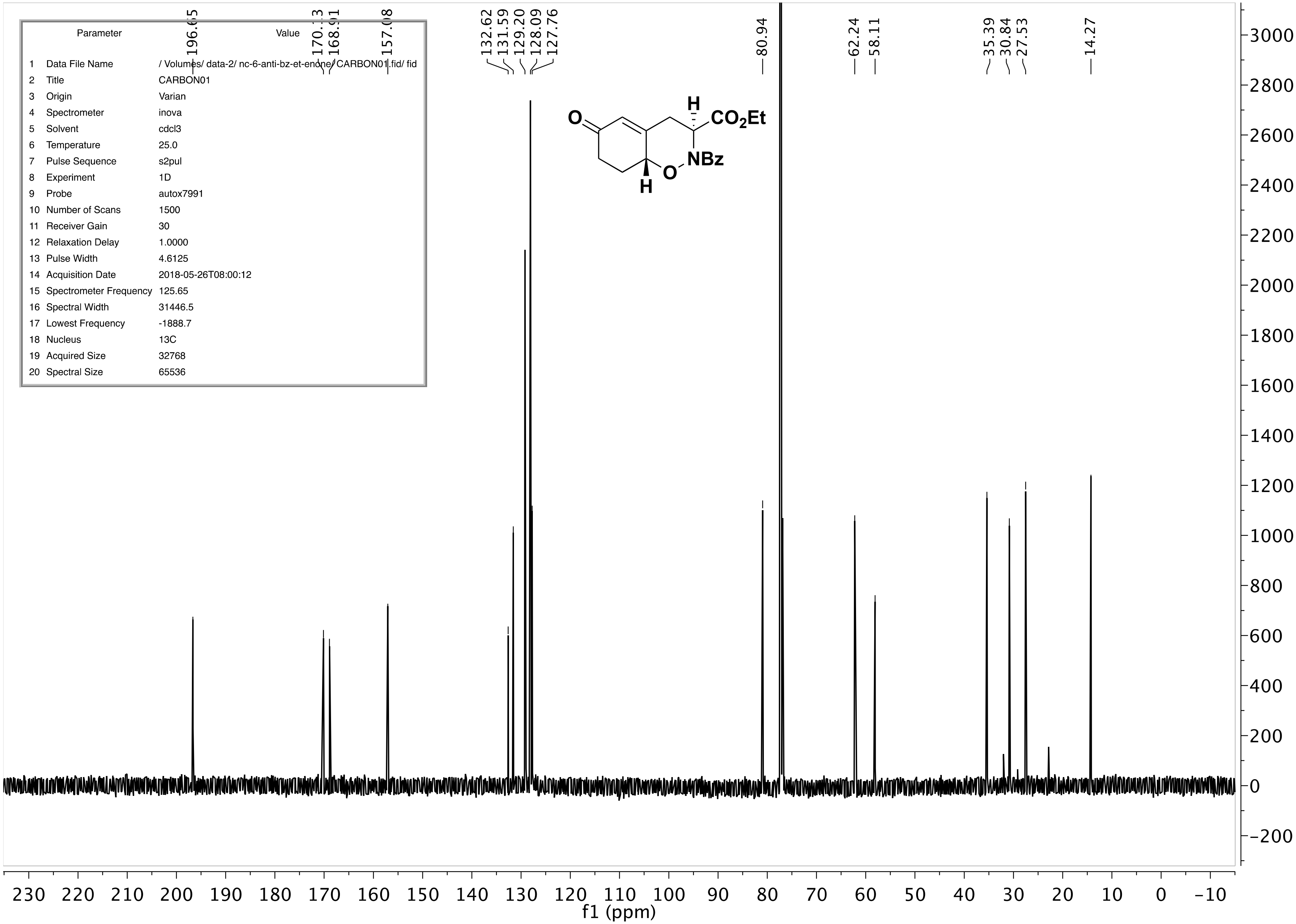

Parameter
Value
1 Data File Name / Volumes/ data-2/ nc-6-anti-bz-et-enone/ CARBON01.fid/ fid
2 Title CARBON01
3 Origin Varian
4 Spectrometer inova
5 Solvent cdcl3
6 Temperature 25.0
7 Pulse Sequence s2pul
8 Experiment 1D
9 Probe autox7991
10 Number of Scans 1500
11 Receiver Gain 30
12 Relaxation Delay 1.0000
13 Pulse Width 4.6125
14 Acquisition Date 2018-05-26T08:00:12
15 Spectrometer Frequency 125.65
16 Spectral Width 31446.5
17 Lowest Frequency -1888.7
18 Nucleus 13C
19 Acquired Size 32768
20 Spectral Size 65536
196.65
168.91
157.08
132.62
131.59
129.20
128.09
127.76
80.94
62.24
58.11
35.39
30.84
27.53
14.27
O
H
CO2Et
NBz
O
H
f1 (ppm)

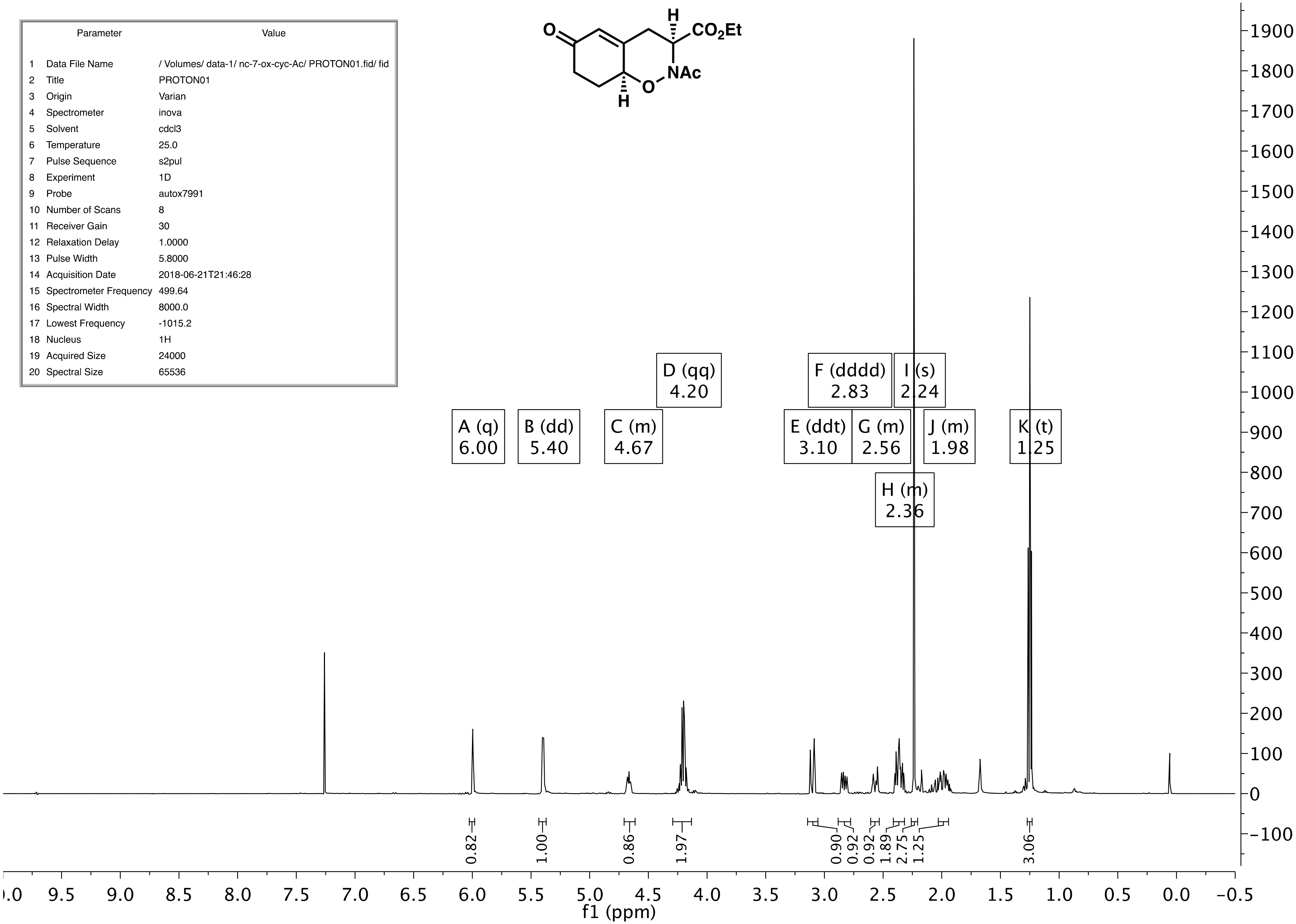

	Parameter	Value
1	Data File Name	/ Volumes/ data-1/ nc-7-ox-cyc-Ac/ PROTON01.fid/ fid
2	Title	PROTON01
3	Origin	Varian
4	Spectrometer	inova
5	Solvent	cdcl3
6	Temperature	25.0
7	Pulse Sequence	s2pul
8	Experiment	1D
9	Probe	autox7991
10	Number of Scans	8
11	Receiver Gain	30
12	Relaxation Delay	1.0000
13	Pulse Width	5.8000
14	Acquisition Date	2018-06-21T21:46:28
15	Spectrometer Frequency	499.64
16	Spectral Width	8000.0
17	Lowest Frequency	-1015.2
18	Nucleus	1H
19	Acquired Size	24000
20	Spectral Size	65536

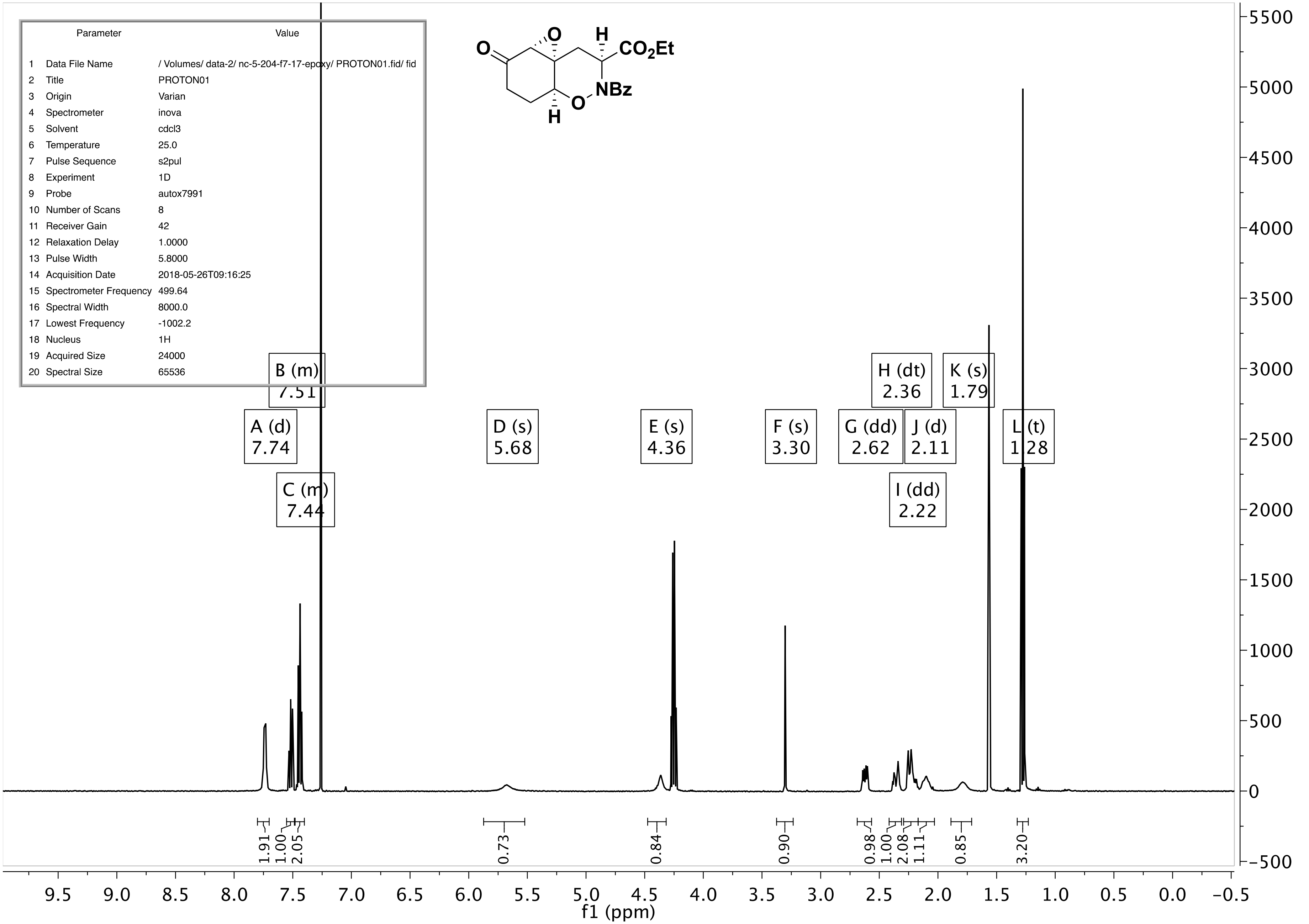
Parameter Value
1 Data File Name / Volumes/ data-2/ nc-5-204-f7-17-epoxy/ PROTON01.fid/ fid
2 Title PROTON01
3 Origin Varian
4 Spectrometer inova
5 Solvent cdcl3
6 Temperature 25.0
7 Pulse Sequence s2pul
8 Experiment 1D
9 Probe autox7991
10 Number of Scans 8
11 Receiver Gain 42
12 Relaxation Delay 1.0000
13 Pulse Width 5.8000
14 Acquisition Date 2018-05-26T09:16:25
15 Spectrometer Frequency 499.64
16 Spectral Width 8000.0
17 Lowest Frequency -1002.2
18 Nucleus 1H
19 Acquired Size 24000
20 Spectral Size 65536
O
O
H
CO_2Et
NBz
O
H
A (d) 7.74
B (m) 7.51
C (m) 7.44
D (s) 5.68
E (s) 4.36
F (s) 3.30
G (dd) 2.62
H (dt) 2.36
I (dd) 2.22
J (d) 2.11
K (s) 1.79
L (t) 1.28
1.91
1.00
2.05
0.73
0.84
0.90
0.98
1.00
2.08
1.11
0.85
3.20
f1 (ppm)

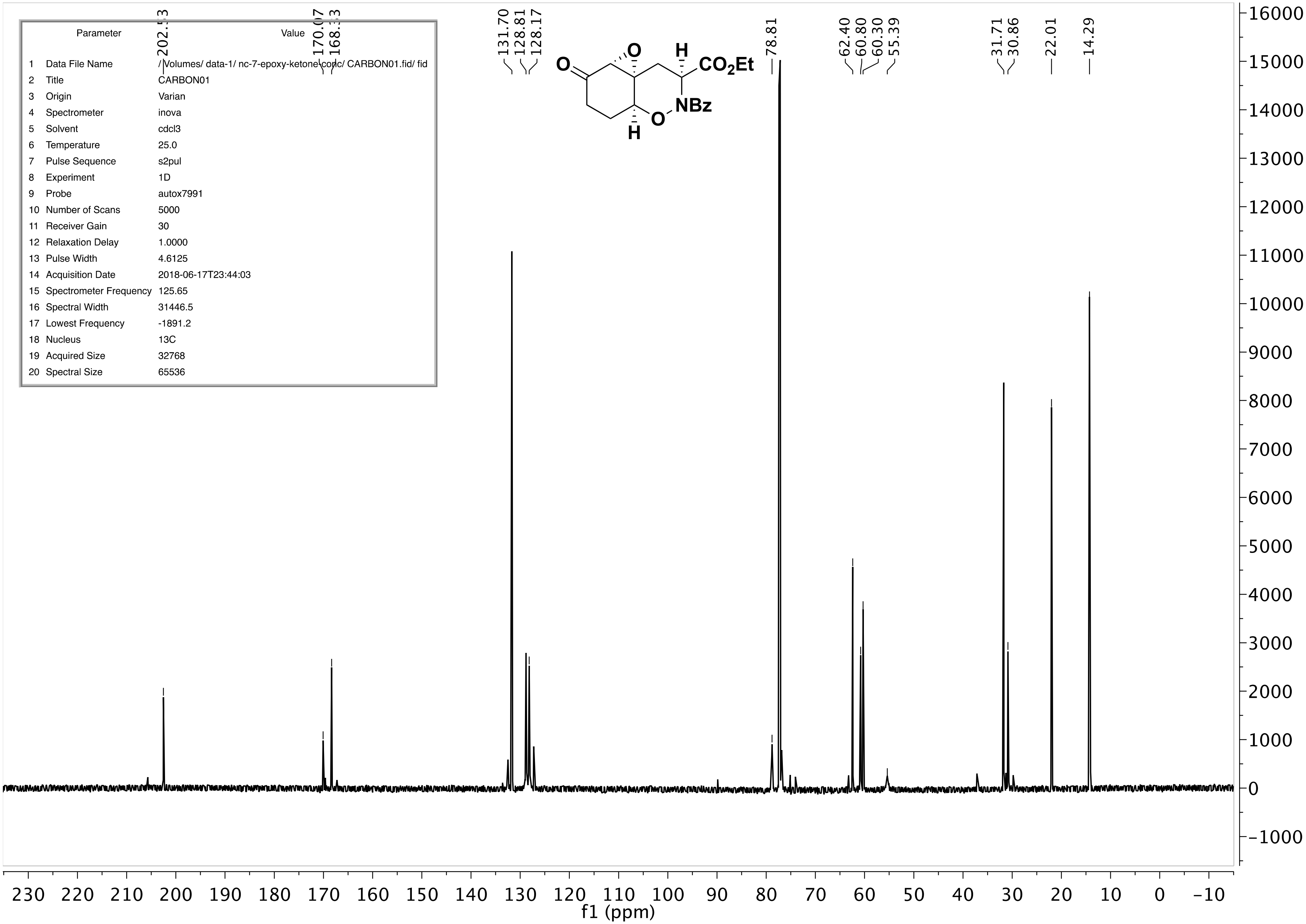

Parameter Value
1 Data File Name /Volumes/ data-1/ nc-7-epoxy-ketone conc/ CARBON01.fid/ fid
2 Title CARBON01
3 Origin Varian
4 Spectrometer inova
5 Solvent cdcl3
6 Temperature 25.0
7 Pulse Sequence s2pul
8 Experiment 1D
9 Probe autox7991
10 Number of Scans 5000
11 Receiver Gain 30
12 Relaxation Delay 1.0000
13 Pulse Width 4.6125
14 Acquisition Date 2018-06-17T23:44:03
15 Spectrometer Frequency 125.65
16 Spectral Width 31446.5
17 Lowest Frequency -1891.2
18 Nucleus 13C
19 Acquired Size 32768
20 Spectral Size 65536
170.07
168.33
131.70
128.81
128.17
78.81
62.40
60.80
60.30
55.39
31.71
30.86
22.01
14.29
O
H
CO_2Et
NBz
f1 (ppm)

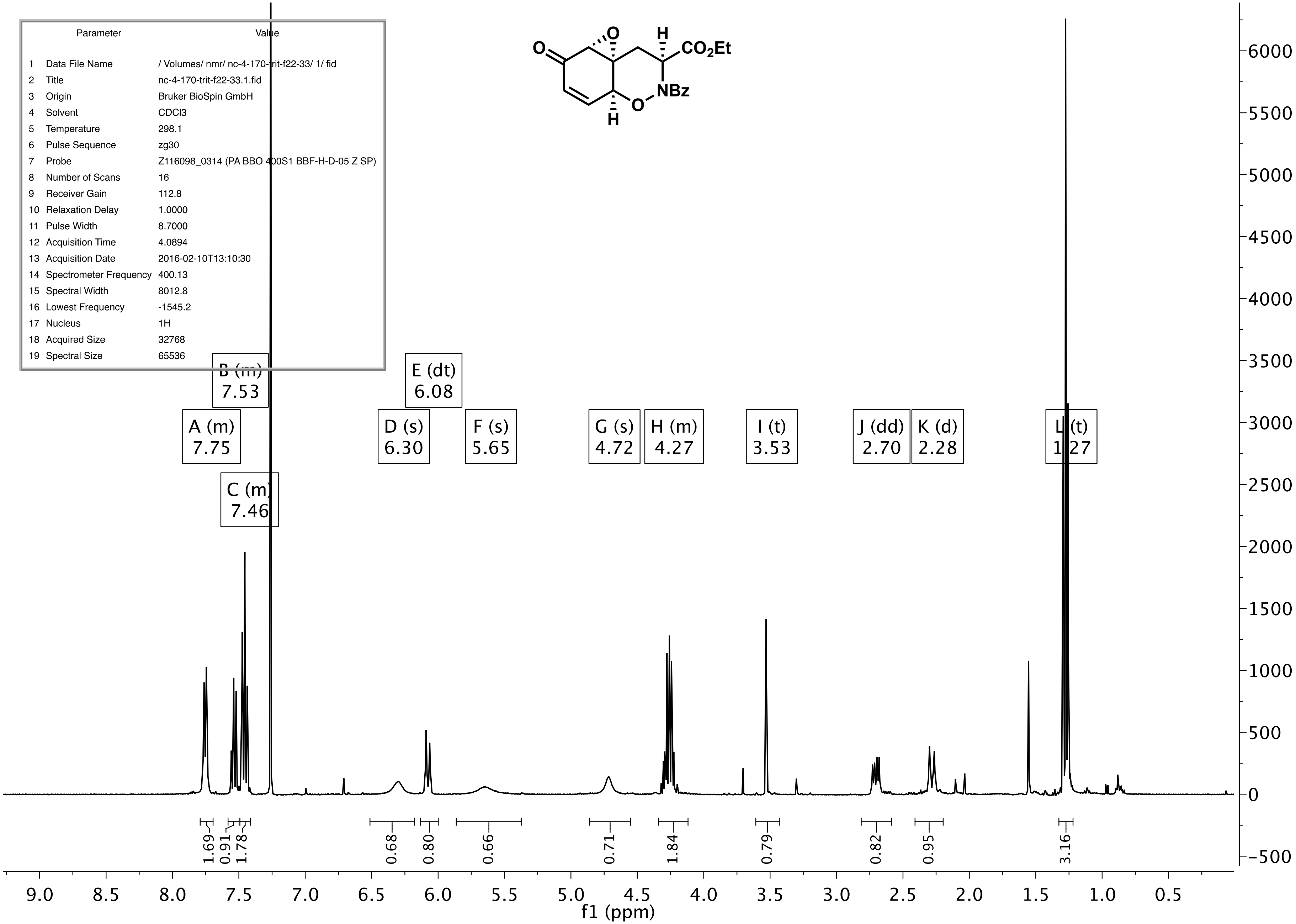
Parameter Value
1 Data File Name / Volumes/ nmr/ nc-4-170-trit-f22-33/ 1/ fid
2 Title nc-4-170-trit-f22-33.1.fid
3 Origin Bruker BioSpin GmbH
4 Solvent CDCl3
5 Temperature 298.1
6 Pulse Sequence zg30
7 Probe Z116098_0314 (PA BBO 400S1 BBF-H-D-05 Z SP)
8 Number of Scans 16
9 Receiver Gain 112.8
10 Relaxation Delay 1.0000
11 Pulse Width 8.7000
12 Acquisition Time 4.0894
13 Acquisition Date 2016-02-10T13:10:30
14 Spectrometer Frequency 400.13
15 Spectral Width 8012.8
16 Lowest Frequency -1545.2
17 Nucleus 1H
18 Acquired Size 32768
19 Spectral Size 65536
O
H
CO2Et
NBz
O
H
A (m) 7.75
B (m) 7.53
C (m) 7.46
D (s) 6.30
E (dt) 6.08
F (s) 5.65
G (s) 4.72
H (m) 4.27
I (t) 3.53
J (dd) 2.70
K (d) 2.28
L (t) 1.27
1.69
0.91
1.78
0.68
0.80
0.66
0.71
1.84
0.79
0.82
0.95
3.16
f1 (ppm)

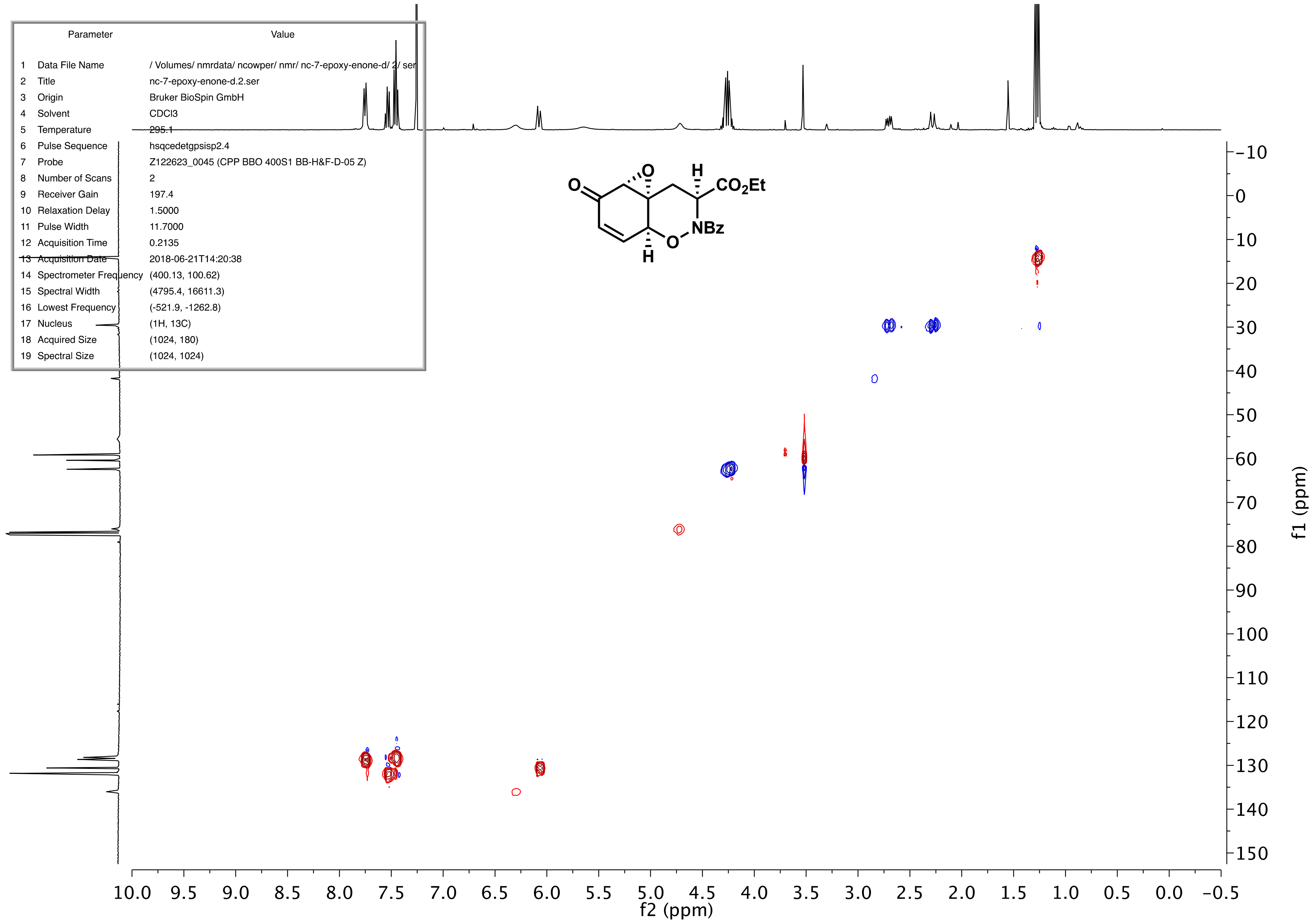

	Parameter	Value
1	Data File Name	/ Volumes/ nmrdata/ ncowper/ nmr/ nc-7-epoxy-enone-d/ 2/ ser
2	Title	nc-7-epoxy-enone-d.2.ser
3	Origin	Bruker BioSpin GmbH
4	Solvent	CDCl3
5	Temperature	295.1
6	Pulse Sequence	hsqcedetgpsisp2.4
7	Probe	Z122623_0045 (CPP BBO 400S1 BB-H&F-D-05 Z)
8	Number of Scans	2
9	Receiver Gain	197.4
10	Relaxation Delay	1.5000
11	Pulse Width	11.7000
12	Acquisition Time	0.2135
13	Acquisition Date	2018-06-21T14:20:38
14	Spectrometer Frequency	(400.13, 100.62)
15	Spectral Width	(4795.4, 16611.3)
16	Lowest Frequency	(-521.9, -1262.8)
17	Nucleus	(1H, 13C)
18	Acquired Size	(1024, 180)
19	Spectral Size	(1024, 1024)

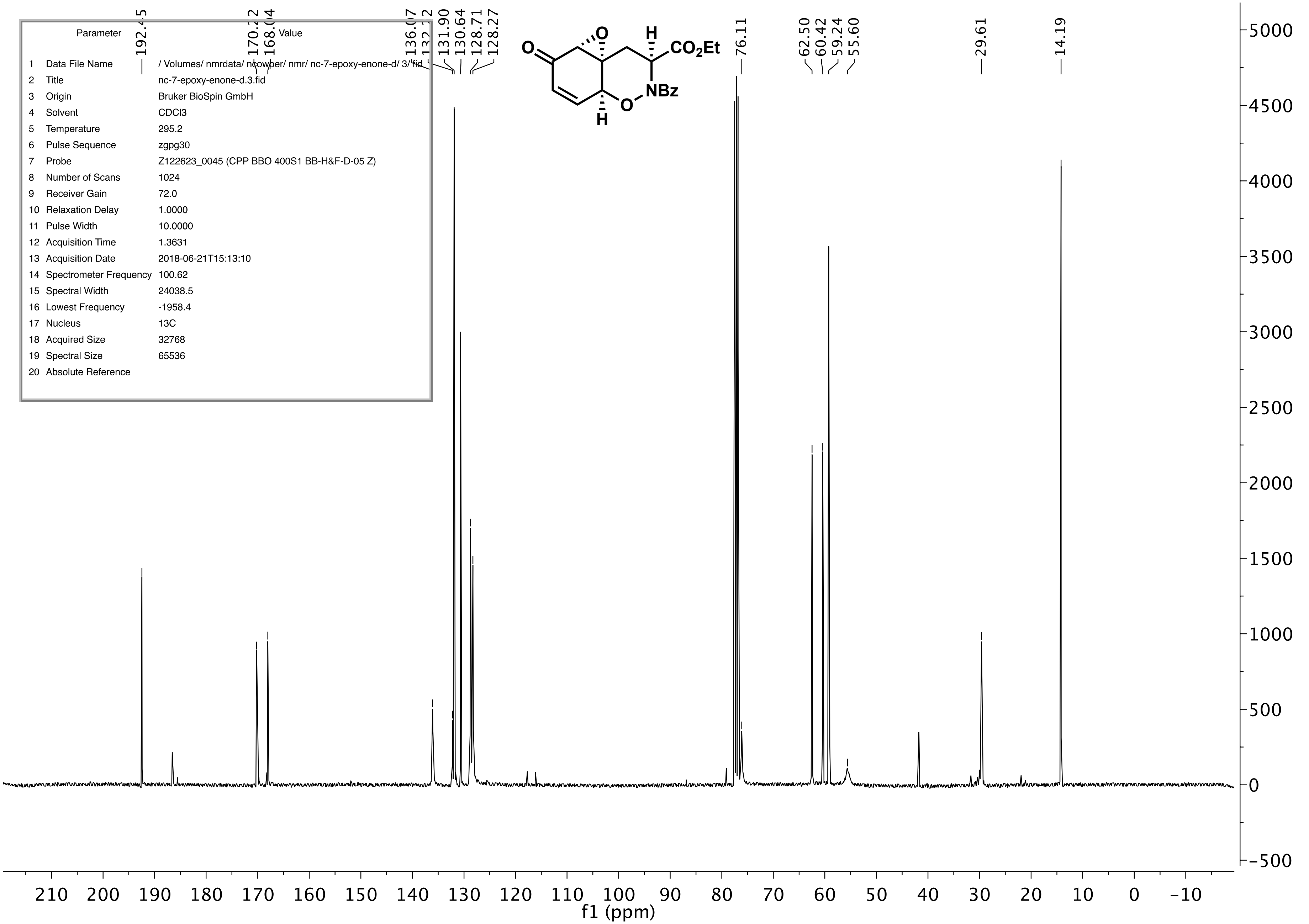

Parameter
Value
1 Data File Name / Volumes/ nmrdata/ ncowper/ nmr/ nc-7-epoxy-enone-d/ 3/ fid
2 Title nc-7-epoxy-enone-d.3.fid
3 Origin Bruker BioSpin GmbH
4 Solvent CDCl3
5 Temperature 295.2
6 Pulse Sequence zgpg30
7 Probe Z122623_0045 (CPP BBO 400S1 BB-H&F-D-05 Z)
8 Number of Scans 1024
9 Receiver Gain 72.0
10 Relaxation Delay 1.0000
11 Pulse Width 10.0000
12 Acquisition Time 1.3631
13 Acquisition Date 2018-06-21T15:13:10
14 Spectrometer Frequency 100.62
15 Spectral Width 24038.5
16 Lowest Frequency -1958.4
17 Nucleus 13C
18 Acquired Size 32768
19 Spectral Size 65536
20 Absolute Reference
168.04
136.07
131.90
130.64
128.71
128.27
76.11
62.50
60.42
59.24
55.60
29.61
14.19
O
H
CO2Et
NBz
O
H
O
5000
4500
4000
3500
3000
2500
2000
1500
1000
500
0
−500
210 200 190 180 170 160 150 140 130 120 110 100 90 80 70 60 50 40 30 20 10 0 −10
f1 (ppm)

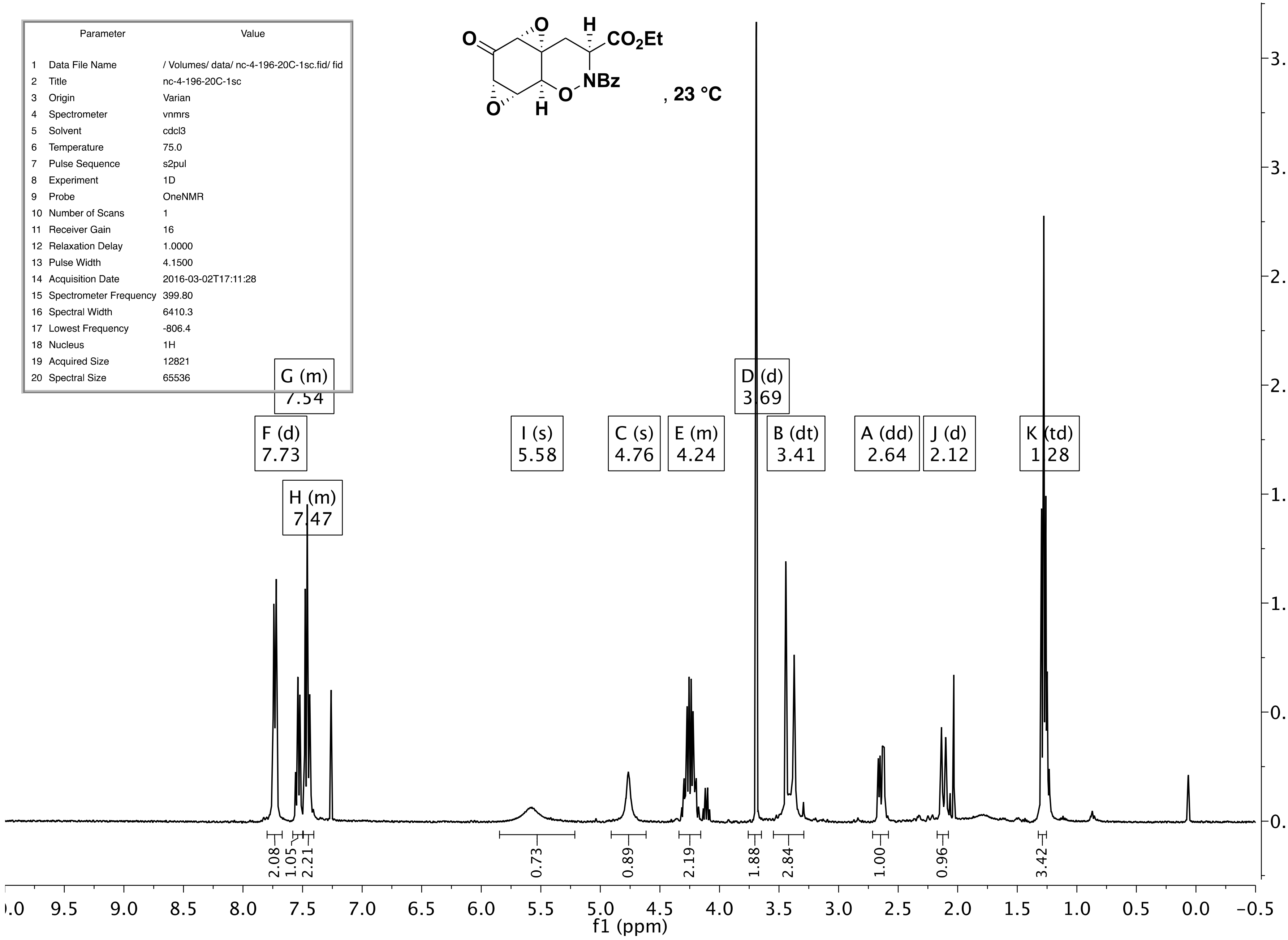

Parameter		Value
1	Data File Name	/ Volumes/ data/ nc-4-196-20C-1sc.fid/ fid
2	Title	nc-4-196-20C-1sc
3	Origin	Varian
4	Spectrometer	vnmrs
5	Solvent	cdcl3
6	Temperature	75.0
7	Pulse Sequence	s2pul
8	Experiment	1D
9	Probe	OneNMR
10	Number of Scans	1
11	Receiver Gain	16
12	Relaxation Delay	1.0000
13	Pulse Width	4.1500
14	Acquisition Date	2016-03-02T17:11:28
15	Spectrometer Frequency	399.80
16	Spectral Width	6410.3
17	Lowest Frequency	-806.4
18	Nucleus	1H
19	Acquired Size	12821
20	Spectral Size	65536

	Parameter	Value
1	Data File Name	/ Volumes/ data/ nc-4-196-46C-shimmed-1sc-10s-at.fid/ fid
2	Title	nc-4-196-46C-shimmed-1sc-10s-at
3	Origin	Varian
4	Spectrometer	vnmrs
5	Solvent	cdcl3
6	Temperature	46.0
7	Pulse Sequence	s2pul
8	Experiment	1D
9	Probe	OneNMR
10	Number of Scans	1
11	Receiver Gain	48
12	Relaxation Delay	1.0000
13	Pulse Width	4.1500
14	Acquisition Date	2016-03-02T17:38:56
15	Spectrometer Frequency	399.80
16	Spectral Width	6410.3
17	Lowest Frequency	-806.4
18	Nucleus	1H
19	Acquired Size	64103
20	Spectral Size	131072

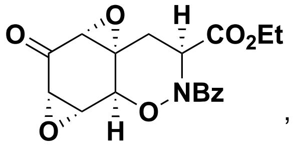

, 46 °C

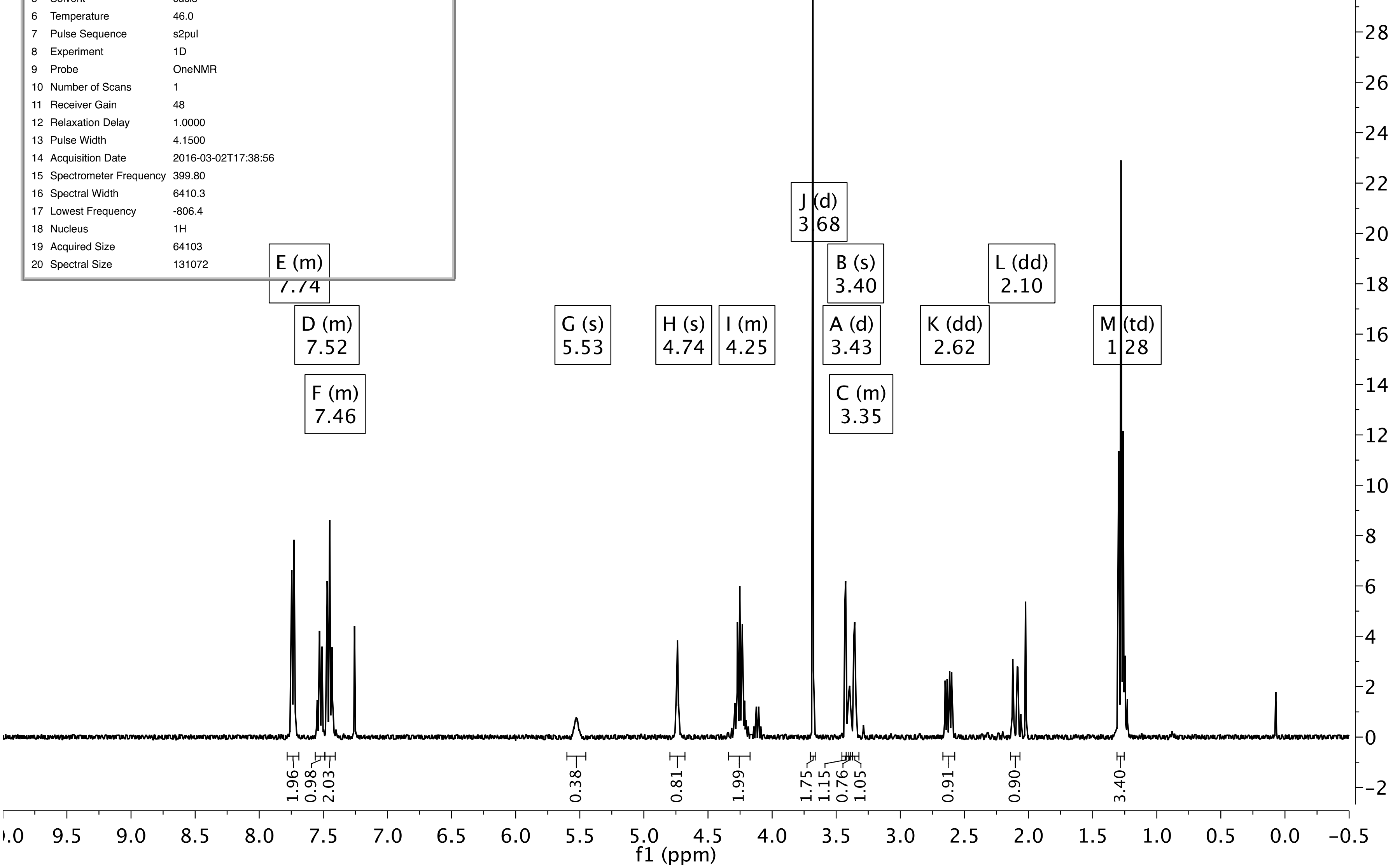

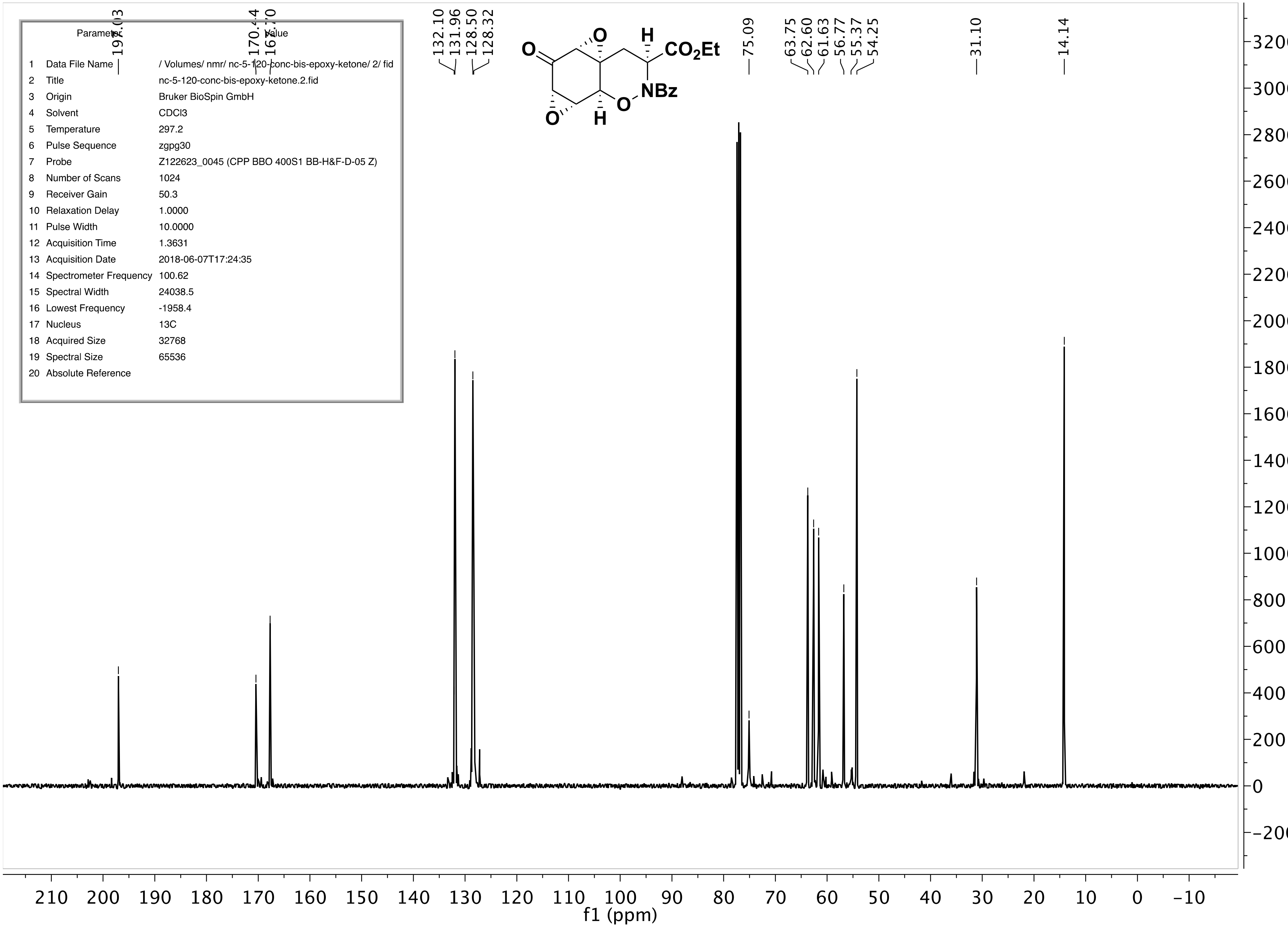
Parameter
Value
1 Data File Name / Volumes/ nmr/ nc-5-120-conc-bis-epoxy-ketone/ 2/ fid
2 Title nc-5-120-conc-bis-epoxy-ketone.2.fid
3 Origin Bruker BioSpin GmbH
4 Solvent CDCl3
5 Temperature 297.2
6 Pulse Sequence zgpg30
7 Probe Z122623_0045 (CPP BBO 400S1 BB-H&F-D-05 Z)
8 Number of Scans 1024
9 Receiver Gain 50.3
10 Relaxation Delay 1.0000
11 Pulse Width 10.0000
12 Acquisition Time 1.3631
13 Acquisition Date 2018-06-07T17:24:35
14 Spectrometer Frequency 100.62
15 Spectral Width 24038.5
16 Lowest Frequency -1958.4
17 Nucleus 13C
18 Acquired Size 32768
19 Spectral Size 65536
20 Absolute Reference
132.10
131.96
128.50
128.32
75.09
63.75
62.60
61.63
56.77
55.37
54.25
31.10
14.14
O
O
H
CO2Et
NBz
O
O
H
f1 (ppm)

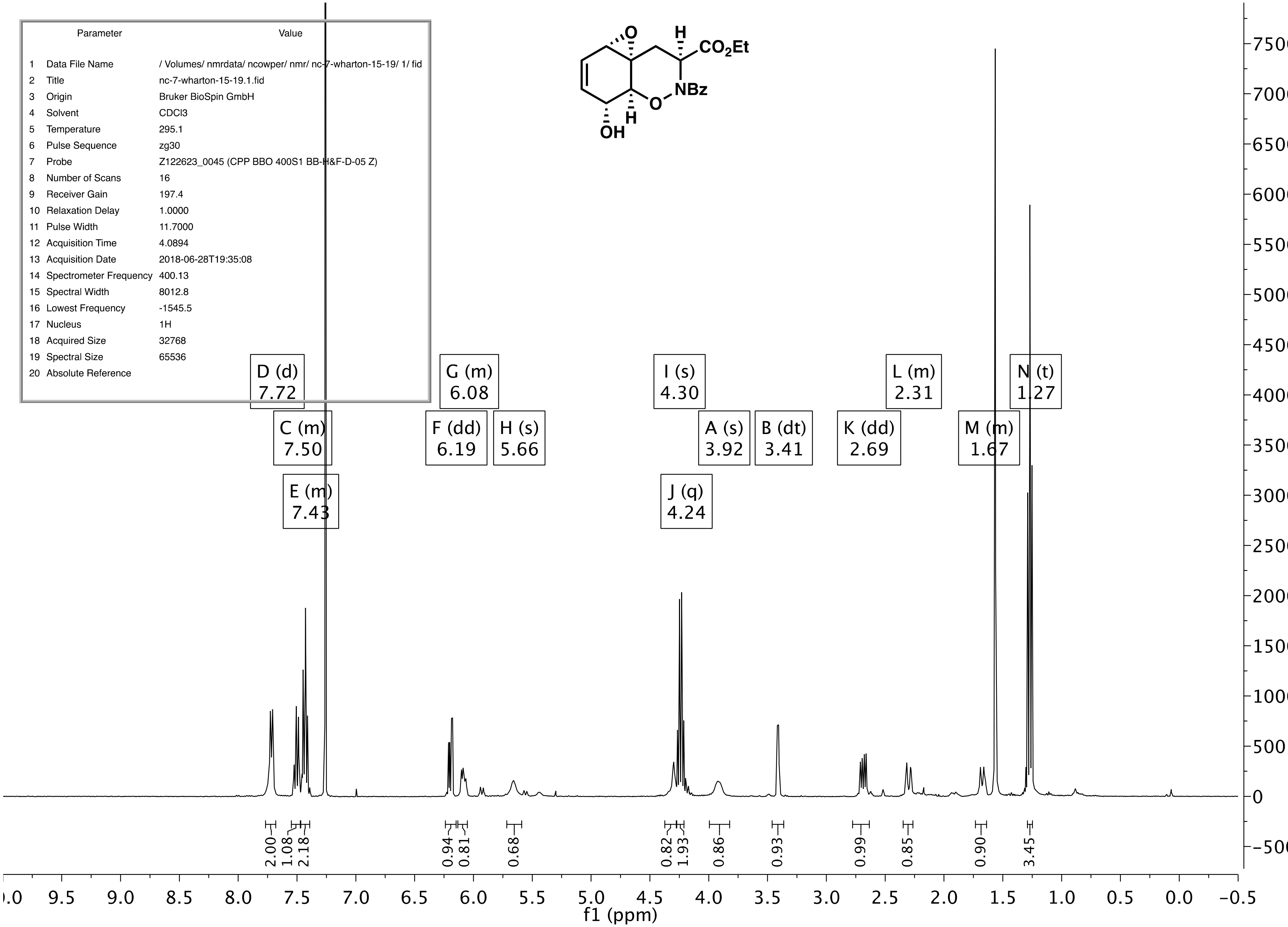
Parameter Value
1 Data File Name / Volumes/ nmrdata/ ncowper/ nmr/ nc-7-wharton-15-19/ 1/ fid
2 Title nc-7-wharton-15-19.1.fid
3 Origin Bruker BioSpin GmbH
4 Solvent CDCl3
5 Temperature 295.1
6 Pulse Sequence zg30
7 Probe Z122623_0045 (CPP BBO 400S1 BB-H&F-D-05 Z)
8 Number of Scans 16
9 Receiver Gain 197.4
10 Relaxation Delay 1.0000
11 Pulse Width 11.7000
12 Acquisition Time 4.0894
13 Acquisition Date 2018-06-28T19:35:08
14 Spectrometer Frequency 400.13
15 Spectral Width 8012.8
16 Lowest Frequency -1545.5
17 Nucleus 1H
18 Acquired Size 32768
19 Spectral Size 65536
20 Absolute Reference
O
H
CO2Et
NBz
O
H
OH
D (d) 7.72
C (m) 7.50
E (m) 7.43
G (m) 6.08
F (dd) 6.19
H (s) 5.66
I (s) 4.30
J (q) 4.24
A (s) 3.92
B (dt) 3.41
K (dd) 2.69
L (m) 2.31
M (m) 1.67
N (t) 1.27
2.00 1.08 2.18 0.94 0.81 0.68 0.82 1.93 0.86 0.93 0.99 0.85 0.90 3.45
f1 (ppm)

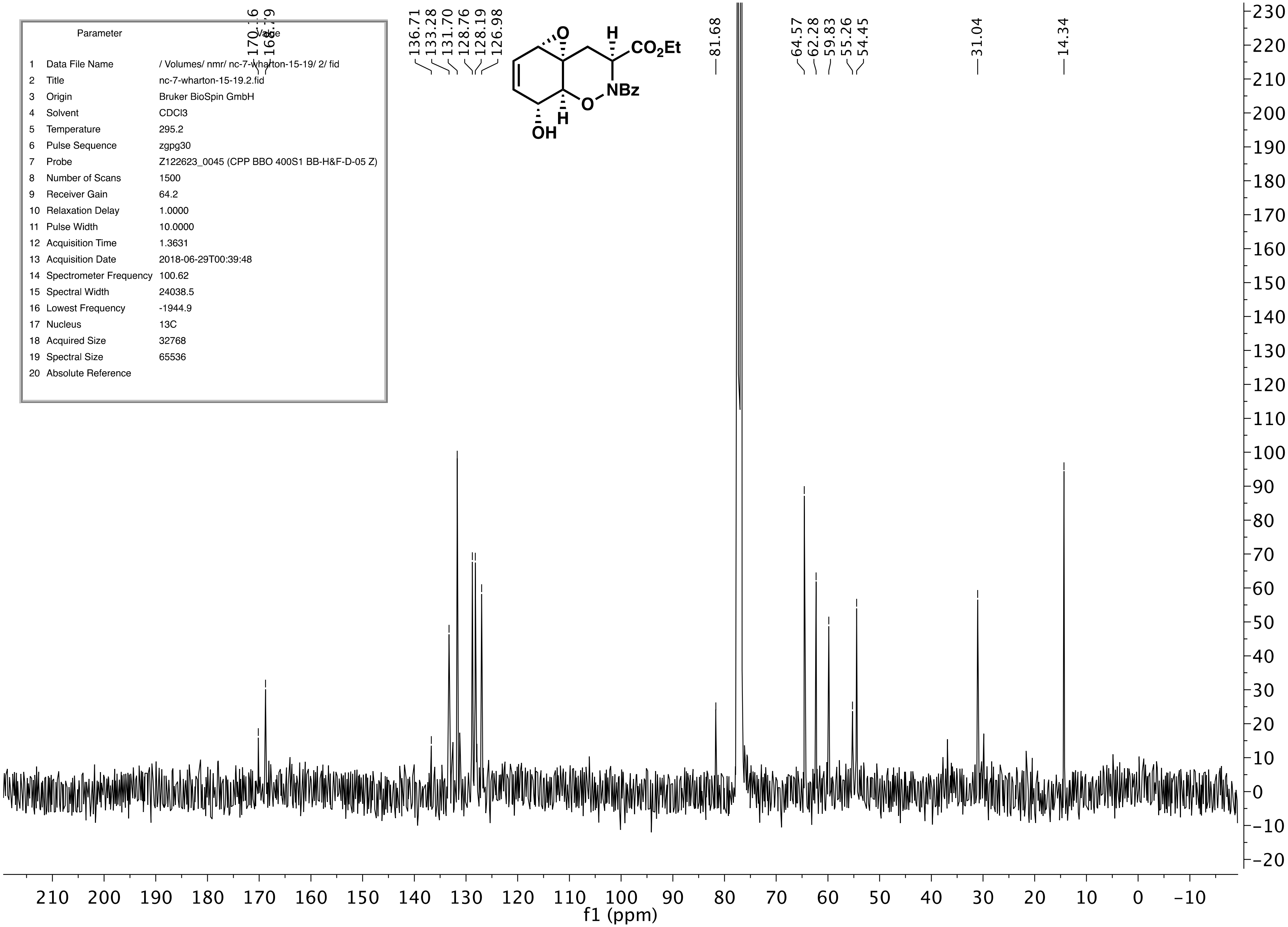

	Parameter	Value
1	Data File Name	/ Volumes/ nmr/ nc-7-wharton-15-19/ 2/ fid
2	Title	nc-7-wharton-15-19.2.fid
3	Origin	Bruker BioSpin GmbH
4	Solvent	CDCl3
5	Temperature	295.2
6	Pulse Sequence	zgpg30
7	Probe	Z122623_0045 (CPP BBO 400S1 BB-H&F-D-05 Z)
8	Number of Scans	1500
9	Receiver Gain	64.2
10	Relaxation Delay	1.0000
11	Pulse Width	10.0000
12	Acquisition Time	1.3631
13	Acquisition Date	2018-06-29T00:39:48
14	Spectrometer Frequency	100.62
15	Spectral Width	24038.5
16	Lowest Frequency	-1944.9
17	Nucleus	13C
18	Acquired Size	32768
19	Spectral Size	65536
20	Absolute Reference	

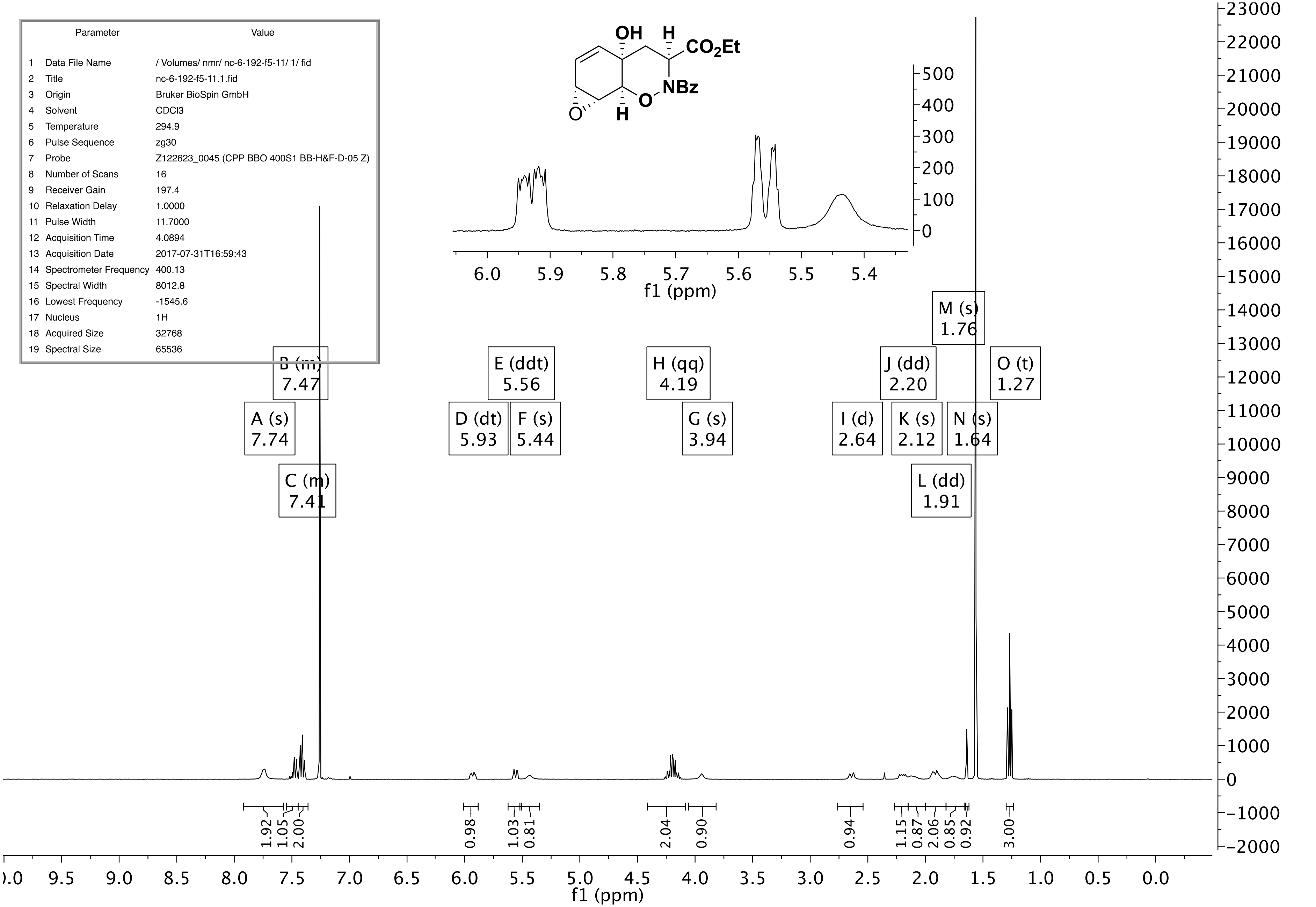

Parameter Value
1 Data File Name / Volumes/ nmr/ nc-6-192-f5-11/ 1/ fid
2 Title nc-6-192-f5-11.1.fid
3 Origin Bruker BioSpin GmbH
4 Solvent CDCl3
5 Temperature 294.9
6 Pulse Sequence zg30
7 Probe Z122623_0045 (CPP BBO 400S1 BB-H&F-D-05 Z)
8 Number of Scans 16
9 Receiver Gain 197.4
10 Relaxation Delay 1.0000
11 Pulse Width 11.7000
12 Acquisition Time 4.0894
13 Acquisition Date 2017-07-31T16:59:43
14 Spectrometer Frequency 400.13
15 Spectral Width 8012.8
16 Lowest Frequency -1545.6
17 Nucleus 1H
18 Acquired Size 32768
19 Spectral Size 65536
OH
H
CO_2Et
NBz
O
O
H
A (s) 7.74
B (m) 7.47
C (m) 7.41
D (dt) 5.93
E (ddt) 5.56
F (s) 5.44
H (qq) 4.19
G (s) 3.94
I (d) 2.64
J (dd) 2.20
K (s) 2.12
L (dd) 1.91
M (s) 1.76
N (s) 1.64
O (t) 1.27
1.92 1.05 2.00 0.98 1.03 0.81 2.04 0.90 0.94 1.15 0.87 2.06 0.85 0.92 3.00
f1 (ppm)

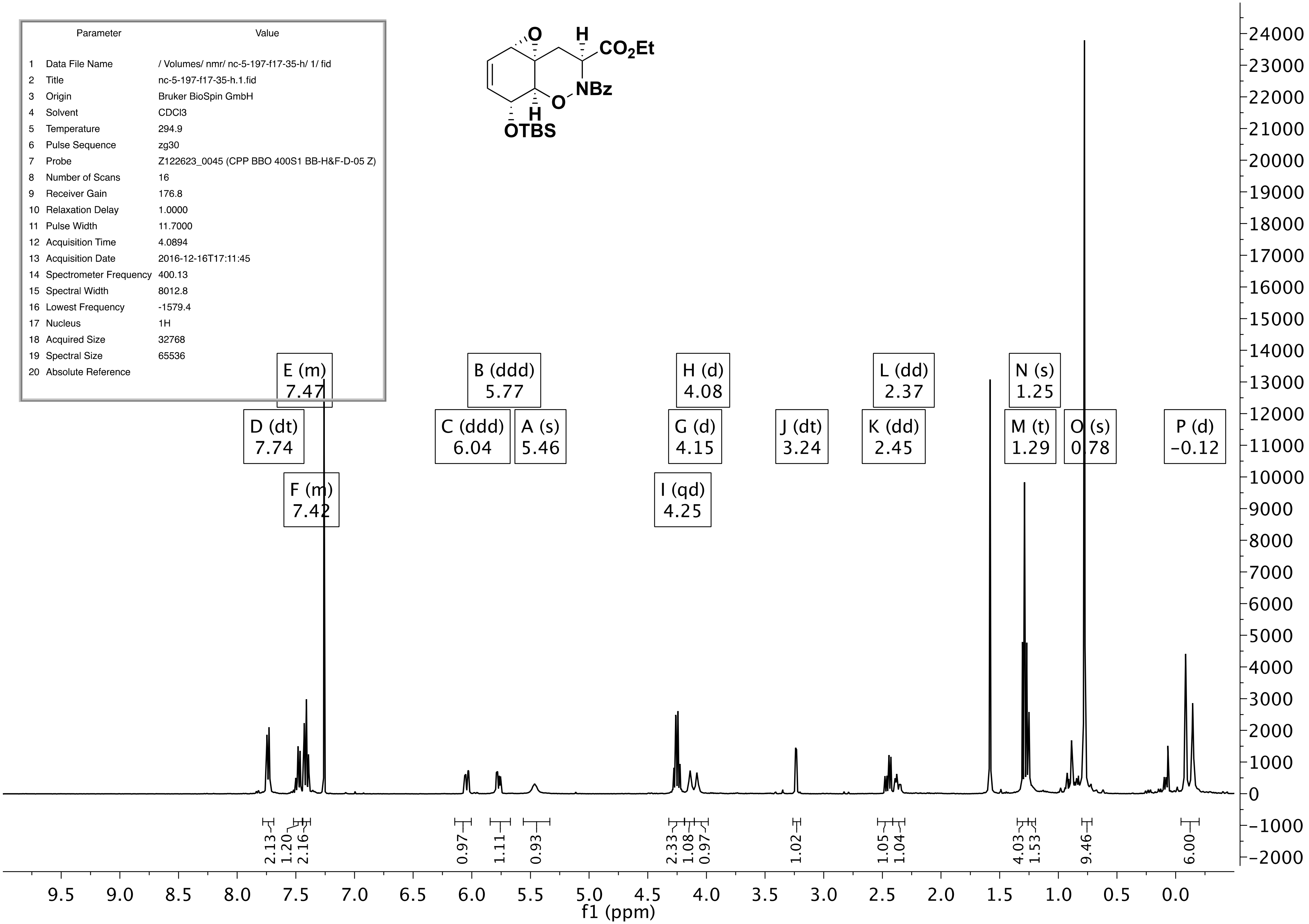
Parameter
Value
1 Data File Name / Volumes/ nmr/ nc-5-197-f17-35-h/ 1/ fid
2 Title nc-5-197-f17-35-h.1.fid
3 Origin Bruker BioSpin GmbH
4 Solvent CDCl3
5 Temperature 294.9
6 Pulse Sequence zg30
7 Probe Z122623_0045 (CPP BBO 400S1 BB-H&F-D-05 Z)
8 Number of Scans 16
9 Receiver Gain 176.8
10 Relaxation Delay 1.0000
11 Pulse Width 11.7000
12 Acquisition Time 4.0894
13 Acquisition Date 2016-12-16T17:11:45
14 Spectrometer Frequency 400.13
15 Spectral Width 8012.8
16 Lowest Frequency -1579.4
17 Nucleus 1H
18 Acquired Size 32768
19 Spectral Size 65536
20 Absolute Reference
O
H
CO2Et
NBz
O
H
OTBS
E (m) 7.47
D (dt) 7.74
F (m) 7.42
B (ddd) 5.77
C (ddd) 6.04
A (s) 5.46
H (d) 4.08
G (d) 4.15
I (qd) 4.25
J (dt) 3.24
L (dd) 2.37
K (dd) 2.45
N (s) 1.25
M (t) 1.29
O (s) 0.78
P (d) -0.12
2.13
1.20
2.16
0.97
1.11
0.93
2.33
1.08
0.97
1.02
1.05
1.04
4.03
1.53
9.46
6.00
9.5 9.0 8.5 8.0 7.5 7.0 6.5 6.0 5.5 5.0 4.5 4.0 3.5 3.0 2.5 2.0 1.5 1.0 0.5 0.0
f1 (ppm)
24000 23000 22000 21000 20000 19000 18000 17000 16000 15000 14000 13000 12000 11000 10000 9000 8000 7000 6000 5000 4000 3000 2000 1000 0 -1000 -2000

Parameter		Value
1	Data File Name	/ Volumes/ nmr/ nc-5-197-f17-35/ 1/ fid
2	Title	nc-5-197-f17-35.1.fid
3	Origin	Bruker BioSpin GmbH
4	Solvent	CDCl3
5	Temperature	294.9
6	Pulse Sequence	zgpg30
7	Probe	Z122623_0045 (CPP BBO 400S1 BB-H&F-D-05 Z)
8	Number of Scans	1024
9	Receiver Gain	72.0
10	Relaxation Delay	2.0000
11	Pulse Width	10.0000
12	Acquisition Time	1.3631
13	Acquisition Date	2016-12-15T16:36:28
14	Spectrometer Frequency	100.62
15	Spectral Width	24038.5
16	Lowest Frequency	-1932.3
17	Nucleus	13C
18	Acquired Size	32768
19	Spectral Size	65536
20	Absolute Reference	

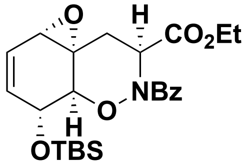

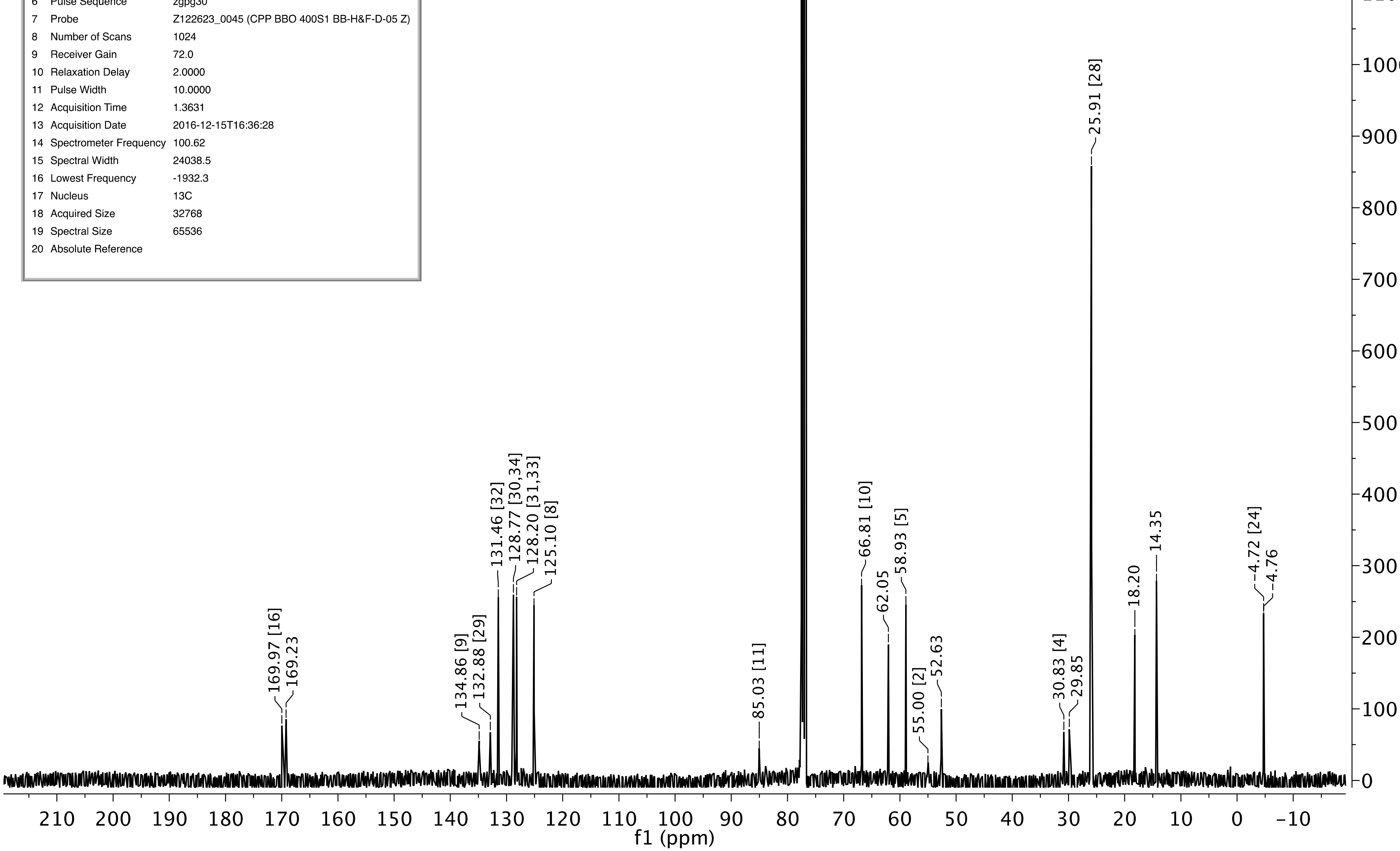

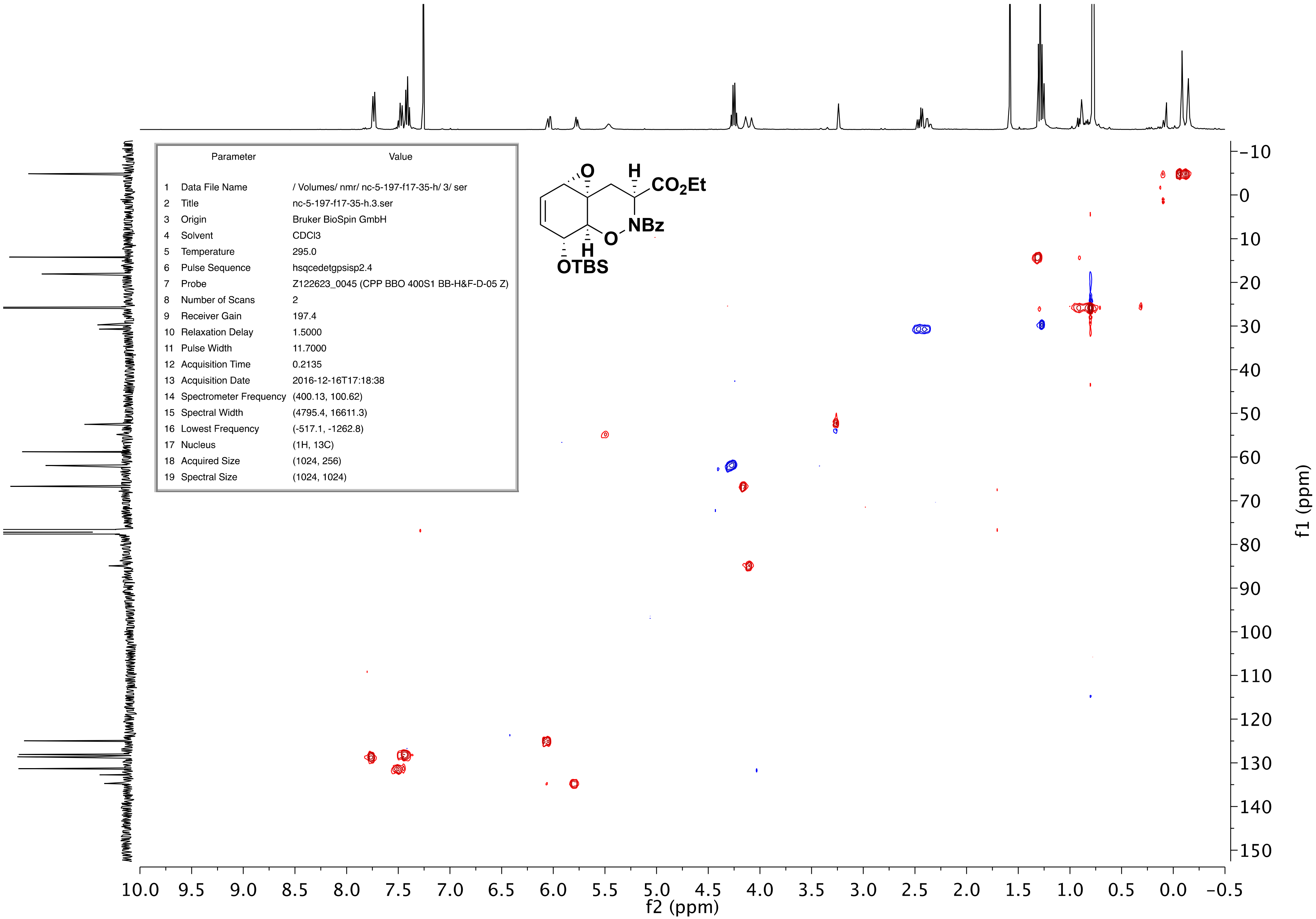

	Parameter	Value
1	Data File Name	/ Volumes/ nmr/ nc-5-197-f17-35-h/ 3/ ser
2	Title	nc-5-197-f17-35-h.3.ser
3	Origin	Bruker BioSpin GmbH
4	Solvent	CDCl3
5	Temperature	295.0
6	Pulse Sequence	hsqcedetgpsisp2.4
7	Probe	Z122623_0045 (CPP BBO 400S1 BB-H&F-D-05 Z)
8	Number of Scans	2
9	Receiver Gain	197.4
10	Relaxation Delay	1.5000
11	Pulse Width	11.7000
12	Acquisition Time	0.2135
13	Acquisition Date	2016-12-16T17:18:38
14	Spectrometer Frequency	(400.13, 100.62)
15	Spectral Width	(4795.4, 16611.3)
16	Lowest Frequency	(-517.1, -1262.8)
17	Nucleus	(1H, 13C)
18	Acquired Size	(1024, 256)
19	Spectral Size	(1024, 1024)

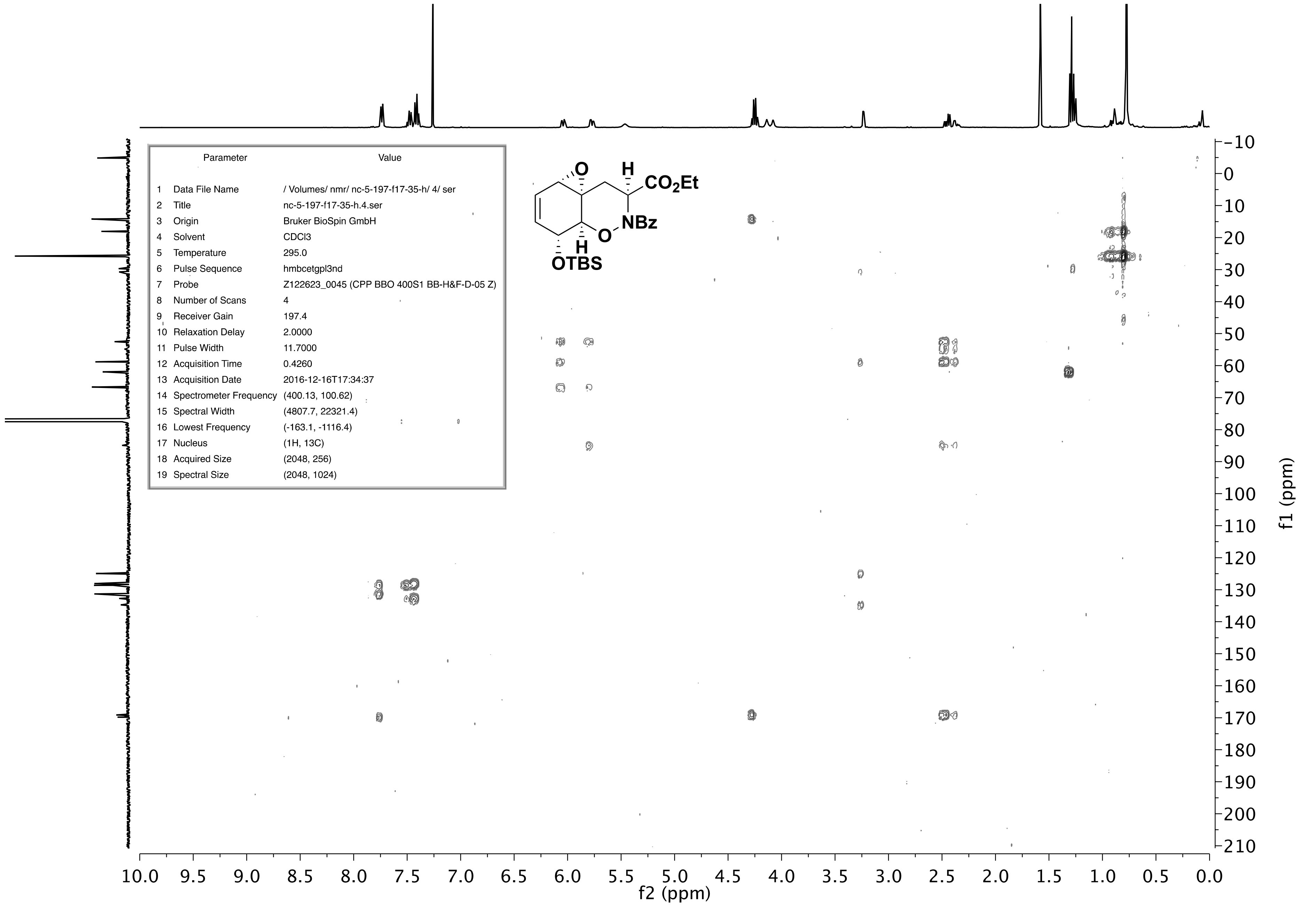

	Parameter	Value
1	Data File Name	/ Volumes/ nmr/ nc-5-197-f17-35-h/ 4/ ser
2	Title	nc-5-197-f17-35-h.4.ser
3	Origin	Bruker BioSpin GmbH
4	Solvent	CDCl3
5	Temperature	295.0
6	Pulse Sequence	hmbcetgpl3nd
7	Probe	Z122623_0045 (CPP BBO 400S1 BB-H&F-D-05 Z)
8	Number of Scans	4
9	Receiver Gain	197.4
10	Relaxation Delay	2.0000
11	Pulse Width	11.7000
12	Acquisition Time	0.4260
13	Acquisition Date	2016-12-16T17:34:37
14	Spectrometer Frequency	(400.13, 100.62)
15	Spectral Width	(4807.7, 22321.4)
16	Lowest Frequency	(-163.1, -1116.4)
17	Nucleus	(1H, 13C)
18	Acquired Size	(2048, 256)
19	Spectral Size	(2048, 1024)

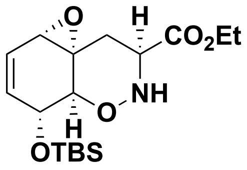

	Parameter	Value
1	Data File Name	/ Volumes/ nmrdata/ ncowper/ nmr/ nc-5-198-f9-14-h/ 1/ fid
2	Title	nc-5-198-f9-14-h.1.fid
3	Origin	Bruker BioSpin GmbH
4	Solvent	CDCl3
5	Temperature	294.9
6	Pulse Sequence	zg30
7	Probe	Z122623_0045 (CPP BBO 400S1 BB-H&F-D-05 Z)
8	Number of Scans	16
9	Receiver Gain	197.4
10	Relaxation Delay	1.0000
11	Pulse Width	11.7000
12	Acquisition Time	4.0894
13	Acquisition Date	2016-12-19T02:26:37
14	Spectrometer Frequency	400.13
15	Spectral Width	8012.8
16	Lowest Frequency	-1545.3
17	Nucleus	1H
18	Acquired Size	32768
19	Spectral Size	65536
20	Absolute Reference	

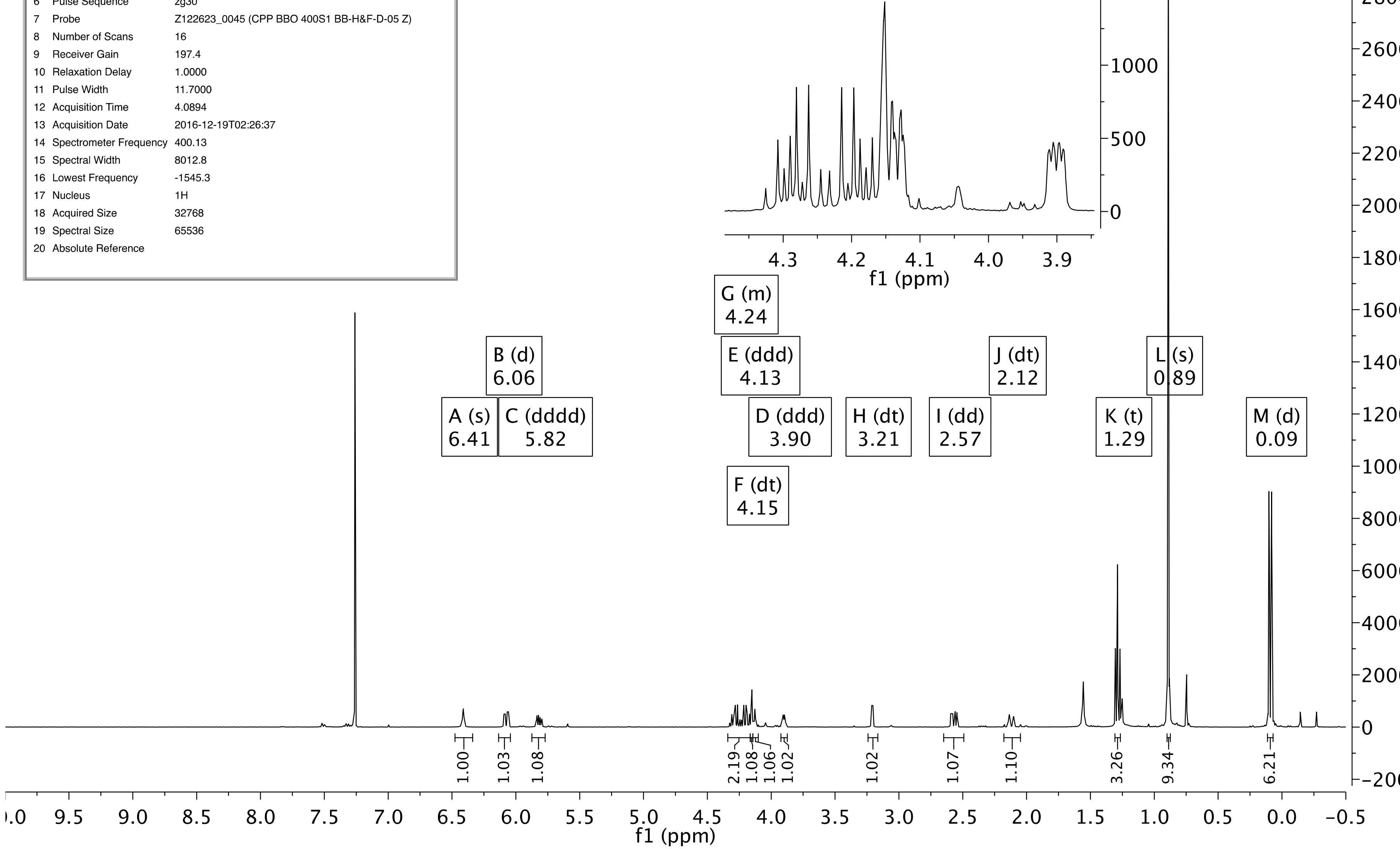

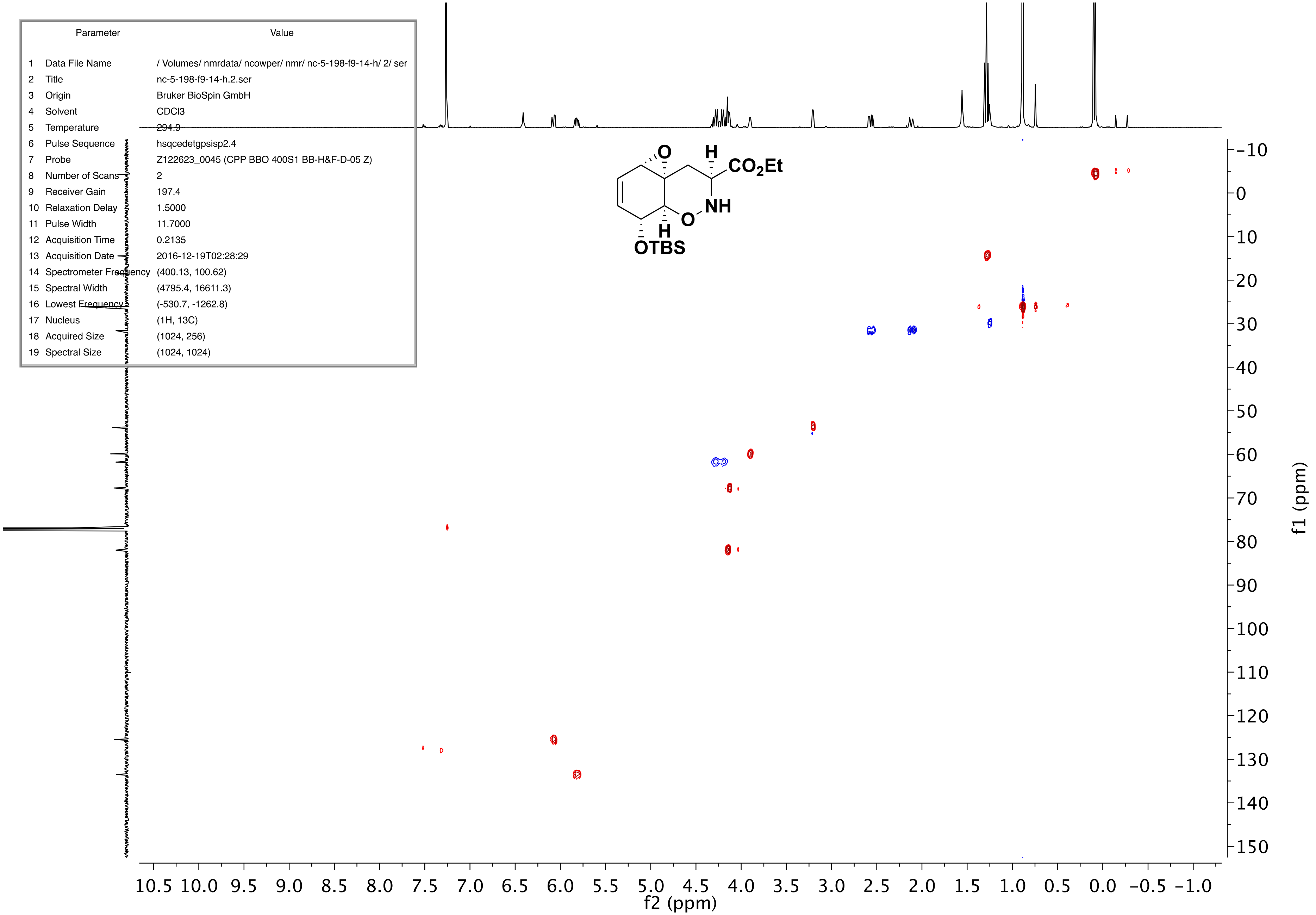

	Parameter	Value
1	Data File Name	/ Volumes/ nmrdata/ ncowper/ nmr/ nc-5-198-f9-14-h/ 2/ ser
2	Title	nc-5-198-f9-14-h.2.ser
3	Origin	Bruker BioSpin GmbH
4	Solvent	CDCl3
5	Temperature	294.9
6	Pulse Sequence	hsqcedetgpsisp2.4
7	Probe	Z122623_0045 (CPP BBO 400S1 BB-H&F-D-05 Z)
8	Number of Scans	2
9	Receiver Gain	197.4
10	Relaxation Delay	1.5000
11	Pulse Width	11.7000
12	Acquisition Time	0.2135
13	Acquisition Date	2016-12-19T02:28:29
14	Spectrometer Frequency	(400.13, 100.62)
15	Spectral Width	(4795.4, 16611.3)
16	Lowest Frequency	(-530.7, -1262.8)
17	Nucleus	(1H, 13C)
18	Acquired Size	(1024, 256)
19	Spectral Size	(1024, 1024)

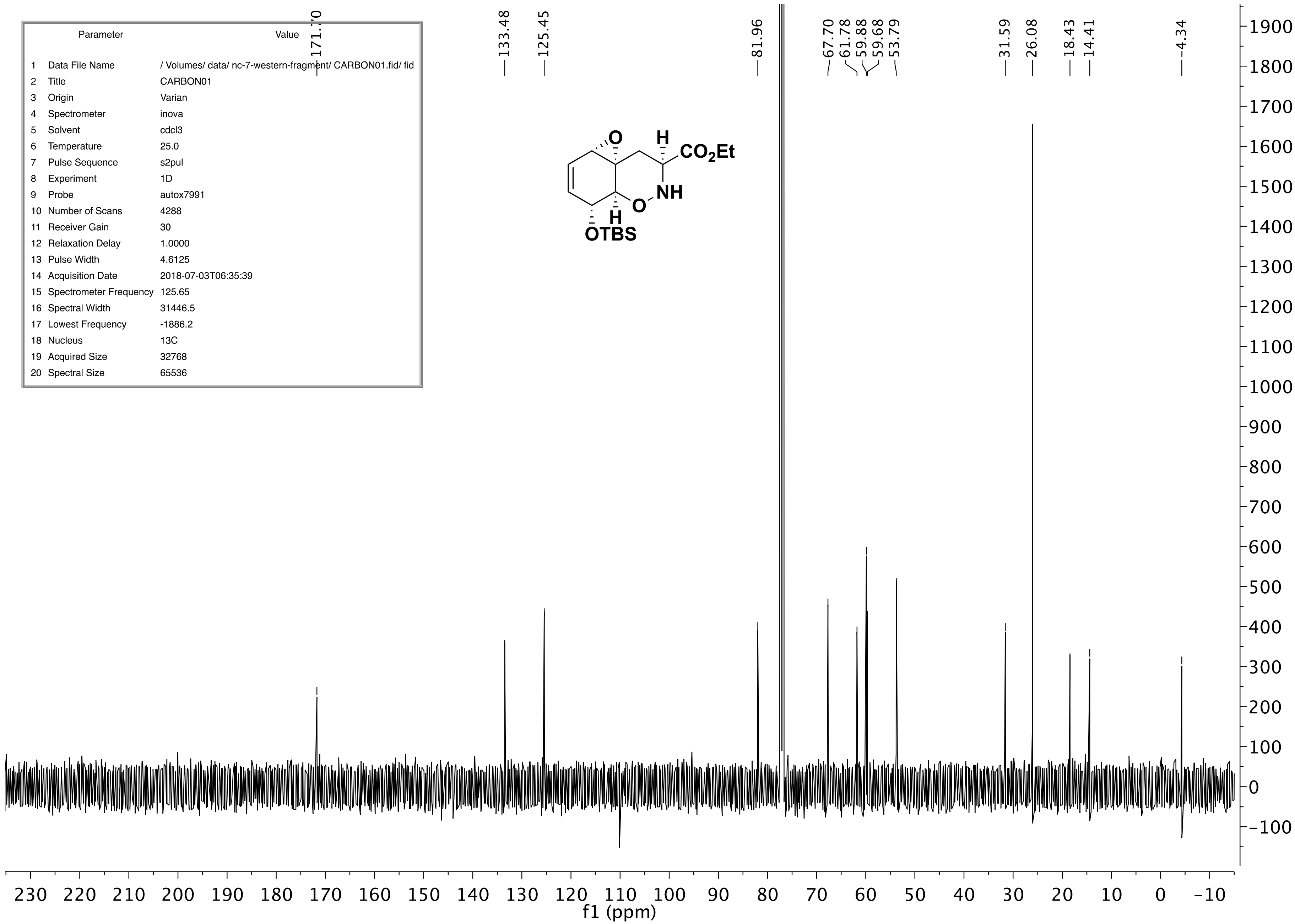

Parameter		Value
1	Data File Name	/ Volumes/ data/ nc-7-western-fragment/ CARBON01.fid/ fid
2	Title	CARBON01
3	Origin	Varian
4	Spectrometer	inova
5	Solvent	cdcl3
6	Temperature	25.0
7	Pulse Sequence	s2pul
8	Experiment	1D
9	Probe	autox7991
10	Number of Scans	4288
11	Receiver Gain	30
12	Relaxation Delay	1.0000
13	Pulse Width	4.6125
14	Acquisition Date	2018-07-03T06:35:39
15	Spectrometer Frequency	125.65
16	Spectral Width	31446.5
17	Lowest Frequency	-1886.2
18	Nucleus	13C
19	Acquired Size	32768
20	Spectral Size	65536

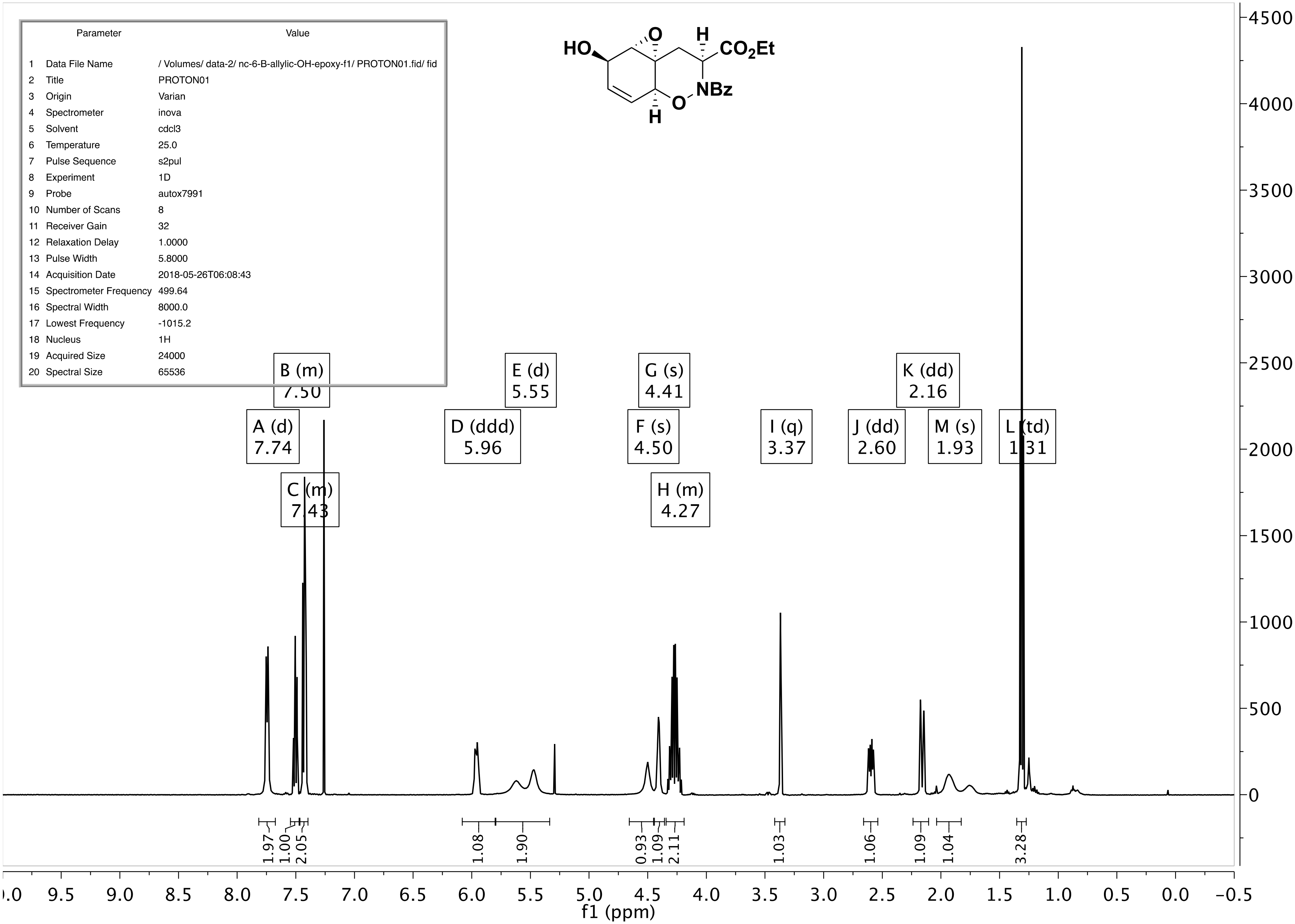

Parameter
Value
1 Data File Name / Volumes/ data-2/ nc-6-B-allylic-OH-epoxy-f1/ PROTON01.fid/ fid
2 Title PROTON01
3 Origin Varian
4 Spectrometer inova
5 Solvent cdcl3
6 Temperature 25.0
7 Pulse Sequence s2pul
8 Experiment 1D
9 Probe autox7991
10 Number of Scans 8
11 Receiver Gain 32
12 Relaxation Delay 1.0000
13 Pulse Width 5.8000
14 Acquisition Date 2018-05-26T06:08:43
15 Spectrometer Frequency 499.64
16 Spectral Width 8000.0
17 Lowest Frequency -1015.2
18 Nucleus 1H
19 Acquired Size 24000
20 Spectral Size 65536
HO
O
H
CO2Et
NBz
O
H
A (d) 7.74
B (m) 7.50
C (m) 7.43
D (ddd) 5.96
E (d) 5.55
F (s) 4.50
G (s) 4.41
H (m) 4.27
I (q) 3.37
J (dd) 2.60
K (dd) 2.16
M (s) 1.93
L (td) 1.31
1.97
1.00
2.05
1.08
1.90
0.93
1.09
2.11
1.03
1.06
1.09
1.04
3.28
f1 (ppm)

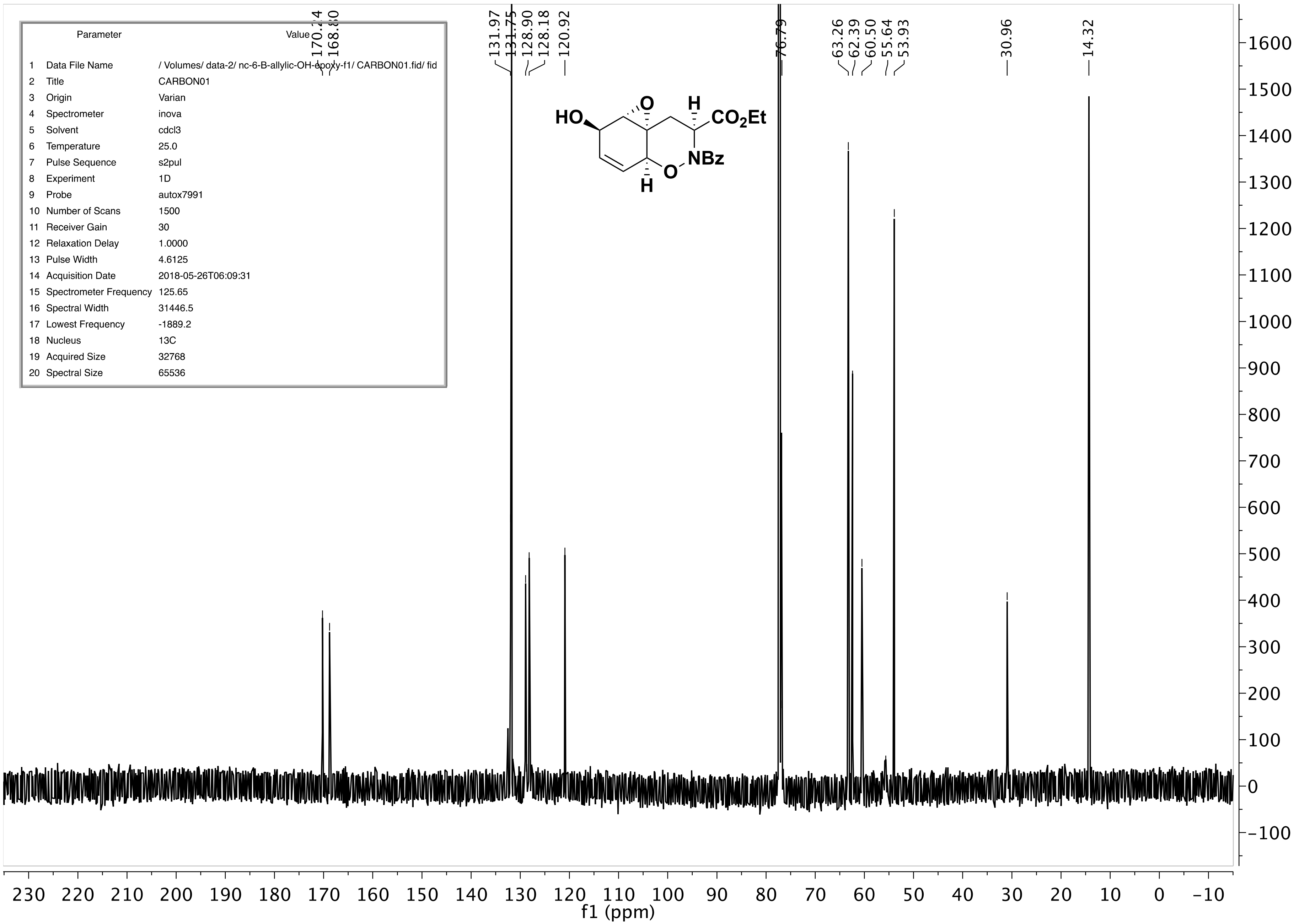
Parameter Value
1 Data File Name / Volumes/ data-2/ nc-6-B-allylic-OH-epoxy-f1/ CARBON01.fid/ fid
2 Title CARBON01
3 Origin Varian
4 Spectrometer inova
5 Solvent cdcl3
6 Temperature 25.0
7 Pulse Sequence s2pul
8 Experiment 1D
9 Probe autox7991
10 Number of Scans 1500
11 Receiver Gain 30
12 Relaxation Delay 1.0000
13 Pulse Width 4.6125
14 Acquisition Date 2018-05-26T06:09:31
15 Spectrometer Frequency 125.65
16 Spectral Width 31446.5
17 Lowest Frequency -1889.2
18 Nucleus 13C
19 Acquired Size 32768
20 Spectral Size 65536
170.24
168.80
131.97
131.75
128.90
128.18
120.92
76.79
63.26
62.39
60.50
55.64
53.93
30.96
14.32
HO
O
H
CO2Et
NBz
O
H
f1 (ppm)

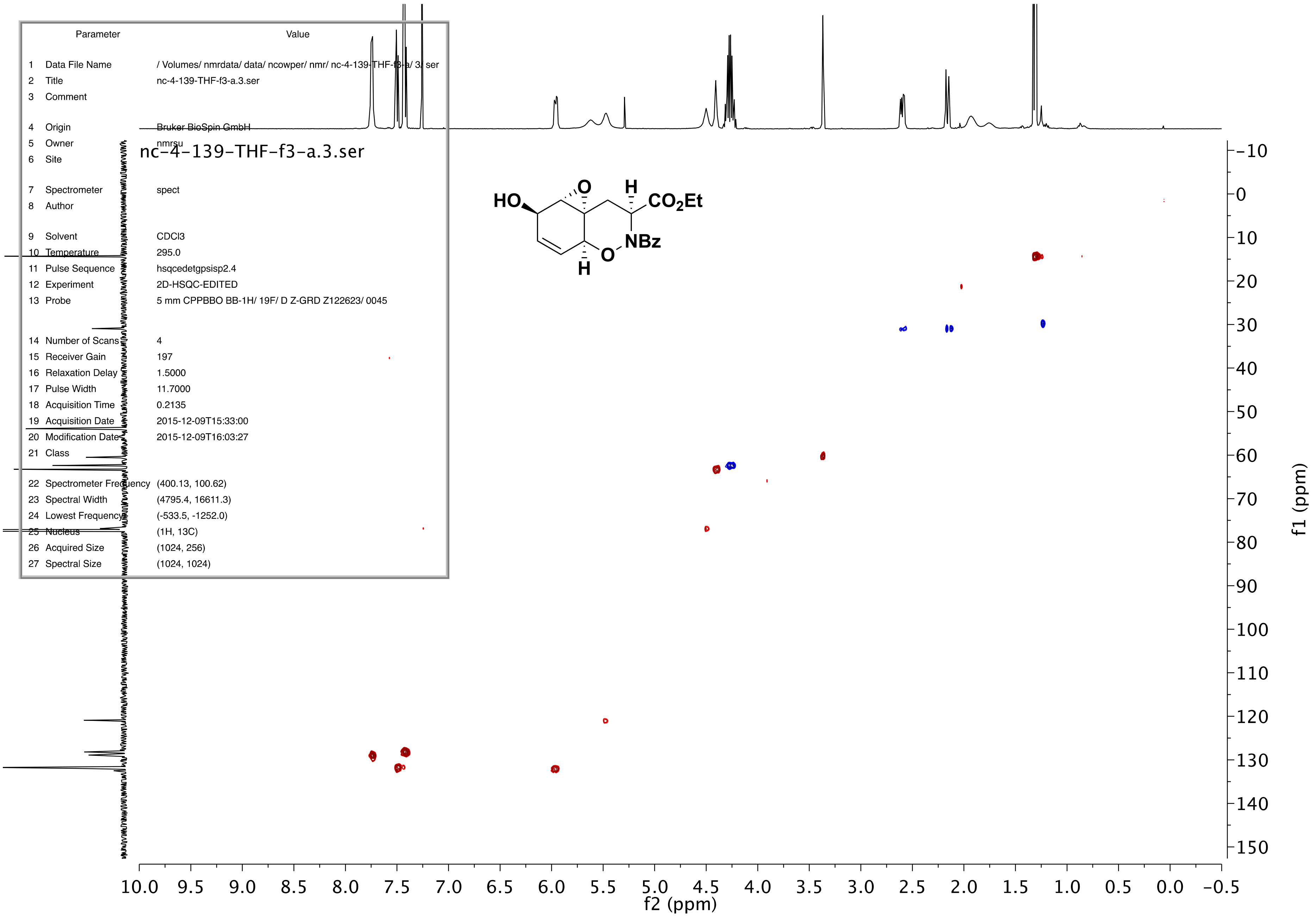

	Parameter	Value
1	Data File Name	/ Volumes/ nmrdata/ data/ ncowper/ nmr/ nc-4-139-THF-f3-a/ 3/ ser
2	Title	nc-4-139-THF-f3-a.3.ser
3	Comment	
4	Origin	Bruker BioSpin GmbH
5	Owner	nmrsu
6	Site	
7	Spectrometer	spect
8	Author	
9	Solvent	CDCl3
10	Temperature	295.0
11	Pulse Sequence	hsqcedetgpsisp2.4
12	Experiment	2D-HSQC-EDITED
13	Probe	5 mm CPPBBO BB-1H/ 19F/ D Z-GRD Z122623/ 0045
14	Number of Scans	4
15	Receiver Gain	197
16	Relaxation Delay	1.5000
17	Pulse Width	11.7000
18	Acquisition Time	0.2135
19	Acquisition Date	2015-12-09T15:33:00
20	Modification Date	2015-12-09T16:03:27
21	Class	
22	Spectrometer Frequency	(400.13, 100.62)
23	Spectral Width	(4795.4, 16611.3)
24	Lowest Frequency	(-533.5, -1252.0)
25	Nucleus	(1H, 13C)
26	Acquired Size	(1024, 256)
27	Spectral Size	(1024, 1024)

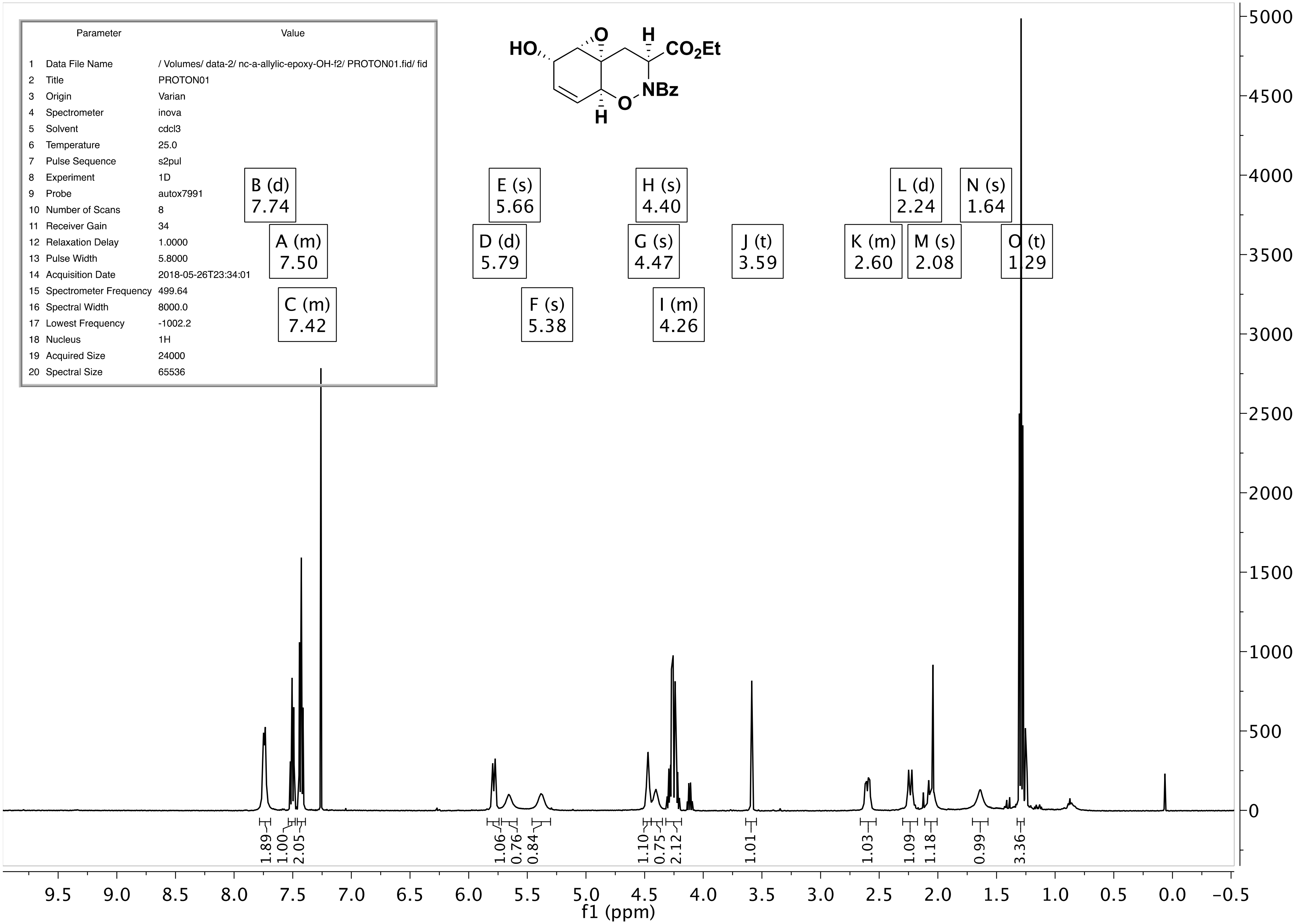
Parameter Value
1 Data File Name / Volumes/ data-2/ nc-a-allylic-epoxy-OH-f2/ PROTON01.fid/ fid
2 Title PROTON01
3 Origin Varian
4 Spectrometer inova
5 Solvent cdcl3
6 Temperature 25.0
7 Pulse Sequence s2pul
8 Experiment 1D
9 Probe autox7991
10 Number of Scans 8
11 Receiver Gain 34
12 Relaxation Delay 1.0000
13 Pulse Width 5.8000
14 Acquisition Date 2018-05-26T23:34:01
15 Spectrometer Frequency 499.64
16 Spectral Width 8000.0
17 Lowest Frequency -1002.2
18 Nucleus 1H
19 Acquired Size 24000
20 Spectral Size 65536
HO
O
H
CO2Et
NBz
O
H
B (d) 7.74
A (m) 7.50
C (m) 7.42
E (s) 5.66
D (d) 5.79
F (s) 5.38
H (s) 4.40
G (s) 4.47
I (m) 4.26
J (t) 3.59
K (m) 2.60
L (d) 2.24
M (s) 2.08
N (s) 1.64
O (t) 1.29
1.89
1.00
2.05
1.06
0.76
0.84
1.10
0.75
2.12
1.01
1.03
1.09
1.18
0.99
3.36
f1 (ppm)

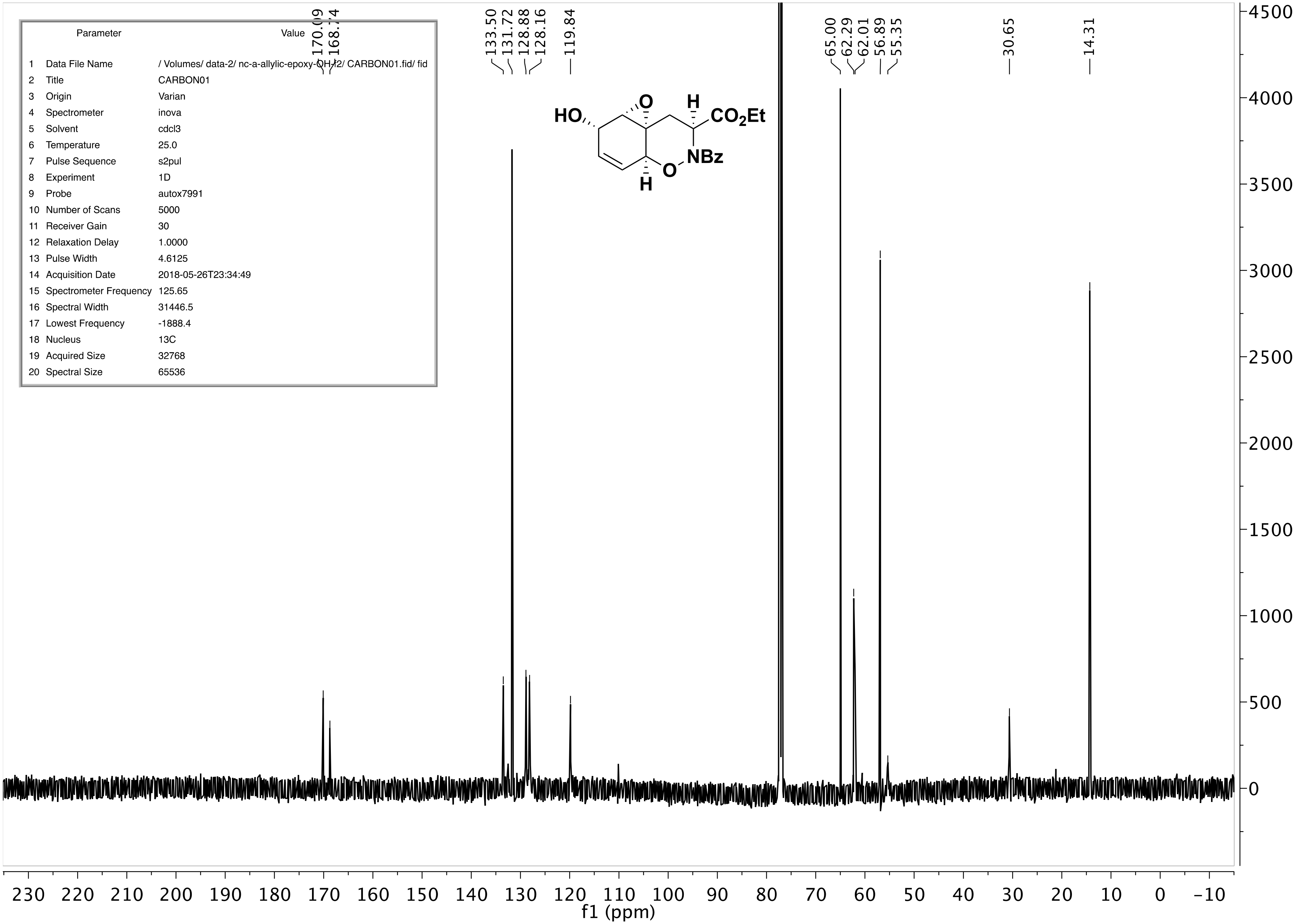
Parameter Value
1 Data File Name / Volumes/ data-2/ nc-a-allylic-epoxy-OH-2/ CARBON01.fid/ fid
2 Title CARBON01
3 Origin Varian
4 Spectrometer inova
5 Solvent cdcl3
6 Temperature 25.0
7 Pulse Sequence s2pul
8 Experiment 1D
9 Probe autox7991
10 Number of Scans 5000
11 Receiver Gain 30
12 Relaxation Delay 1.0000
13 Pulse Width 4.6125
14 Acquisition Date 2018-05-26T23:34:49
15 Spectrometer Frequency 125.65
16 Spectral Width 31446.5
17 Lowest Frequency -1888.4
18 Nucleus 13C
19 Acquired Size 32768
20 Spectral Size 65536
170.09
168.74
133.50
131.72
128.88
128.16
119.84
65.00
62.29
62.01
56.89
55.35
30.65
14.31
HO
O
H
CO2Et
NBz
O
H
f1 (ppm)

Parameter		Value
1	Data File Name	/ Volumes/ nmrdata/ data/ ncowper/ nmr/ nc-141-f6-8a/ 2/ ser
2	Title	nc-141-f6-8a.2.ser
3	Comment	
4	Origin	Bruker BioSpin GmbH
5	Owner	nmrsu
6	Site	
7	Spectrometer	spect
8	Author	
9	Solvent	CDCl3
10	Temperature	294.9
11	Pulse Sequence	hsqcedetgpsisp2.4
12	Experiment	2D-HSQC-EDITED
13	Probe	5 mm CPPBBO BB-1H/ 19F/ D Z-GRD Z122623/ 0045
14	Number of Scans	2
15	Receiver Gain	197
16	Relaxation Delay	1.5000
17	Pulse Width	11.7000
18	Acquisition Time	0.2135
19	Acquisition Date	2015-12-14T14:20:00
20	Modification Date	2015-12-14T14:35:40
21	Class	
22	Spectrometer Frequency	(400.13, 100.62)
23	Spectral Width	(4795.4, 16611.3)
24	Lowest Frequency	(-528.7, -1250.0)
25	Nucleus	(1H, 13C)
26	Acquired Size	(1024, 256)
27	Spectral Size	(1024, 1024)

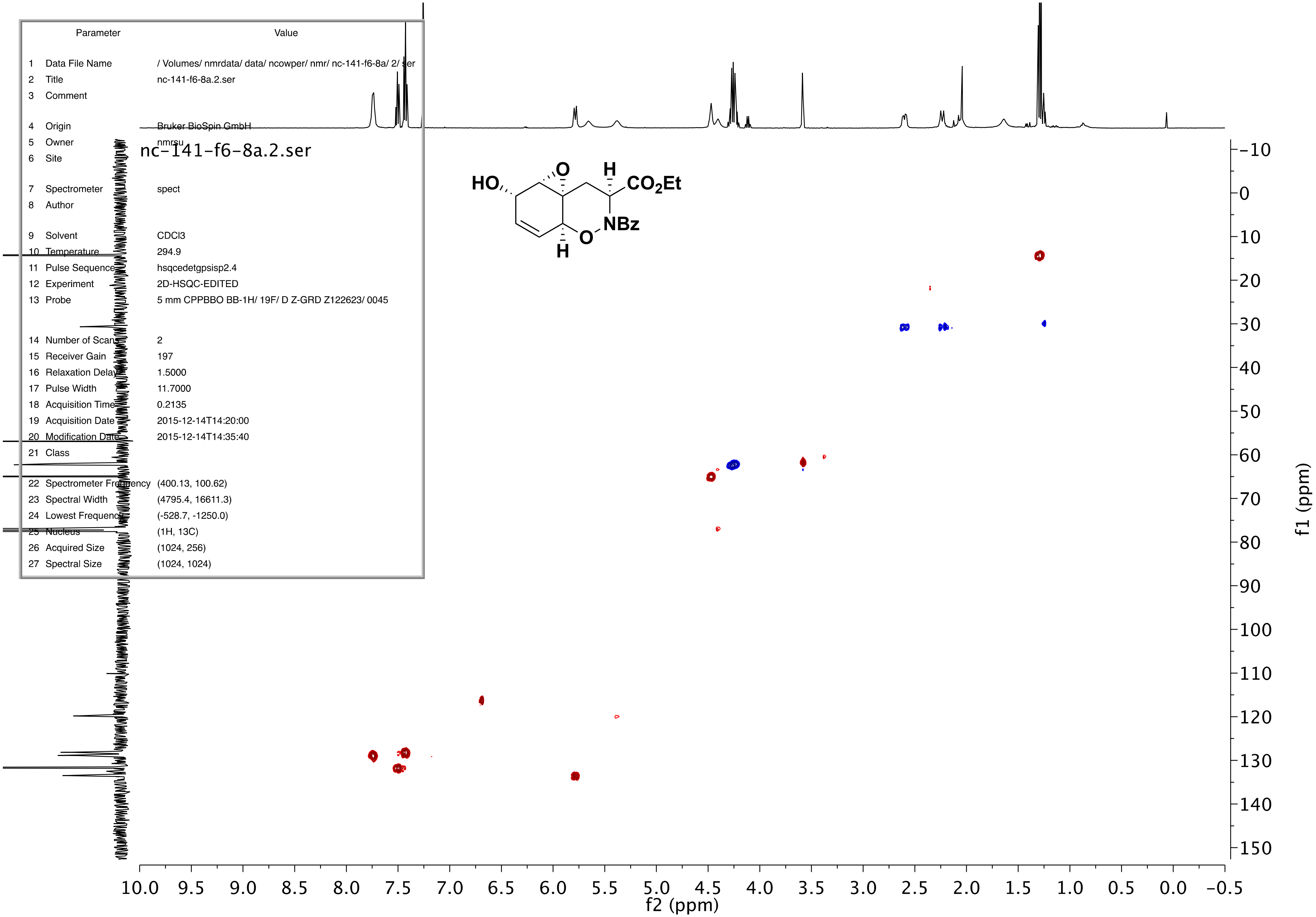

Supplement: SC-011-D0SC04802J-s001 [file SC-011-D0SC04802J-s001.pdf]
